# Structural characteristics of *ScBx* genes controlling the biosynthesis of hydroxamic acids in rye (*Secale cereale* L.)

**DOI:** 10.1007/s13353-015-0271-z

**Published:** 2015-02-10

**Authors:** Beata Bakera, Bogna Makowska, Jolanta Groszyk, Michał Niziołek, Wacław Orczyk, Hanna Bolibok-Brągoszewska, Aneta Hromada-Judycka, Monika Rakoczy-Trojanowska

**Affiliations:** 1 Department of Plant Genetics, Breeding and Biotechnology, Warsaw University of Life Sciences, 159 Nowoursynowska Str, 02-776 Warsaw, Poland; 2 Plant Breeding and Acclimatization Institute (IHAR) - National Research Institute, Radzików, 05-870 Błonie, Poland; 3 Nencki Institute of Experimental Biology, Polish Academy of Sciences, 3 Pasteur Str, 02-093 Warsaw, Poland

**Keywords:** Benzoxazinoids, Cytochrome P450 monooxygenase, Indole-glycerolphosphate lyase, *Poaceae*, Secondary metabolites

## Abstract

Benzoxazinoids (BX) are major secondary metabolites of gramineous plants that play an important role in disease resistance and allelopathy. They also have many other unique properties including anti-bacterial and anti-fungal activity, and the ability to reduce alfa–amylase activity. The biosynthesis and modification of BX are controlled by the genes *Bx1* ÷ *Bx10*, *GT* and *glu*, and the majority of these *Bx* genes have been mapped in maize, wheat and rye. However, the genetic basis of BX biosynthesis remains largely uncharacterized apart from some data from maize and wheat. The aim of this study was to isolate, sequence and characterize five genes (*ScBx1, ScBx2, ScBx3, ScBx4* and *ScBx5*) encoding enzymes involved in the synthesis of DIBOA, an important defense compound of rye. Using a modified 3D procedure of BAC library screening, seven BAC clones containing all of the *ScBx* genes were isolated and sequenced. Bioinformatic analyses of the resulting contigs were used to examine the structure and other features of these genes, including their promoters, introns and 3’UTRs. Comparative analysis showed that the *ScBx* genes are similar to those of other *Poaceae* species, especially to the *TaBx* genes. The polymorphisms present both in the coding sequences and non-coding regions of *ScBx* in relation to other *Bx* genes are predicted to have an impact on the expression, structure and properties of the encoded proteins.

**Electronic supplementary material:**

The online version of this article (doi:10.1007/s13353-015-0271-z) contains supplementary material, which is available to authorized users.

## Introduction

Benzoxazinoids (BX) are protective and allelopathic secondary metabolites found in a large number of species belonging to the *Poaceae* family, including the major agricultural cereals maize, wheat and rye (Frey et al. 2009; Niemeyer 2009). The properties and biosynthesis of BX have been intensively studied for over 50 years; they were first discovered and characterized in rye (Virtanen and Hietala 1955a, b), wheat and maize (Wahlroos and Virtanen 1959) in the 1950s. The first step in BX biosynthesis in maize, diploid and hexaploid wheat, and *Hordeum lechleri*, is the conversion of indole-3-glycerolphosphate to indole that occurs in chloroplasts. The products of the subsequent four reactions, taking place in endoplasmatic reticulum, are the four main BXs: HBOA (2-hydroxy-1,4-benzoxazin-3-one), DIBOA (2,4-dihydroxy-1,4-benzoxazin-3-one), TRIBOA (2,4,7-trihydroxy-1,4-benzoxazin-3-one) and DIMBOA (2,4-dihydroxy-7-methoxy-1,4-benzoxazin-3-one). The final products of BX biosynthesis are the glycosides, which are stored in the vacuole (Frey et al. 2009; Niemeyer 2009). Upon disintegration of the cell due to pathogen or pest attack and the mobilization of jasmonic acid and/or its methyl ester, glycosidases stored in chloroplasts are activated and toxic BX aglucons are produced (Oikawa et al. 2002; Niemeyer 2009).

DIMBOA is the main aglucon in maize and wheat, whereas in rye DIBOA and its more stable degradation product BOA (2-benzoxazolin-2(3H)-one) are predominant. DIBOA was found to be the major compound in rye leaves, while both DIMBOA and DIBOA were identified in rye roots (Frey et al. 2009; Niemeyer 2009). DIBOA-Glc is the final product of BX biosynthesis in wild *Hordeum* species and several other species of *Poaceae* (Frey et al. 2009). This compound has also been detected in leaf and root extracts of rye (Zasada et al. 2007; Meyer et al. 2009).

Several genes controlling BX biosynthesis have been isolated and characterized. The enzymes participating in BX biosynthesis in maize are encoded by the genes *ZmBx1* ÷ *ZmBx10*: *Bx1* – indole-3-glycerol phosphate lyase; *Bx2 ÷ Bx5* – cytochrome P450 monooxygenases, members of the CYP71 family; *Bx6* – 2-oxoglutarate dependent dioxygenase; *Bx7* – 7-O-methyltransferase; *Bx8, Bx9* – UDP-glucosyltransferases; *Bx10a,b,c* – 4-O-methyltransferases (Jonczyk et al. 2008; Frey et al. 2009; Meihls et al. 2013). The genes *Bx1* to *Bx5* have also been isolated from hexaploid (*Triticum aestivum*) and diploid wheat (*Triticum monococcum, Triticum urartu* and *Triticum boeoticum*), (Nomura et al. 2003; Jonczyk et al. 2008; Niemeyer 2009; Frey et al. 2009). The *Bx6* and *Bx7*, encoding enzymes that catalyze the sequential 7-hydroxylation and 7-O-methylation of DIBOA-Glc to DIMBOA-Glc, have been identified in maize, but not in wheat or rye, although the same reactions probably occur in these cereals (Frey et al. 2009; Sue et al. 2011). Indeed, the mRNA of a *Bx6-like* gene coding for 2,4-dihydroxy-1,4-benzoxazin-3-one-glucoside dioxygenase has recently been described in rye (http://www.ncbi.nlm.nih.gov/nuccore/HG380515.1÷520.1). The genes *ZmBx1 ÷ ZmBx8* are clustered and located on the short arm of maize chromosome 4, *ZmBx9/GT*, which are functionally almost identical to *ZmBx8* and *ZmBx10a,b,c* are on chromosome 1, whereas *Zmglu1* and *Zmglu2*, encoding glucosideglucosidases, are on chromosome 10. In hexaploid wheat, the *Bx* gene cluster is divided between groups: 2 – *glu* homologs, 4 – *TaBx1* and *TaBx2*, 5 – *TaBx3* ÷ *TaBx5*, and 7 – *GT* homologs (Jonczyk et al. 2008; Frey et al. 2009; Niemeyer 2009; Sue et al. 2011). The majority of *Bx* genes identified so far have been sequenced, at least at the cDNA level.

In rye, homeoloci of *TaBx1* and *TaBx2* were identified on chromosome 7R (*ScBx1* and *ScBx2*), and those of *TaBx3* ÷ *TaBx5* on chromosome 5R (*ScBx3* ÷ *ScBx5*), (Nomura et al. 2003, 2008; Frey et al. 2009; Niemeyer 2009). Recently, Sue et al. (2011) showed that two rye *Bx* genes, *ScGT*, an ortholog of *ZmBx*8/*ZmBx9*, and *Scglu*, an ortholog of *Zmglu1* and *Zmglu2*, are located on chromosomes 4R and 2R, respectively. The cDNA sequences of six *Bx* genes of rye are available: *ScBx1* ÷ *ScBx5* and a *Bx6*-like gene (La Hovary 2012, http://www.ncbi.nlm.nih.gov/nuccore/HG380515.1 ÷520.1).

The aims of this study were to examine the sequences of the full-length rye *ScBx1* ÷ *ScBx5* genes in order to characterize their exons, introns, UTRs and promoters, to compare their structures with *Bx* genes from other species, and to predict their likely role based on promoter analysis.

## Materials and methods

### Plant material and DNA isolation

DNA was isolated from young seedlings of winter rye (*Secale cereale* L.) inbred line L318 (S20–S22) using the CTAB method (Murray and Thompson 1980). BAC clone DNA was isolated using a modified alkaline lysis method and pooled using the three-dimensional (3D) procedure recommended by Amplicon Express, described below (Isolation of positive BAC clones section). The DNA concentration was measured using a NanoDrop 2000 spectrophotometer.

### Primer design and PCR

Specific primers for genes *ScBx1* and *ScBx2* were designed based on the rye cDNA and mRNA sequences (GenBank accession no. JQ716987.1 and JX442061.1, respectively), while for the other *ScBx* genes, the sequences of mRNAs of *Triticum aestivum* (B genome) were used. Rye line L318-specific primers were designed based on two selected amplicons per gene. In total, ten primer pairs were used for BAC library screening (Table 1).

**Table 1:** Primers used for BAC library screening

Gene	Sequences (5′-3′)	
KF-*ScBx1*	F	ATGGCTTTCGCGCTCAATGCGT
	R	CCGGGCGAAGGCTAATGATACAA
	F	ATGGATCCTCTTCTCGTACTACAGGCCCATC
	R	AAATGGATCCAACAGCAACAGGTTTGTCAG
KF-*ScBx2*	F	ATTTCCGGAAGGCCAGGAAG
	R	CTGAGTGAACCCCAGGACAC
	F	TGCTCAACGCCAGGAAGATT
	R	TCCTCCTAACCTCCTCCTGC
KF-*ScBx3*	F	ATCAACGTCTCCCTTGG
	R	GATGTTTCTATGCCTGCC
	F	AGAGGAGGACCTTAGCAGCAT
	R	TCATCCCTGGACAAATCCTC
KF-*ScBx4*	F	ATCATCGGCCACCTCCAC
	R	TATTGTGAGCGTCTCGGTTT
	F	CGAGCTCACCGAGATCAC
	R	CAGCACCAGGAATGATGTTT
KF-*ScBx5*	F	CGACAAGTACGGCCACAAC
	R	TTGAATCCCACGAGAAGGTC
	F	GTCGACAAGTACGGCCACA
	R	CACGAATCTTGTTGACGACGA

PCRs were composed of 500 ng total genomic DNA, 3 μM F and R primers, 0.2 mM dNTPs, 0.5 mM MgCl_2_, 1x PCR buffer and 3 units of *DreamTaq polymerase* (Fermentas) in a total volume of 15 μl. Amplification was performed in a thermal cycler using the following conditions: (1) 94 °C for 1 min; (2) 94 °C for 30 s, 60 °C for 30 s, 72 °C for 60 s for 35 cycles; (3) 72 °C for 5 min. The products were separated on a 1 % agarose gel, stained with ethidium bromide and visualized on a UV transilluminator.

### Cloning, sequencing and BLAST analysis

All amplicons were purified using a GeneJET PCR Purification Kit (Thermo Scientific) and sequenced by a commercial sequencing company (Genomed S.A., Warsaw). The resulting sequences were compared with those of all *Bx* orthologs available in databases using the BLAST algorithm.

### Construction of a BAC library

For the preparation of high molecular weight (HMW) rye DNA, nuclei were isolated and purified by flow cytometry as described by Šimková et al. (2003). Approximately 48,000 nuclei (corresponding to ca. 0.8 μg DNA) were embedded in 80 μl agarose plugs and DNA isolation was performed according to Šimková et al. (2003), except that the plugs were washed six times in ice-cold TE buffer before digestion. A BAC library was constructed as described by Peterson et al. (2000) with some modifications. Each plug was cut into nine pieces, which were divided among three tubes. For partial digestion of the HMW DNA, 0.4 to 1.2 U of *Hind*III (0.7 U on average) were used per tube. Two rounds of size selection were then performed. In the first round, the partially digested DNA was resolved on a 1 % SeaKem Gold Agarose gel (Lonza, Rockland, USA) in 0.25xTBE by pulsed-field gel electrophoresis (PFGE) using the following conditions: voltage 6 V/cm, switch time 1–50 s, run time 17 h. The size fraction of 100–150 kb was cut from the gel and subjected to a second round of electrophoretic size selection on a 0.9 % SeaKem Gold Agarose gel in 0.25xTBE using the following conditions: voltage 6 V/cm, switch time 3 s, run time 17 h. The 100–150-kb size fraction was cut from the gel and subdivided into two fractions of 100–120 kb (B) and 120–150 kb (M). The DNA in each fraction was electroeluted from the gel and quantified by electrophoresis on a standard 1 % agarose gel alongside a phage lambda DNA dilution series. The DNA from both of the fractions was ligated with *Hind*III-digested cloning-ready pIndigoBAC-5 vector (Epicentre, Madison, USA) using DNA:vector mass ratios of 3.75:1 for B and 4:1 for M. *Escherichia coli* ElectroMAX DH10B competent cells (Invitrogen, Carlsbad, USA) were then transformed with the ligations. The resulting BAC clone library, comprised of 105,216 individual clones, was ordered in 384-well plates filled with 75 μl of freezing medium (2YT supplemented with 6.6 % glycerol and 12.5 mg/l chloramphenicol) using a Qbot (Genetix, New Milton, UK) and stored at −80 °C.

### Isolation of positive BAC clones

The isolation of BAC clones containing *ScBx* genes was based on the Amplicon Express strategy (http://ampliconexpress.com/products-services/screening-services/pools-and-superpools).

Briefly, 39 superpools, each containing 2688 individual BAC clones from seven plates were prepared for the first round of PCR. The second PCR round was performed on the matrixed Plate, Row and Column pools from the selected Superpools. Finally, the “positive” BAC clones containing the desired sequences were picked and sequenced (Genomed S.A.).

### Bioinformatic analyses

Bioinformatic analyses were performed by means of the programs listed below using default settings except for SoftBerry/FGENESH when monocot plant specific gene-finding parameters and Blastn when the selection of nucleotide collection (in “other databases” section) and megablast (highly similar sequences) algorithm were applied:

- For *ScBx* genes/amplicons preliminary identification: Blastn (www.ncbi.nlm.nih.gov/)
- For constructing contigs from short amplicons after obtained in PCR during the preliminary studies: Sequencher 4.5
- For identifing *Bx* genes in the total sequence of all BAC contigs: BioEdit ver. 7.0.9.0
- For *ScBx* genes structure (exon, intron and end of 3’UTR positions within *ScBx* genes) determination: SoftBerry/FGENESH (http://www.softberry.com). Additionally, ClustalW2 (http://www.ebi.ac.uk/Tools/msa/clustalw2/) and Emboss tools: needle, seqcut, showseq (http://emboss.bioinformatics.nl/) were used as supporting tools to gene assembling
- For promoter analysis: PlantCare (http://bioinformatics.psb.ugent.be/webtools/plantcare/html/, Lescot et al. 2002). The possibly longest sequences (except for *ScBx1*, where a length of 3000 bp was arbitrarily selected) of predicted *ScBx* promoters were scanned for the presence of putative cis-acting regulatory elements. For stress-specific motifs (SSM) identifying, the “search for care” query was used. The frequency of SSM was calculated according to the formula: total number of given types of SSM divided by the total analyzed sequence upstream the start codon x 100
- For protein structure and function prediction: I-TASSER (http://zhanglab.ccmb.med.umich.edu/I-TASSER/, Zhang 2008; Roy et al. 2010). The cDNA sequences were changed into amino acid sequences and put into I-TASSER analysis. Then from five models, the highest scoring I-TASSER model, based on C-score,^1^ was chosen

Phylogenetic trees were generated separately for the cDNA sequences of the *Bx1* and *Bx2 ÷ Bx5* genes using Mega6 software (Tamura et al. 2013) based on the Maximum Parsimony algorithm (Nei and Kumar 2000) with bootstrap consensus from 2000 replicates (Felsenstein 1985). The alignments used to build phylogenetic trees were done in Mega6 software. In the first step, cDNA sequences from GenBank were translated into aminoacid sequences followed by alignment done by means of ClustalW (attached to Mega6). After that, the aminoacid sequences were transformed again into cDNA sequences and used for further phylogenetic analysis. In the case of *Bx1* gene, one out of two generated trees was chosen; for *Bx2 ÷ Bx5* genes only one tree was computed.

In the text, the *ScBx* genes isolated in this study are named **KF-** *ScBx*, while the genes isolated by La Hovary (2012) are referred to as **J-** *ScBx* and those isolated by Tanwir et al. (http://www.ncbi.nlm.nih.gov/nuccore/HG380515.1 ÷520.1) from cv. Picasso, as **HG-** *ScBx.*

## Results and discussion

Although the biosynthesis of benzoxazinoids has been intensively studied for over 50 years, little is known about the genes involved and the mechanisms controlling their differential expression in different plant tissues and during plant ontogeny. Maize, wheat and *Hordeum lechleri* are the best characterized species with regard to the genetic basis of BX synthesis. Prior to this study, the only available genetic data relating to BX synthesis in rye were the genomic location of *ScBx1 ÷ ScBx5* gene orthologs and the coding sequences of *ScBx1* and *ScBx2* genes. The construction of a BAC library (Šimková H and Rakoczy-Trojanowska 2010) enabled the isolation and precise characterization of these five genes controlling BX biosynthesis, including their introns and promoters, and permitted phylogenetic analysis.

### Isolation of positive BAC clones

Using PCR with rye KF-*ScBx1 ÷ ScBx5* gene-specific primers, seven clones were isolated from the rye inbred line L318 (S20) BAC library (105,216 clones). These clones were sequenced and the resulting reads were aligned in contigs. Altogether, 93 contigs were arranged: between 6 and 25 per BAC clone (Table 2). The desired *ScBx* sequences were identified in all selected clones. Except for clones 2 and 4, where only single *ScBx* sequences were found (*ScBx5* and *ScBx3*, respectively), two or three *ScBx* genes were present in the remaining clones. Some of the gene pairs Sc*Bx1*-*ScBx2* and *ScBx3*-*ScBx4* were present in the same contigs, whereas *ScBx5* was found in separate ones. The isolation efficiency was 0.0007 % and the presence of a given *ScBx* gene in more than one BAC clone indicated that *ScBx*s are present in the rye genome as single-copy genes. Apart from the detection of an additional *TaBx3* copy on the long arm of chromosome 5B (Nomura et al. 2003), there are no data indicating the existence of more than one copy of any *Bx1, Bx2, Bx4* and *Bx5* gene per genome.

**Table 2:** KF-*ScBx* genes identified in BAC clones

Selected BAC clone designation	Number of contigs obtained for a given BAC clone	KF-*ScBx* genes identified in a given BAC clone (contig no.)
1	11	*ScBx1* (2), *ScBx2*(4)
2	16	*ScBx5*(3)
3	13	*ScBx3*(6), *ScBx4*(6), *ScBx5*(5)
4	11	*ScBx3*(7)
5	6	*ScBx1* (1)*, ScBx2*(1)
6	25	*ScBx3* (4), *ScBx4*(4), *ScBx5*(7)
7	11	*ScBx1*(3), *ScBx2*(2)

### Characteristics of the KF-*ScBx1* ÷ *ScBx5* genes

#### Structure

**Fig. 1:**
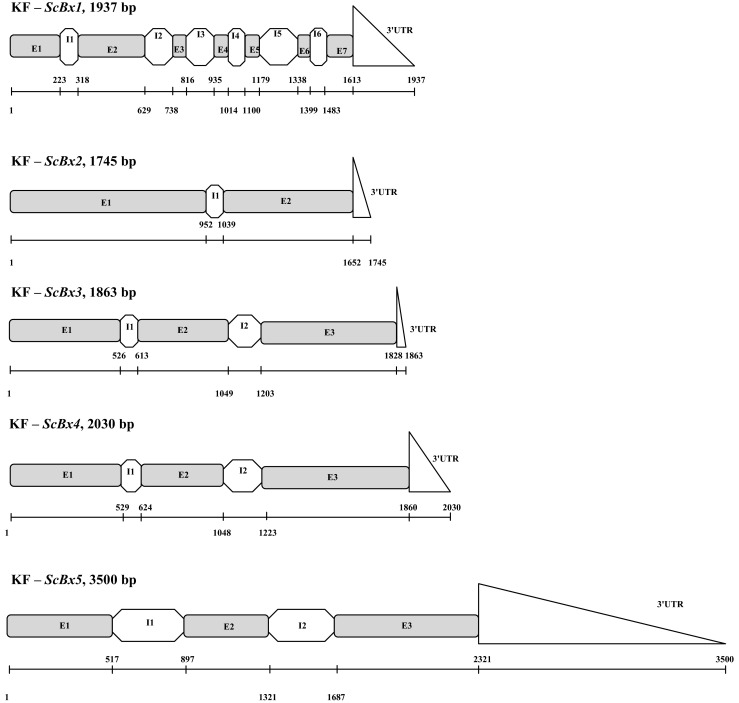
The schematic structure of KF-*ScBx* genes; E – exon, I – intron

The predicted structure and length of the five KF-*ScBx* genes are presented in Table 3 and Fig. 1. The gene *ScBx1* is composed of seven exons and six introns, *ScBx2* has two exons and one intron, and each of the genes *ScBx3* ÷ *ScBx5* has three exons and two introns. While *ScBx1* has the largest number of exons, the length of individual exons and their total length are the lowest among all the *ScBx* genes. Despite the fact that *ScBx2* contains two exons and *ScBx3* ÷ *ScBx5* have three exons each, the total exon length of these *ScBx* genes is very similar. The *ScBx5* gene has the longest sequence due to the possession of relatively long introns (more than twice longer than in the genes *ScBx2* ÷ *ScBx4*) and an unusually long 3′UTR. It was not possible to characterize the 5′UTR of the newly isolated rye *Bx* genes by bioinformatic analysis alone. The position and number of introns differ between genes KF-*ScBx2 ÷ ScBx5*: *ScBx2* has only one, most probably the ancestral intron, whereas *ScBx3*, *ScBx4* and *ScBx5* – two introns (the ancestral intron 2 and the intron 1 which was gained after the first duplication in the CYP71C subfamily as hypothesized by Dutartre et al. 2012)*.* However, their positions differ from that suggested by Dutartre et al. (2012) which calls in question the hypothesis about conserved positions of introns within *Bx2 ÷ Bx5* clades in the whole *Poaceae* family (for more details see supplementary materials, Table 4). The observation that the introns 1 and 2 are positioned identically or similarly confirms the monophyletic origin of *ScBx3 ÷ ScBx5* genes.

**Table 3:** Structural features of the KF-*ScBx* genes

KF gene name	Acc. No.(NCBI)	Gene length [bp]	Exons No./total length	Introns No./total length	Exon/intron index^*^	Length of 3′UTR
*ScBx1*	KF636828	1937	7/960	6/658	1.46	319
*ScBx2*	KF620524	1745	2/1563	1/88	17.76	94
*ScBx3*	KF636827	1863	3/1584	2/243	6.52	36
*ScBx4*	KF636826	2030	3/1587	2/272	5.84	171
*ScBx5*	KF636825	3500	3/1572	2/748	2.10	1180

^*^The total length of exons divided by the total length of introns

**Table 4:** Stress-specific motifs found in promoter sequences of KF-ScBx genes; ^*)^ only promoters of *TaBx3* and *TaBx4* were compared

Gene name	Promoter sequence length [bp]	Stress-specific motifs/No. of a given motif	Probable function	Presence/absence of SSM in wheat(+/−)^*)^ and maize (++/−−)	Frequency [No. of stress-specific motifs/100 nt]
*ScBx1*	3000	GGCAAC/1	MYBHv1 binding site	--	0.30
		CGTCA/3, TGACG/3	MeJA-responsiveness	++	
		AAAAAATTTC/1	heat stress responsiveness	--	
		CCATCTTTTT/1	salicylic acid responsiveness	++	
*ScBx2*	1960	CAACGG/1	MYBHv1 binding site	++	0.36
		CGTCA /1	MeJA-responsiveness	--	
		TGACG/1		--	
		AAAAAATTTC/1	heat stress responsiveness	++	
		CGGTCA/1	MYB binding site	++	
		ATTTTCTTCA/2	defense and stress responsiveness	++	
*ScBx3*	1497	TTGACC/2	fungal elicitor responsive element	+; −-	0.74
		CAACGG/1	MYBHv1 binding site	+; −-	
		AAAAAATTTC /1	heat stress responsiveness	-; −-	
		TAACTG/2, CAATCA/1, CGGTCA/2	MYB binding site involved in drought-inducibility	+; −-	
		ATTTTCTCCA/2	defense and stress responsiveness	-; −-	
*ScBx4*	1041	TTGACC/1	fungal elicitor responsive element	+; −-	1.15
		CGTCA/3	MeJA-responsiveness	-; ++	
		TGACG/3			
		AGAAAATTCG/1	heat stress responsiveness	+, −-	
		ATTTTCTTCA/2	defense and stress responsiveness	+; ++	
		CAGAAAAGGA/2	salicylic acid responsiveness	-; −-	
*ScBx5*	2725	CAACGG/1	MYBHv1 binding site	--	0.37
		CGTCA/3	MeJA-responsiveness	++	
		TGACG/3			
		TAACTG/1	MYB binding site involved in drought-inducibility	--	
		GTTTTCTTAC/1	defense and stress responsiveness	--	
		TCAGAAGAGG/1	salicylic acid responsiveness	++	

#### Predicted function

Use of the *ScBx* gene sequences to search the NCBI database (http://blast.ncbi.nlm.nih.gov/Blast.cgi) identified highly significant similarities between the coding sequences (formed by splicing the exons together) of the 5 KF-*ScBx* genes and the *Bx* genes of rye (HG and J sequences), *H. lechleri*, *Z. mays* and three genomes of *T. aestivum* (for more details see supplementary materials, Table 1). All of the KF genes, apart from *ScBx1*, are most similar to the rye and wheat genes, and the least similar to the maize genes. In contrast, the KF-*ScBx1* sequence is more similar to the equivalent *Z. mays* gene than to that of *H. lechleri*. A more detailed comparison is described in Comparative structural analysis of *Bx* genes subsection.

Based on the results of our bioinformatic analysis, the proteins encoded by the KF-*ScBx* genes probably have the following functions:

- *ScBx1* – indole-3-glycerol phosphate lyase
- *ScBx2* – indole monooxygenase
- *ScBx3* – indolin-2-one monooxygenase
- *ScBx4* – 3-hydroxyindolin-2-one monooxygenase
- *ScBx5* – 2-hydroxy-1,4-benzoxazin-3-one monooxygenase

### Arrangement

It was possible to establish the arrangement of gene pairs *ScBx1 ÷ ScBx2* and *ScBx3 ÷ ScBx4* since they were present in the same contig(s) of given BAC clones: *ScBx1 ÷ ScBx2* in contig 1 of clone 5, and *ScBx3 ÷ ScBx4* in contig 6 of clone 3 and contig 4 of clone 6 (Table 2). The distances (from the last nucleotide of the 3′UTR of the first gene to the first nucleotide of the first exon of the second gene in a given pair) between genes *ScBx1* and *ScBx2*, organized “tail-to-head”, is 1960 bp and between *ScBx3* and *ScBx4*, also arranged “tail-to-head”, is 8073 bp. This arrangement of these two pairs of genes is like that found in *Triticum aestivum* (Nomura et al. 2003, 2005; Frey et al. 1997; Jonczyk et al. 2008). The distance between *ScBx3* and *ScBx4* is also similar to that of the wheat orthologs (8974, 7255 and 11,309 bp in the A, B and D genomes, respectively), but considerably less than that in maize (45,767 bp). In the case of the gene pair *ScBx1 ÷ ScBx2*, it is only possible to compare the separation distance with that in maize: *ZmBx1* is 2490 bp downstream of *ZmBx2* (Frey et al. 1997). We speculate that this spacing is similar in wheat because both deletion mapping and Southern hybridization analysis revealed that *TaBx1* and *TaBx2* are located close together in the same region of each chromosome (Nomura et al. 2003), but no supporting experimental evidence is available.

Since *ScBx5* was not found in the same contig as *ScBx3* and *ScBx4*, it was not possible to determine the distances between these three genes. Because these genes were always present in the same BACs, it may be concluded that, as in wheat (Nomura et al. 2003, 2005; Sue et al. 2011), the KF-*ScBx3 ÷ ScBx5* genes are located on the same chromosome (5 RS). Although we cannot precisely determine the arm locations of KF-*ScBx3 ÷ ScBx5*, or their separation distances in the rye genome, an approximate evaluation is possible based on the ordering and lengths of contigs from BACs 3 and 6. The spaces between *ScBx5* and *ScBx3*, and between *ScBx5* and *ScBx4* are at least 2929 bp and 14,353 bp, respectively.

### Promoters

In all of the analyzed promoters, SSM potentially involved in different abiotic (e.g., heat or drought) and biotic (e.g., fungal elicitor) stress response reactions, as well as in signaling pathway mobilization (e.g., MeJa- or SA-mediated pathways), were found (Table 4). The sequences of *cis*-acting regulatory elements involved in MeJA-responsiveness (CGTCA and TGACG) were those most frequently detected. The highest frequency of SSMs was in the promoters of genes *ScBx3* and *ScBx4*: 0.74 and 1.15 SSM per 100 nt, respectively. The promoter binding sites MYB and MYBHv1 (analogous to human myeloblastosis gene family coding for transcription factor Myb), typical for the majority of stress-related gene promoters, were identified in almost all of the analyzed promoters except that of *ScBx4*.

A comparison of the KF-*ScBx* promoters with those of the genes *TaBx3*, *TaBx4* and *TaBx5* (sequences of ≥ 200 nt are only available for these three genes and *ZmBx1* ÷ *ZmBx5*, showed that the majority of SSMs are common to all three species; for more details see supplementary materials, Table 2). However, some potentially important differences were found. The *cis*-acting element involved in abscisic acid responsiveness, present in some wheat (*TaBx3*, *TaBx4*) and maize (*ZmBx1*, *ZmBx3*, *ZmBx5*) promoters, was not identified in rye. Other SSM regulatory elements involved in cold- and dehydration-responsiveness were only detected in wheat. A *cis*-acting regulatory element involved in MeJA-responsiveness was present in rye and maize, but not in wheat. These findings suggest that the analyzed rye *Bx* genes play roles in different stress response reactions and are consistent with the existing knowledge. Benzoxazinoids, except for their allelopathic potential are proved to be a crucial element in the defense mechanisms of many species belonging to the *Poaceae* family against biotic stresses as pests or pathogens (Niemeyer 2009). Their role in the defense against abiotic stresses (drought, soil salinity, triazine derivatives, aluminum) has also been documented (Makleit 2005; Kato-Noguchi 2008; Niemeyer 2009) but much more poorly and fragmentarily. In this context, an identification in *ScBx* gene promoters of the abiotic stress specific motifs is of spectacular importance. Nevertheless, the specificity of some rye *Bx* genes, especially *ScBx3* and *ScBx4*, appears to be slightly different to that of the equivalent genes of wheat and maize or rye SSMs differ from wheat/maize ones. Moreover, until the experimental evidence is available, we can only predict, on the base of promoter bioinformatic analysis, the function of *ScBx* genes.

The frequency of SSM elements in the compared promoters of the *Bx3* and *Bx4* genes was much lower in wheat and maize than in rye amounting: 0.27, 0.67, 0.24 and 0.31 for *TaBx3*, *TaBx4*, *ZmBx3 and ZmBx4*, respectively, and it was comparable in the case of *Bx5* genes (for more details see supplementary materials, Table 2a, 2b). For contrast, we have also compared the frequency of potential SSMs between *ScBx* gene promoters and the total contig sequence (806,758 bp) of seven BAC clones which included *ScBx* genes (Table 2) which turned to be considerably lower, at least fourfold, than in promoters. Some motifs — occurred either sporadically — AAAAAATTTC (for heat stress responsiveness), CCATCTTTTT and CAGAAAAGGA (for salicylic acid responsiveness), ATTTTCTTCA and ATTTTCTCCA (for defense and stress responsiveness), and some motifs: AGAAAATTCG (for heat stress responsiveness) and GTTTTCTTAC (for defense and stress responsiveness) were not present at all (for more details see supplementary materials, Table 2c).

### Comparative structural analysis of *Bx* genes

#### *ScBx* genes of different rye accessions

The availability of only incomplete data prevented full comparison of the KF-*ScBx* genes with those from accessions HG and J. The coding sequences of the genes *ScBx1* and *ScBx3* were the same in different rye accessions, but slight differences were found in the coding sequences of the other genes and in the 3′UTR lengths (Table 5).

**Table 5:** Comparison of coding sequence and 3′UTR lengths of *ScBx* genes in rye accessions KF, HG and J

Gene	KF		HG		J	
	Exon total length[bp]	3′UTR [bp]	Exon total length [bp]	3′UTR [bp]	Exon total length [bp]	3′UTR [bp]
*ScBx1*	960	319	960	na	960	330
*ScBx2*	1563	94	1587	na	1563	44
*ScBx3*	1584	36	1584	na	na	na
*ScBx4*	1587	171	1530	na	na	na
*ScBx5*	1572	1180	1575	na	na	na

*na* data not available

All *ScBx* genes from the different rye accessions were similar at the nucleotide level (for more details see supplementary materials, Table 3), but only the *ScBx3* gene sequences were identical. The other *ScBx* genes varied by the presence of a few SNPs and INDELs. The highest number of SNPs was identified in the first exon of *ScBx2*, with A/G, C/T and T/C being the most frequent polymorphisms. Of the 19 identified SNPs, three caused non-conserved and one a semi-conserved substitution. Three INDELs caused length mismatches between the *ScBx2*, *ScBx4* and *ScBx5* genes of the KF and HG accessions. These are present in the first exon of KF-*ScBx2* (24-bp deletion: ATGGCTCAGGTACATGTAGAAGAG), the first exon of KF-*ScBx4* (57-bp insertion: GCAGCGCGCCGTCGGCCATGGCGTATCCACAGAAGCACTACTGCT…CT) and the second exon of KF-*ScBx5* (3-bp deletion: TCC), (for more details see supplementary materials, Table 3).

The non-conserved amino acid (AA) substitutions and INDELs may have an impact on protein properties. To test this hypothesis, we performed bioinformatic prediction of the changes in protein structure caused by two major INDELs present in two of the KF-*ScBx* genes: a 24-bp deletion in the first exon of KF-*ScBx2* and a 57-bp insertion in the first exon of KF-*ScBx4* (for more details see supplementary materials, Fig. 1 a, b). This analysis showed that these INDELs caused changes in the length of the first predicted α-helices of KF-*ScBx2* (reduced) and KF- *ScBx4* (increased) compared with those of the corresponding proteins of accession HG. No other structural alterations were found.

### *Bx* genes in different cereal species

Maize, wheat and *Hordeum lechleri* are the best characterized species with regard to the genetic basis of BX synthesis (Frey et al. 1995, 1997, 2009; Jonczyk et al. 2008; Nomura et al. 2003, 2005; Grün et al. 2005). Therefore, we performed a comparative analysis including *Bx* genes from these species. However, in the case of wheat *Bx1*, *Bx2* and *Bx5*, and *H.lechleri Bx1* ÷ *Bx5* the number of exons and introns were established bioinformatically as only *cds* sequences of these genes are available.

The majority of sequenced *Bx* genes had the same number of exons and introns in phase 0 as their orthologs. Two exceptions are the *ZmBx1* gene with 6 exons, while this gene in all the other species has seven exons, and the *ZmBx4* gene with only two exons and one, most probably ancestral, intron rather than three exons and two introns found in the equivalent *Sc-*, *Ta-* and *HlBx* genes (Table 6). The fifth and sixth exons of the rye and wheat *Bx1* genes correspond to the fifth exon of the maize gene. The *ZmBx4* intron matches the second intron of *ScBx4*, *TaBx4* and *HlBx4* genes.

**Table 6:** Comparison of the general structure of *Bx* genes in different *Poaceae* species

Gene	Rye KF		Wheat, genome A		Wheat, genome B		Wheat, genome D		Maize		*H. lechleri*	
	Exon No.	Intron No.	Exon No.	Intron No.	Exon No.	Intron No.	Exon No.	Intron No.	Exon No.	Intron No.	Exon No.	Intron No.
*Bx1*	7	6	7	6	7	6	7	6	6	5	7	6
*Bx2*	2	1	2	1	2	1	2	1	2	1	2	1
*Bx3*	3	2	3	2	3	2	3	2	3	2	3	2
*Bx4*	3	2	3	2	3	2	3	2	2	1	3	2
*Bx5*	3	2	3	2	3	2	3	2	3	2	3	2

These findings suggest that both the sixth exon of *Sc/TaBx1* and the second intron of *Sc/TaBx4* arose after differentiation into *Panicoideae* and *Pooideae*, and are consistent with the notion of a common, monophyletic origin of the BX biosynthetic pathway (e.g., Frey et al. 1997; Dutartre et al. 2012). However, the positions of introns in matched *Bx2 ÷ Bx5* clades turned out to be highly conserved only for genes *Bx3 and Bx4* of rye and wheat. In the case of the rest of genes we have noticed smaller or bigger differences; the most considerable dissimilarities were observed in comparisons with maize (for more details see supplementary materials, Table 4). Our results do not gainsay the hypothesis about the monophyletic origin of monooxygenase encoding genes but do not fully agree with the conception that the position of introns in the CYP71C family genes indicates their fully shared evolutionary origin as suggested by Dutartre et al. (2012).

Overall, the exon lengths of the KF-*ScBx* genes were very similar to those of the equivalent *Bx* genes of wheat and *H. lechleri*, but the exons of all maize *Bx* genes are slightly longer (Table 7). The lengths of the coding sequences of the genes *Bx3* and *Bx4* are identical in rye, wheat and *H. lechleri*, and are equal to 1584 bp and 1587 bp, respectively. The greatest variation in the *Bx* genes of different species is seen in the 3′UTR sequences. In the case of the gene *ScBx5*, the length of its predicted 3′UTR exceeds those of the other *ScBx* genes by more than four times.

**Table 7:** Comparison of coding sequence (*cds*) and 3′UTR lengths of *Bx* genes in different *Poaceae* species

Gene	Rye KF		Wheat, genome A		Wheat, genome B		Wheat, genome D		Maize		*H. lechleri*	
	*cds* [bp]	3′UTR [bp]	*cds* [bp]	3′UTR[bp]	*cds* [bp]	3′UTR [bp]	*cds* [bp]	3′UTR [bp]	*cds* [bp]	3′UTR [bp]	*cds* [bp]	3′UTR [bp]
*Bx1*	960	319	960	317	960	302	957	298	1041	346	843^*)^	136
*Bx2*	1563	94	1587	184	1587	183	1587	185	1617	84	1584	179
*Bx3*	1584	36	1584	62	1584	81	1584	61	1611	68	1584	71
*Bx4*	1587	171	1587	259	1587	216	1587	204	1608	542	1587	209
*Bx5*	1572	1180	1572	265	1575	260	1572	218	1608	421	1581	94

Sequence acc. no. (from left to right): *Bx1*: KF636828, AB094060, AB124849, AB124850, X76713, AY462226*); *Bx2*: KF620524, AB042630, AB042631, AB124851, Y11368, AY462227; *Bx3*: KF636827, AB298184, AB298185, AB298186, Y11404, AY462228; *Bx4*: KF636826, AB298184, AB298185, AB298186, X81828, AY462229; *Bx5*: KF636825, AB042629, AB124856, AB124857, Y11403, AY462230; *) only a partial cds of the *Hordeum lechleri Bx1* (indole-3-glycerol phosphate lyase) is available

As well as a general structural comparison of the KF-*ScBx* genes with *TaBx*, *ZmBx* and *HlBx* genes, we performed a more detailed analysis of polymorphisms present in both the coding and regulatory (when possible) components of these genes and the probable impact of the former on the structure and properties of the encoded proteins. A complete comparison was not possible because some of the necessary data concerning the *TaBx*, and *HlBx* genes are lacking. Generally, the *ScBx* genes were the most similar to the *TaBx* genes and the least similar to the *ZmBx* genes, except for *ScBx1* which was the most different from *HlBx1* (for more details see supplementary materials, Tables 5, 6 and 7). Polymorphisms (SNPs and INDELs) were found in all *ScBx* gene components. The frequency of SNPs and INDELs ranged from 0.08–5.15 and 0.0–1.11, respectively, depending on the gene and the gene component. The number of polymorphisms was usually higher in introns and 3′UTRs (up to tenfold) than in exons, with the exception of *ScBx5* compared with *ZmBx5* when the opposite relationship was observed. The most frequently observed SNPs in the *ScBx* genes were G/A, A/G, C/T and T/C, and the rarest were A/T, T/A, G/T and T/G, independent of the gene and species with which they were compared. Numerous insertions and deletions were identified in the *ScBx* genes, usually in non-coding components, especially compared to the *ZmBx* and *HlBx* sequences. The length of some detected INDELs reached nearly 1000 bp. The longest continuous insertion (1006 bp) was present in the 3′UTR of KF-*ScBx5* gene compared with *HlBx5* and the longest deletion (166 bp), in the 3′UTR of KF-*ScBx4* gene compared with *ZmBx4* (for more details see supplementary materials, Table 7).

When the coding sequences of *Bx* genes were compared, the highest numbers of polymorphisms were found in the first and second exons of *ScBx1*, and the first and the third exons of *ScBx2 ÷ ScBx5.* The second exon of the cytochrome P450 monooxygenase coding genes (*Bx2 ÷ Bx5*) was the least polymorphic in all species.

In general, rye *Bx* genes are most structurally similar to *TaBx* genes and then to *HlBx* genes. However, relatively extensive differences at the nucleotide level, particularly in the introns, may cause variable splicing in these species. At least some of the SNP-connected conservative AA substitutions and/or INDEL-connected AA insertions/deletions are likely to influence the properties of the encoded proteins.

### Phylogenetic analysis of *Bx* genes

#### *Bx1*

**Fig. 2:**
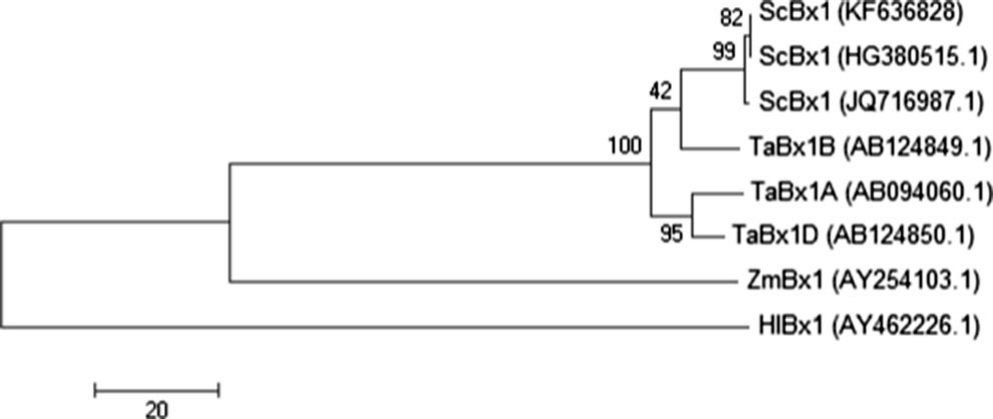
Phylogenetic tree of *Bx1* sequences. The phylogenetic tree is generated by Mega 6 software (Tamura et al. 2013) based on the Maximum Parsimony algorithm (Nei and Kumar 2000). The bootstrap values are indicated at the branch points. Scale bar indicates number of substitutions per site

**Fig. 3:**
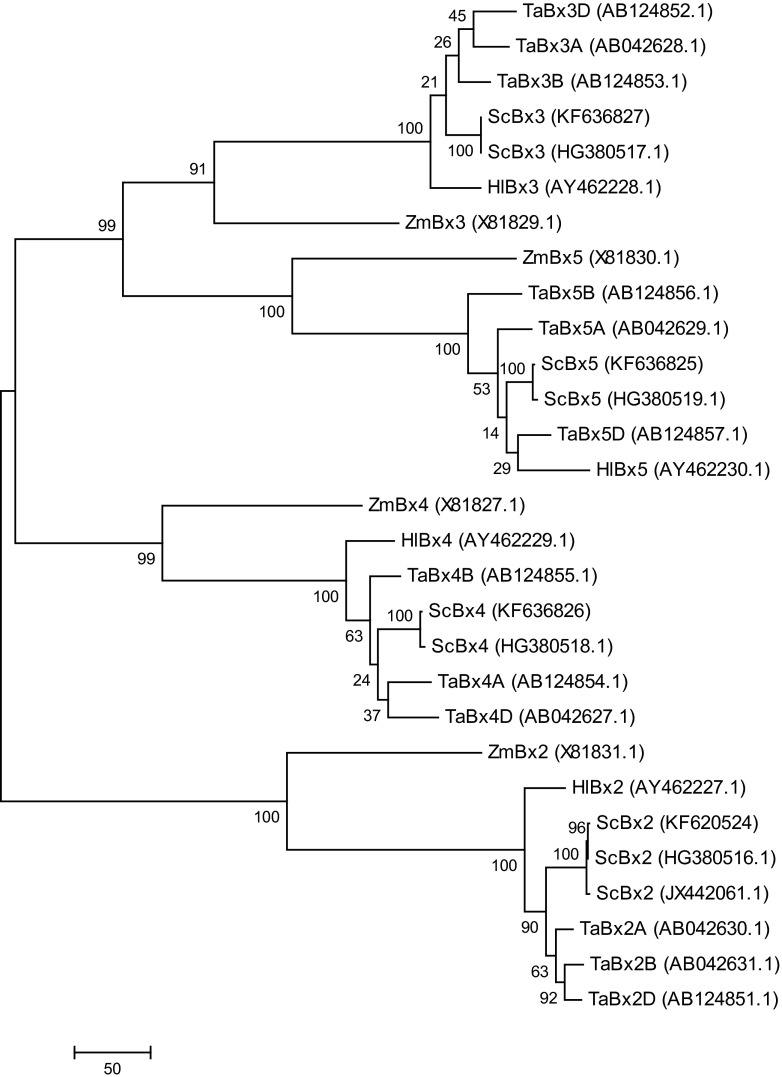
Phylogenetic tree of *Bx2÷Bx5* sequences. The phylogenetic tree is generated by Mega 6 software (Tamura et al. 2013) based on the Maximum Parsimony algorithm (Nei and Kumar 2000). The bootstrap values are indicated at the branch points. Scale bar indicates number of substitutions per site

A phylogenetic tree constructed for the *Bx1* “branchpoint” gene of *Secale cereale*, *Triticum aestivum*, *Hordeum lechleri* and *Zea mays* showed four clusters corresponding to the compared species except for *TaBx1* from B genome which was located together with *ScBx1* genes (Fig. 2). The KF-*ScBx1* gene was the closest neighbor of HG-*ScBx1*. Among the wheat genes, the *TaBx1* from genome B appeared to be the most closely related to KF-*ScBx* which was confirmed by direct sequence comparison (for more details see supplementary materials, Tables 5, 6, Fig. 3). These relationships between the analyzed species support the hypothesis of Dutartre et al. (2012) that the duplication of tryptophan synthase α (TSA) and its neofunctionalization, leading to the creation of *Bx1*, occurred before the radiation of *Poaceae* and the separation of the *Pooideae* and *Panicoideae*.

#### Bx2 ÷ Bx5

Phylogenetic analysis of the four *ScBx* genes encoding cytochrome P450s demonstrated that the newly isolated KF-*ScBx2 ÷ ScBx5* genes are closely related to the corresponding rye HG-*ScBx*s (Fig. 3). Unfortunately, their similarity to the J *-ScBx3 ÷ -ScBx5* is rather not possible to determine as the data are available only for J *-ScBx2*. In comparison with other species, these KF-*ScBx* genes generally appeared to be most similar to the corresponding wheat *TaBx* gene. A similar relationship was revealed by La Hovary (2012). As previously shown by Frey et al. (2009), each of the genes *Bx2* ÷ *Bx5* were from a separate clade, which is a strong indication that the progenitors of these genes evolved before the divergence of the *Triticeae* and the *Panicoideae*.

The results of phylogenetic analysis of *Bx* genes from three different rye accessions, wheat, maize and *H. lechleri* were generally consistent with the findings of earlier studies (Frey et al. 2009; La Hovary 2012; Dutartre et al. 2012) and support the proposed co-evolution of genes controlling the biosynthesis of BXs in the *Poaceae* family and their monophyletic origin.

## Conclusion

- A detailed analysis of the newly isolated *ScBx1 ÷ ScBx5* genes showed that they are similar to the *Bx* genes of other *Poaceae* species, both at the structural and, most probably, the functional level. However, some of the identified polymorphisms may cause slight differences in their expression specificity both on RNA and protein levels.
- The introns and 3′UTRs of *ScBx* genes (particularly *ScBx3* and *ScBx4*) characterized in this study represent gene components under strong evolutionary pressure. In spite of their regulatory role, these sequences may serve as a unique and valuable resource for genetic diversity studies or for association mapping and genomic selection.
- The presence of stress-specific DNA regulatory motifs (especially cis-acting regulatory elements involved in the MeJA-responsiveness) in the promoters of *ScBx* genes indicates their significant role in the benzoxazinoid-dependent defence strategy.

## Electronic supplementary material

Below is the link to the electronic supplementary material.

ESM 1 (DOC 638 kb)

## Footnotes

1 C-score is a confidence score for estimating the quality of predicted models by I-TASSER. It is calculated based on the significance of threading template alignments and the convergence parameters of the structure assembly simulations.
