# Supplementary material for: Structural characteristics of ScBx genes controlling the biosynthesis of hydroxamic acids in rye (Secale cereale L.)
Source: J Appl Genet. 2015 Feb 10;56(3):287–98. doi: 10.1007/s13353-015-0271-z (PMC4543422; doi:10.1007/s13353-015-0271-z)
Supplement: Supplementary file 1 — (DOC 638 kb) [file 13353_2015_271_MOESM1_ESM.doc]

Table 1. Results of bioinformatic comparative analysis of *Bx1*÷*5* genes.

| Gene KF | Parameter | Score of megablast alignment with genes: | | | | | | |
| --- | --- | --- | --- | --- | --- | --- | --- | --- |
|  |  | *Secale cereale* J | *Secale cereale* HG | *Triticum aestivum* genome A | *Triticum aestivum* genome B | *Triticum aestivum* genome D | *Hordeum lechlerii* | *Zea mays* |
| *ScBx1* | M  T  Q  E  I | 1751  1751  100  0.0  99 | 1759  1759  100  0.0  99 | 1596  1596  100  0.0  97 | 1629  1629  100  0.0  97 | 1559  1559  100  0.0  96 | 518  518  81  3e-143  75 | 827  827  82  0.0  83 |
| *ScBx2* | M  T  Q  E  I | 2787 2787  100  0.0  99 | 2805  2805  100  0.0  99 | 2563  2563  99  0.0  97 | 2560  2560  99  0.0  96 | 2560  2560  99  0.0  96 | 2466  2466  100  0.0  95 | 1200  1200  91  0.0  78 |
| *ScBx3* | M  T  Q  E  I | X | 2926  2926  100  0.00  100 | 2571  2571  100  0.00  96 | 2593  2593  100  0.00  96 | 2566  2566  100  0.00  96 | 2521  2521  100  0.00  95 | 977  977  89  0.00  79 |
| *ScBx4* | M  T  Q  E  I | X | 2750  2809  96  0.00  99 | 2555  2555  100  0.00  96 | 2604  2604  100  0.00  96 | 2527  2527  100  0.00  95 | 2460  2460  100  0.00  95 | 1088  1088  99  0.00  79 |
| *ScBx5* | M  T  Q  E  I | X | 2821  2821  100  0.00  99 | 2560  2560  100  0.00  96 | 2419  2419  100  0.00  94 | 2574  2574  100  0.00  96 | 2455  2455  100  0.00  94 | 1254  1254  98  0.00  78 |

M - Max score

T - Total score

Q - Query cover [%]

E - E-value

I - Identity [%]

X - data not available

Table 2a. Stress-specific motifs found in promoter sequences of *TaBx* genes from genome A.

| Gene name | Promoter sequence length [bp] | Stress-specific motifs/No of a given motif | function | Frequency  [No of stress-specific motifs/100 nt] |
| --- | --- | --- | --- | --- |
| *TaBx3* | 1497 | CGTACGTGCA/1 | cis-acting element involved in the abscisic acid responsiveness | 0,27 |
|  |  | ATTTTCTTCA/1 | cis-acting element involved in defense and stress responsiveness |  |
|  |  | TAACTG/1 | MYB binding site involved in drought-inducibility |  |
|  |  | CAACGG/1 | MYBHv1 binding site |  |
| *TaBx4* | 1041 | TACGTG/1 | cis-acting element involved in the abscisic acid responsiveness | 0,67 |
|  |  | TGGCCGAC/1 | regulatory element involved in cold- and dehydration-responsiveness |  |
|  |  | AGAAAATTCG/1 | cis-acting element involved in heat stress responsiveness |  |
|  |  | ATTTTCTTCA/1  GTTTTCTTAC/1 | cis-acting element involved in defense and stress responsiveness |  |
|  |  | TAACTG/1 | MYB binding site involved in drought-inducibility |  |
| *TaBx5* | 276 | CAACTG/1 | MYB binding site involved in drought-inducibility | 0,37 |

Table 2b. Stress-specific motifs found in promoter sequences of *ZmBx* genes.

| Gene name | Promoter sequence length [bp] | Stress-specific motifs/No of a given motif | function | Frequency  [No of stress-specific motifs/100 nt] |
| --- | --- | --- | --- | --- |
| *ZmBx1* | 2006 | TACGTG/1 | cis-acting element involved in the abscisic acid responsiveness | 0,40 |
|  |  | TGACG/1 | cis-acting regulatory element involved in the MeJA-responsiveness |  |
|  |  | CGTCA/1 | cis-acting regulatory element involved in the MeJA-responsiveness |  |
|  |  | ATTTTCTCCA/1 | cis-acting element involved in defense and stress responsiveness |  |
|  |  | GAGAAGAATA/1  TCAGAAGAGG//1  CCATCTTTTT/2 | cis-acting element involved in salicylic acid responsiveness |  |
| *ZmBx2* | 4539 | CACGTG/1  CGCACGTG/1  GCCGCGTGG/1  CACGTG/1 | cis-acting element involved in the abscisic acid responsiveness | 0,22 |
|  |  | CAACGG/1 | MYBHv1 binding site |  |
|  |  | AAAAAATTTC/2  AGAAAATTCG/1 | cis-acting element involved in heat stress responsiveness |  |
|  |  | CGGTCA/1 | MYB Binding Site |  |
|  |  | ATTTTCTTCA/1 | cis-acting element involved in defense and stress responsiveness |  |
| *ZmBx3* | 406 | TACGTG/1 | cis-acting element involved in the abscisic acid responsiveness | 0,25 |
| *ZmBx4* | 2604 | CGTCA/1 | cis-acting regulatory element involved in the MeJA-responsiveness | 0,31 |
|  |  | TAACTG/1 | MYB Binding Site |  |
|  |  | ATTTTCTTCA/3 | cis-acting element involved in defense and stress responsiveness |  |
|  |  | TCAGAAGAGG/1  GAGAAGAATA/1 | cis-acting element involved in salicylic acid responsiveness |  |
|  |  | TGACG/1 | cis-acting regulatory element involved in the MeJA-responsiveness |  |
| *ZmBx5* | 1967 | TACGTG/1  TACGTG/1  CGTACGTGCA/1 | cis-acting element involved in the abscisic acid responsiveness | 0,51 |
|  |  | CGTCA/2 | cis-acting regulatory element involved in the MeJA-responsiveness |  |
|  |  | AAAAAATTTC/1 | cis-acting element involved in heat stress responsiveness |  |
|  |  | CCATCTTTTT/1  GAGAAGAATA/1 | cis-acting element involved in salicylic acid responsiveness |  |
|  |  | TGACG/2 | cis-acting regulatory element involved in the MeJA-responsiveness |  |

Table 2c. Stress-specific motifs found in 7 BACs (806758 bp) containing *ScBx* gene sequences.

| Stress-specific motifs/  No. of a given motif | Probable function | Frequency  [No. of stress-specific motifs/100 nt] |
| --- | --- | --- |
| GGCAAC/152  CAACGG/162 | MYBHv1 binding site | 0,0188  0,0200 |
| CGTCA/501  TGACG/576 | MeJA-responsiveness | 0,0621  0,0714 |
| AAAAAATTTC/2  AGAAAATTCG/0 | heat stress responsiveness | 0,0002  0,0000 |
| CCATCTTTTT/2  CAGAAAAGGA/1  TCAGAAGAGG/2 | salicylic acid responsiveness | 0,0002  0,0001  0,0002 |
| CGGTCA/117 | MYB binding site | 0,0145 |
| TAACTG/119  CAATCA/228 | MYB binding site involved in drought-inducibility | 0,148  0,283 |
| ATTTTCTTCA/2  ATTTTCTCCA/2  GTTTTCTTAC/0 | defense and stress responsiveness | 0,0002  0,0002  0,0000 |
| TTGACC/162 | fungal elicitor responsive element | 0,0200 |

Table 3. SNP and INDEL polymorphisms in *ScBx* genes from rye accessions KF, J and HG.

| Gene | Gene component | SNP type – position^*)^ | SNP-associated AA substitution type – position^**)^ | INDEL  length [bp] – position^*)^ | INDEL associated AA INDEL position^**)^  /characteristics | Total no. of polymorphisms |
| --- | --- | --- | --- | --- | --- | --- |
| KF-*ScBx1* | Exon 1 | C/Y_HG_ – 109 | Pro/X – 37 | nf | - | SNP – 4  INDEL – 0 |
|  | Exon 2 | nf | - | nf | - |  |
|  | Exon 3 | nf | - | nf | - |  |
|  | Exon 4 | A/G_J_ – 651 | ns | nf | - |  |
|  | Exon 5 | nf | - | nf | - |  |
|  | Exon 6 | nf | - | nf | - |  |
|  | Exon 7 | G/A_J_ – 834 | ns | nf | - |  |
|  | 3’UTR | C/A_J_; C/T_J_ | - | nf | - |  |
| KF-*ScBx2* | Exon 1 | G/A_J_ – 42  G/T_HG_ – 76  C/T_J_ – 272  C/T_J_ – 444  C/G_J,HG_ –575  A/G_J,HG_ – 879  T/C_J_ – 881 | ns  Ala/Ser^nc^ – 26  ns  ns  ns  ns  Val/Ala^sc^– 294 | 24 _HG_ –  1 - 23 | 1 - 8/ deletion of 8 AAs: Met, Ala, Gln, Val, His, Val, Glu, Glu | SNP – 9  INDEL – 1 |
|  | Exon 2 | T/C_J_ –1224 | ns | nf | - |  |
|  | 3’UTR | T/C_J_ | - | nf | - |  |
| KF-*ScBx3* | Exon 1 | nf | - | nf | - | SNP – 0  INDEL –0 |
|  | Exon 2 | nf | - | nf | - |  |
|  | Exon 3 | nf | - | nf | - |  |
|  | 3’UTR | np | - | np | - |  |
| KF-*ScBx4* | Exon 1 | nf | - | 57_HG_ –  29 - 85 | 10 - 29/ insertion of 19 AAs: Leu, Gln, Arg, Ala, Val, Gly, His, Gly, Val, Ser, Thr, Glu, Ala, Leu, Leu, Leu, Thr, Val, Leu | SNP – 5  INDEL – 1 |
|  | Exon 2 | A/G_HG_ – 565  T/C_HG_ – 566  A/G_HG_ – 597  T/C_HG_ – 631 | ns  Met/Ala^nc^–189  ns  Cys/Arg^nc^–211 | nf | - |  |
|  | Exon 3 | C/T_HG_ – 1338 | ns | nf | - |  |
|  | 3’UTR | np | - | np | - |  |
| KF-*ScBx5* | Exon 1 | G/T_HG_ – 156 | ns | nf | - | SNP – 1  INDEL – 1 |
|  | Exon 2 | nf | - | 3_HG_ –  763 - 765 | 255/ deletion of 1 AA: Leu |  |
|  | Exon 3 | nf | - | nf | - |  |
|  | 3’UTR | np | - | np | - |  |

^*)^ in relation to the first nucleotide of the first codon

^**)^ in relation to the first AA

J – SNP/INDEL in relation to J-*ScBx*

HG – SNP/INDEL in relation to HG-*ScBx*

ns – no substitution

sc – semi-conserved substitution

nc – non-conserved substitution

nf – not found in databases

np – comparison not possible – no 3’UTR present in HG accessions

Table 4. Positions of introns in genes *Bx2*÷*Bx5* of rye, wheat and maize^*)^.

| Gene | Intron | Species | | | | |
| --- | --- | --- | --- | --- | --- | --- |
|  |  | Rye | Wheat | | | Maize |
|  |  |  | Genome A | Genome B | Genome D |  |
| *Bx2* | 1 | np^**)^ | na^***)^ | na | na | np |
|  | 2 | 952 | na | na | na | 997 |
| *Bx3* | 1 | 526 | 526 | 526 | 526 | 541 |
|  | 2 | 1049 | 1049 | 1047 | 1049 | 1132 |
| *Bx4* | 1 | 529 | 529 | 529 | 529 | np |
|  | 2 | 1048 | 1046 | 1043 | 1039 | 967 |
| *Bx5* | 1 | 517 | na | na | na | 535 |
|  | 2 | 1321 | na | na | na | 1072 |

^*)^ counting from the first nucleotide (A) of the first exon

^**)^ not present

^***)^ data not available

Table 5. Number of SNP and INDEL polymorphisms computed in KF-*ScBx* against *Bx* genes of wheat, maize and *H.lechlerii*.

| KF-*ScBx* | Gene compo-nent | vs *TaBx* (A) | | *vs TaBx* (B) | | *vs* *TaBx* (D) | | *vs* *ZmBx* | | *vs HlBx* | |
| --- | --- | --- | --- | --- | --- | --- | --- | --- | --- | --- | --- |
|  |  | NT SNP/  INDEL | AA SNP/  INDEL | NT SNP/  INDEL | AA SNP/  INDEL | NT SNP/  INDEL | AA SNP/  INDEL | NT SNP/  INDEL | AA SNP/  INDEL | NT SNP/  INDEL | AA SNP/  INDEL |
| *ScBx1* | Intron 1 | n.a. |  | n.a. |  | n.a. |  | 42/6 |  | n.a. |  |
|  | Intron 2 | n.a. |  | n.a. |  | n.a. |  | 24/11 |  | n.a. |  |
|  | Intron 3 | n.a. |  | n.a. |  | n.a. |  | 23/9 |  | n.a. |  |
|  | Intron 4 | n.a. |  | n.a. |  | n.a. |  | 35/9 |  | n.a. |  |
|  | Intron 5 | n.a. |  | n.a. |  | n.a. |  | 38/4 |  | n.a. |  |
|  | Intron 6 | n.a. |  | n.a. |  | n.a. |  | - ^*)^ |  | n.a. |  |
|  | Exon1 | 11/0 | 10/0 | 10/0 | 7/0 | 15/1 | 13/1 | 53/9 | 59/5 | 25/6 | 79/3 |
|  | Exon 2 | 11/0 |  | 8/0 |  | 9/0 |  | 52/1 |  | 79/2 |  |
|  | Exon 3 | 1/0 |  | 1/0 |  | 1/0 |  | 9/2 |  | 24/0 |  |
|  | Exon 4 | 2/0 |  | 2/0 |  | 1/0 |  | 10/2 |  | 21/0 |  |
|  | Exon 5 | 0/0 |  | 1/0 |  | 2/0 |  | 25/1 |  | 24/0 |  |
|  | Exon 6 | 1/0 |  | 1/0 |  | 1/0 |  | - ^**)^ |  | 13/0 |  |
|  | Exon 7 | 4/0 |  | 1/0 |  | 4/0 |  | 27/0 |  | 18/0 |  |
|  | 3’UTR | 37/65 |  | 40/5 |  | 30/6 |  | 203/6 |  | 90/2 |  |
| *ScBx2* | Intron 1 | n.a. |  | n.a. |  | n.a. |  | 34/9 |  | n.a. |  |
|  | Exon 1 | 32/0 | 16/1 | 34/0 | 15/1 | 33/0 | 13/1 | 184/13 | 100/6 | 37/0 | 16/2 |
|  | Exon 2 | 25/0 |  | 24/0 |  | 25/0 |  | 141/1 |  | 34/0 |  |
|  | 3’UTR | 7/3 |  | 7/3 |  | 8/3 |  | 45/7 |  | 2/3 |  |
| *ScBx3* | Intron1 | 10/0 |  | 30/2 |  | 8/0 |  | 46/3 |  | n.a. |  |
|  | Intron 2 | 39/5 |  | 27/7 |  | 44/5 |  | 73/5 |  | n.a. |  |
|  | Exon 1 | 20/0 | 15/0 | 20/0 | 15/0 | 23/0/ | 14/0 | 98/5 | 126/7 | 23/0 | 25/0 |
|  | Exon 2 | 17/0 |  | 17/0 |  | 15/0 |  | 95/3 |  | 19/0 |  |
|  | Exon 3 | 27/0 |  | 23/0 |  | 27/0 |  | 126/4 |  | 31/0 |  |
|  | 3’UTR | 8/3 |  | 5/2 |  | 4/2 |  | 17/4 |  | 16/2 |  |
| *ScBx4* | Intron1 | 27/3 |  | 29/3 |  | 26/4 |  | -^***)^ |  | n.a. |  |
|  | Intron 2 | 25/3 |  | 26/2 |  | 33/2 |  | 51/4 |  | n.a |  |
|  | Exon 1 | 23/0 | 13/0 | 23/0 | 8/0 | 24/0 | 13/0 | 96/4 | 110/5 | 33/0 | 21/0 |
|  | Exon 2 | 16/0 |  | 13/0 |  | 21/0 |  | 213/4 |  | 18/0 |  |
|  | Exon 3 | 29/0 |  | 23/0 |  | 28/0 |  | -^****)^ |  | 34/0 |  |
|  | 3’UTR | 8/2 |  | 45/6 |  | 8/2 |  | 88/6 |  | 28/7 |  |
| *ScBx5* | Intron1 | n.a. |  | n.a. |  | n.a. |  | 50/18  39/14 |  | n.a. |  |
|  | Intron 2 | n.a. |  | n.a. |  | n.a. |  | 39/14 |  | n.a. |  |
|  | Exon 1 | 26/1 | 14/0 | 46/1 | 21/1 | 28/0 | 11/0 | 101/5 | 109/7 | 27/1 | 15/2 |
|  | Exon 2 | 11/0 |  | 11/0 |  | 5/0 |  | 86/1 |  | 23/0 |  |
|  | Exon 3 | 24/0 |  | 35/0 |  | 24/0 |  | 137/4 |  | 31/0 |  |
|  | 3’UTR | 26/0 |  | 25/1 |  | 21/2 |  | 74/10 |  | 10/3 |  |

NT SNP/INDEL - SNP/INDEL on nucleotide level

AA SNP/INDEL - SNP connected AA substitution/INDEL connected AA INDEL

n.a. – comparison not possible as data are not available

^*)^ intron 6 is not present in maize

^**)^ exon 6 is not present in maize

^***)^ intron 2 is not present in maize

^****)^ exon 3 is not present in maize

Table 6. Frequency of SNP and INDEL polymorphisms in KF-*ScBx* genes in comparison with the *Bx* genes of wheat, maize and *H.lechlerii*.

| Gene KF-*ScBx* | Gene component | No. of polymorphisms per 10 nt | | | | | | | | | |
| --- | --- | --- | --- | --- | --- | --- | --- | --- | --- | --- | --- |
|  |  | vs *TaBx* (A) | | *vs TaBx* (B) | | *vs* *TaBx* (D) | | *vs* *ZmBx* | | *vs HlBx* | |
|  |  | SNP | INDEL | SNP | INDEL | SNP | INDEL | SNP | INDEL | SNP | INDEL |
| *ScBx1* | Exons | 0.31 | 0.00 | 0.25 | 0.00 | 0.34 | 0.01 | 1.83 | 0.16 | 2.02 | 0.08 |
|  | Introns | np | np | np | np | np | np | 2.46 | 0.59 | np | np |
|  | 3’UTRR | 0.50 | 0.16 | 0.82 | 0.16 | 0.50 | 0.16 | 0.50 | 0.16 | np | np |
| *ScBx2* | Exons | 0.36 | 0.00 | 0.37 | 0.00 | 0.37 | 0.00 | 2.08 | 0.08 | 0.45 | 0.00 |
|  | Introns 1 | np | np | np | np | np | np | 3.86 | 1.02 | np | np |
|  | 3’UTR | 0.74 | 0.32 | 0.74 | 0.32 | 0.85 | 0.32 | 4.79 | 0.74 | 0.21 | 0.32 |
| *ScBx3* | Exons | 0.40 | 0.00 | 0.38 | 0.00 | 0.41 | 0.00 | 2.01 | 0.08 | 0.46 | 0.00 |
|  | Introns 2 | 2.02 | 0.21 | 2.35 | 0.37 | 2.14 | 0.21 | 4.90 | 0.33 | np | np |
|  | 3’UTR | 2.22 | 0.83 | 1.39 | 0.56 | 1.11 | 0.56 | 4.72 | 1.11 | 4.44 | 0.56 |
| *ScBx4* | Exons | 0.43 | 0.00 | 0.37 | 0.00 | 0.46 | 0.00 | 1.95 | 0.05 | 0.54 | 0.00 |
|  | Introns 2 | 1.91 | 0.22 | 2.02 | 0.18 | 2.17 | 0.22 | 1.91 | 0.15 | np | np |
|  | 3’UTR | 0.47 | 0.12 | 2.63 | 0.35 | 0.47 | 0.12 | 5.15 | 0.35 | 1.64 | 0.41 |
| *ScBx5* | Exons | 0.39 | 0.01 | 0.59 | 0.01 | 0.36 | 0.00 | 2.06 | 0.06 | 0.52 | 0.01 |
|  | Introns 2 | np | np | np | np | np | np | 0.90 | 0.40 | np | np |
|  | 3’UTR | 0.22 | 0.00 | 0.21 | 0.01 | 0.18 | 0.02 | 0.35 | 0.20 | 0.08 | 0.03 |

np – comparison not possible because data concerning gene component not available

Table 7a. The structural comparison of KF-*ScBx1* with *TaBx1(A,B,D)*, *ZmBx1* and *HlBx1* genes
in exons/introns/3’UTR.

| **In KF-*ScBx1*** | *vs TaBx1A* | *vs TaBx1B* | *vs TaBx1D* | *vs ZmBx1* | *HlBx1* |
| --- | --- | --- | --- | --- | --- |
| The most/least frequent SNPs [%] | G/A(23,3)/  T/A(0,0) | G/A(25,0)/  T/A(0,0) | G/A(24,2)/  T/A(0,0) | A/G (15,3)/  T/A(1,5) | A/G (19,6)/  T/C; A/T; G/T (1,8) |
| The longest insertion (bp) | none/no data/4bp | none/no data/16bp | 3bp/no data/20bp | 3bp/25bp/4bp | 111bp/no data/86bp |
| The longest deletion (bp) | none/no data/4bp | none/no data/4bp | none/no data/4bp | 23bp/18bp/13bp | 9bp/no data/none |
| The largest number of insertions in exons | 0 | 0 | 0 | 8 | 2 |
| The largest number of deletions in exons | 0 | 0 | 1 | 1 | 7 |
| The most polymorphic exon | 1, 2 | 1 | 1 | 1 | 2 |
| The most frequent AA substitution type [%] | T/P (20,0) | S/G (28,6) | T/P (15,4)  G/R (15,4) | I/V (6,8) | I/V (5,1)  P/A (5,1) |
| Type (frequency [%]) of non-conservative AA substitutions | G/R (0,3);  E/G (0,3) | G/R (0,3);  P/L (0,3) | D/V (0,3);  R/W (0,3);  G/R (0,6) | A/E (5,1);  L/P (3,4);  R/T (3,4);  K/A (3,4);  Y/L (1,7);  P/L (1,7);  G/T (1,7);  N/A (1,7);  L/A (1,7);  V/P (1,7);  T/Q (1,7);  V/R (1,7);  S/V (1,7);  E/A (1,7);  G/Q (1,7); | E/A (3,8);  A/E (2,5);  L/A (2,5);  R/T (1,3);  N/A (1,3);  V/P (1,3);  I/P (1,3);  K/V (1,3);  V/D (1,3);  R/L (1,3);  C/L (1,3);  C/F (1,3);  Y/S (1,3);  R/S (1,3);  C/Q (1,3);  H/S (1,3);  W/R (1,3);  K/I (1,3);  I/T (1,3);  E/P (1,3);  E/G (1,3);  T/R (1,3);  L/P (1,3);  G/E (1,3); |

Table 7b. The structural comparison of KF-*ScBx2* with *TaBx2(A,B,D)*, *ZmBx2* and *HlBx2* genes
in exons/introns/3’UTR.

| **In KF-*ScBx2*** | *vs TaBx2A* | *vs TaBx2B* | *vs TaBx2D* | *vs ZmBx2* | *HlBx2* |
| --- | --- | --- | --- | --- | --- |
| The most/least frequent SNPs [%] | G/A (26,3)/  C/T; G/T (0,0) | G/A (27,6)/  A/T (0,0) | G/A (27,6)/  G/T (0,0) | A/G (19,7)/  G/T (1,2) | G/A (19,7)/  G/T (0,0) |
| The longest insertion (bp) | none/no data/none | none/no data/none | none/no data/none | 3bp/20bp/12bp | 15bp/no data/1bp |
| The longest deletion (bp) | 24bp/no data/88bp | 24bp/no data/87bp | 24bp/no data/89bp | 33bp/none/5bp | 24bp/no data/86bp |
| The largest number of insertions in exons | 1 | 1 | 1 | 11 | 1 |
| The largest number of deletions in exons | none | none | none | 2 | 1 |
| The most polymorphic exon | 1 | 1 | 1 | 1 | 1 |
| The most frequent AA substitution type [%] | V/I (18,8) | V/I (20,0) | V/I (23,1) | A/T (5,1) | V/I (18,8) |
| Type (frequency [%]) of non-conservative AA substitutions | E/V (6,3);  V/P (6,3);  E/A (6,3);  M/A (6,3) | L/Q (6,7);  V/P (6,7);  M/A (6,7) | V/P (7,7);  M/A (7,7) | A/Q (1,0)  L/P (1,0)  I/Y (1,0)  K/A (1,0)  D/A (2,0)  T/M (1,0)  F/S (2,0)  Q/A (1,0)  R/S (1,0)  L/G (1,0)  A/R (1,0)  Q/M (1,0)  L/Q (1,0)  S/R (1,0)  C/R (1,0)  L/D (1,0)  D/Y (1,0)  S/L (1,0)  N/A (1,0)  Y/D (1,0)  T/E (1,0)  Q/V (1,0)  E/A (1,0)  V/Q (1,0)  M/A (1,0) | E/G (6,3);  V/G (6,3) |

Table 7c. The structural comparison of KF-*ScBx3* with *TaBx3(A,B,D)*, *ZmBx3* and *HlBx3* genes
in exons/introns/3’UTR.

| **In KF-*ScBx3*** | *vs TaBx3A* | *vs TaBx3B* | *vs TaBx3D* | *vs ZmBx3* | *HlBx3* |
| --- | --- | --- | --- | --- | --- |
| The most/least frequent SNPs [%] | C/T; G/A (23,4)/  A/T; G/T (0,0) | A/G; C/T (18,3)/  T/G; C/A; G/T (1,7) | G/A (32,3)/  A/C; T/G (0,0) | T/C (19,6)/  A/T (1,9) | C/T (21,9)/  A/T (0,0) |
| The longest  insertion in exons/introns/3’UTR (bp) | none/1bp/1bp | none/4bp/none | none/1bp/none | 9bp/11bp/none | none/no data/none |
| The longest  deletion in exons/introns/3’UTR (bp) | none/48bp/24bp | none/56bp/42bp | none/40bp/24bp | 15bp/27bp/15bp | none/no data/32bp |
| The largest number of insertions in exons | none | none | none | 4 | none |
| The largest number of deletions in exons | none | none | none | 2 | none |
| The most polymorphic exon | 3 | 3 | 3 | 3 | 3 |
| The most frequent AA substitution type [%] | Val/Ile (13,3) | Glu/Asp (20,0) | Val/Ile (14,3)  Gln/Arg (14,3)  Ala/Glu (14,3) | Asp/Glu (4,0) | Val/Ile (12,0) |
| Type (frequency [%]) of non-conservative AA substitutions | Thr/Asn (6,7)  Ala/Glu (6,7)  Ala/Asp (6,7) | Ala/Glu (6,7)  Pro/Ala (6,7) | Ala/Glu (14,3)  Trp/Leu (7,1)  Ala/Asp (7,1) | Gln/Leu (1,6)  Ser/Trp (1,6)  Pro/Ser (1,6)  Thr/Gly (1,6)  Ala/Glu (0,8)  Ala/Pro (0,8)  Lys/Pro (0,8)  Glu/Thr (0,8)  Asn/Gly (0,8)  Pro/Arg (0,8)  Trp/Met (0,8)  Asp/Ala (0,8)  Phe/Ser (0,8)  Pro/Ala (0,8)  Glu/Gly (0,8)  Ile/Ala (0,8)  Pro/His (0,8)  Trp/Ser (0,8)  Thr/Arg (0,8)  Phe/Gln (0,8)  Gly/Gln (0,8)  Gln/Pro (0,8)  Trp/Gly (0,8)  Val/Lys (0,8)  Arp/Ala (0,8)  Gly/Pro (0,8)  Glu/Ala (0,8)  Ala/Gln (0,8)  Ser/Arg (0,8) | Gly/Val (4,0)  Ile/Gly (4,0)  Asp/Ala (4,0)  Pro/Leu (8,0)  Ile/Thr (4,0)  Ala/Glu (8,0)  Leu/Ser (4,0)  Trp/Leu (4,0) |

Table 7d. The structural comparison of KF-*ScBx4* with *TaBx4(A,B,D)*, *ZmBx4* and *HlBx4* genes
in exons/introns/3’UTR

| **In KF-*ScBx4*** | *vs TaBx4A* | *vs TaBx4B* | *vs TaBx4D* | *vs ZmBx4* | *HlBx4* |
| --- | --- | --- | --- | --- | --- |
| The most/least frequent SNPs [%] | T/C (19,1)/  A/T; T/A (0,0) | C/T (20,3)/  A/T; T/A (0,0) | G/A (21,9)/  A/T; A/C; T/A; T/G (1,4) | T/C (17,7)/  T/A; C/T (1,3) | A/G (23,5)/  A/T (0,0) |
| The longest  insertion in exons/introns/3’UTR (bp) | none/68bp/none | none/77bp/3bp | none/68bp/none | 9bp/47bp/none | none/no data/19bp |
| The longest  deletion in exons/introns/3’UTR (bp) | none/5bp/81bp | none/5bp/32bp | none/1bp/26bp | 15bp/none/166bp | none/no data/40bp |
| The largest number of insertions in exons | none | none | none | 5 | none |
| The largest number of deletions in exons | none | none | none | 2 | none |
| The most polymorphic exon (no.) | 3 | 1,3 | 3 | 2 | 3 |
| The most frequent AA substitution type [%] | Val/Thr (7,7)  Ala/Thr (7,7)  Thr/Ala (7,7)  Gly/Cys (7,7)  Gly/Ala (7,7)  Met/Thr (7,7)  His/Asn (7,7)  Gly/His (7,7)  Asn/Gly (7,7)  Arg/Gly (7,7)  Ile/Val (7,7)  Leu/Ser (7,7)  Ala/Glu (7,7) | V/al/Thr (12,5)  Ala/Thr (12,5)  Gly/Cys (12,5)  Gly/Ala (12,5)  Gly/His (12,5)  Asn/His (12,5)  Leu/Ser (12,5)  Ala/Glu (12,5) | Ala/Thr (15,4) | Ala/Ser (6,4) | Ala/Thr (19,1) |
| Type (frequency [%]) of non-conservative AA substitutions | Gly/Cys (7,7)  Met/Thr (7,7)  Gly/His (7,7)  Asn/Gly (7,7)  Arg/Gly (7,7)  Leu/Ser (7,7)  Ala/Glu (7,7) | Gly/Cys (12,5)  Gly/His (12,5)  Leu/Ser (12,5)  Ala/Glu (12,5) | Gly/Cys (7,7)  Arg/Cys (7,7)  Gly/His (7,7)  Leu/Ser (7,7)  Ala/Glu (7,7) | Arg/Val (0,9)  Gly/Val (0,9)  Gly/Cys (0,9)  Leu/Ala (1,8)  Thr/Gly (0,9)  Ile/Ala (0,9)  Trp/Ala (0,9)  Arg/Ala (0,9)  Ala/Arg (1,8)  Phe/Ser (0,9)  Ser/Pro (0,9)  Lys/Trp (0,9)  Gly/Lys (0,9)  Ala/His (0,9)  Asp/V (0,9)  Arg/Thr (0,9)  Met/Glu (0,9)  Ile/Thr (1,8)  Met/Lys (0,9)  Glu/Ala (0,9)  Gly/Leu (0,9)  Lys/Gly (0,9)  Leu/Thr (0,9)  His/Cys (0,9)  Ala/Glu (0,9)  Tyr/Pro (0,9) | Gly/Phe (4,8)  Asn/Gly (4,8)  Gly/Asn (4,8)  Glu/Gly (4,8)  Leu/Ser (4,8)  Ala/Glu (4,8) |

Table 7e. The structural comparison of KF-*ScBx5* with *TaBx5(A,B,D)*, *ZmBx5* and *HlBx5* genes
in exons/introns/3’UTR.

| **In KF-*ScBx5*** | *vs TaBx5A* | *vs TaBx5B* | *vs TaBx5D* | *vs ZmBx5* | *HlBx5* |
| --- | --- | --- | --- | --- | --- |
| The most/least frequent SNPs [%] | C/T (29,5)/  A/C; G/C (0,0) | C/T (23,9)/  A/T; G/T (2,2) | C/T (27,6)/  A/T; A/C (0,0) | C/G ((21,6)/  G/T (1,9) | C/T (27,2)/  A/T (0,0) |
| The longest  insertion in exons/introns/3’UTR (bp) | none/no data/915bp | none/no data/920bp | none/no data/961bp | 6 bp/182bp/124bp | none/no data/1006 bp |
| The longest  deletion in exons/introns/3’UTR (bp) | none/no data/none | 2 bp/no data/none | none/no data/none | 18 bp/none/44bp | 6 bp/no data/none |
| The largest number of insertions in exons | 1 | 1 | none | 4 | 1 |
| The largest number of deletions in exons | none | none | none | 2 | none |
| The most polymorphic exon | 1 | 1 | 1 | 3 | 3 |
| The most frequent AA substitution type [%] | K/R (21,4) | N/S (9,5) | L/V (18,2) | T/A (3,7) | D/E (13,3) |
| Type (frequency [%]) of non-conservative AA substitutions | T/M (7,1) | T/R (4,8);  S/M (4,8);  R/A (4,8) | none | L/Q (0,9)  T/Q (0,9)  A/L (0,9)  L/A (0,9)  L/T (0,9)  R/A (0,9)  R/S (0,9)  L/P (0,9)  R/P (0,9)  D/A (0,9)  L/C (0,9)  T/C (0,9)  A/N (0,9)  E/M (0,9)  N/Y (1,8)  Q/T (0,9)  A/D (1,8)  L/R (0,9)  Q/Y (0,9)  D/M (0,9)  Y/S (0,9)  T/R (0,9)  Q/W (0,9)  L/S (0,9)  T/G (0,9)  G/C (0,9)  K/L (0,9)  I/A (0,9)  V/S (0,9) | L/H (6,7)  L/C (6,7)  P/Q (6,7) |

Fig.1.

The predicted structure of BX proteins. a. BX2 proteins encoded by KF- and HG-*ScBx2* genes b. BX4 proteins encoded by KF- and HG-*ScBx4* genes; arrows indicate the unique α-helices present in the KF and HG BX proteins.

a)


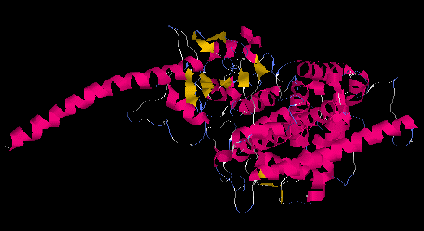

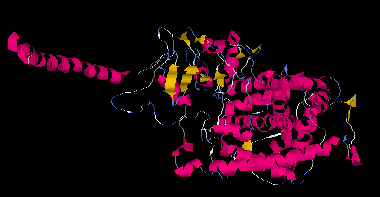


KF-*ScBx2* HG-*ScBx2*

b)


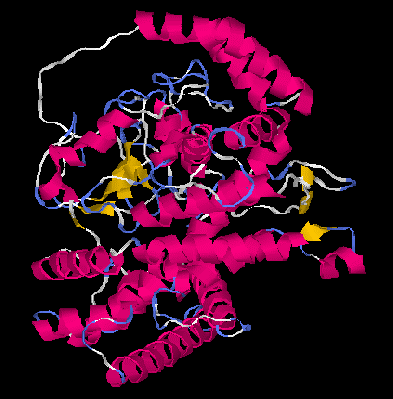

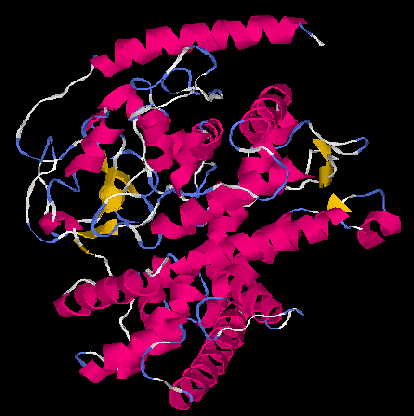


KF*-ScBx4* HG-*ScBx4*

Fig. 2.

Multiple sequence alignment for *Bx1* cds sequences from different *Poaceae* species.

1 50

ScBx1_L318_(KF636828) ATGGCTTTCG CGCTCAAT.. .......GCG TCTTGCTACC CTTCGTCATT

ScBx1_(JQ716987.1) atggctttcg cgctcaat.. .......gcg tcttgctacc cttcgtcatt

ScBx1_Picasso_(HG380515.1) ATGGCTTTCG CGCTCAAT.. .......GCG TCTTGCTACC CTTCGTCATT

TaBx1B_(AB124849.1) atggctttcg cgctcaat.. .......gcg tcttgctacc cttcgtcttt

TaBx1A_(AB094060.1) atggctttcg cgctcaat.. .......gcg tcttgctacc cttcgtcttt

TaBx1D_(AB124850.1) atggctttcg cgctcaat.. .......gca tcttgctacc cttcgtcttt

HlBx1_(AY462226.1) .......... .......... .......... .......... ..........

ZmBx1_(AY254103.1) ATGGCTTTCG CGCCCAAAAC GTCCTCCTCC TCCTCGCTGT CCTCGGCGTT

51 100

ScBx1_L318_(KF636828) CCAGTCGTCG .......... ..CTGCTCCC GAGGCGGATG GCAGCAGCGG

ScBx1_(JQ716987.1) ccagtcgtcg .......... ..ctgctccc gaggcggatg gcagcagcgg

ScBx1_Picasso_(HG380515.1) CCAGTCGTCG .......... ..CTGCTCCC GAGGCGGATG GCAGCAGCGG

TaBx1B_(AB124849.1) ccagtcgtcg .......... ..ctgctccc gaggcggatg gcagcagcgg

TaBx1A_(AB094060.1) ccagtcgtcg .......... ..ctgctccc gaggcggatg gcagcagcgg

TaBx1D_(AB124850.1) c...tcgtcg .......... ..ctgctccc gtggcggatg gcagcagcgg

HlBx1_(AY462226.1) .......... .......... .......... .......... ..........

ZmBx1_(AY254103.1) GCAGGCAGCT CAGTCGCCGC CGCTGCTCCT GAGGCGGATG TCGTCGACCG

101 150

ScBx1_L318_(KF636828) TGGTGATACC GGGGCGGAGG ...AATGTTC TGCCGGTCAT T.........

ScBx1_(JQ716987.1) tggtgatacc ggggcggagg ...aatgttc tgccggtcat t.........

ScBx1_Picasso_(HG380515.1) TGGTGATACC GGGGCGGAGG ...AATGTTC TGYCGGTCAT T.........

TaBx1B_(AB124849.1) tgatgatacc gaggcggagg ...aatgttc tgccggtcat t.........

TaBx1A_(AB094060.1) tgatgatacc gaggcggagg ...aatgttc tgcctgtcat t.........

TaBx1D_(AB124850.1) tgatgatacc aaggcggagg ...aatgttc tgcctgtcat t.........

HlBx1_(AY462226.1) .......... .......... .......... .......... ..........

ZmBx1_(AY254103.1) CAACA...CC GAGACGGAGG TACGACGCGG CCGTCGTCGT CACTACCACC

151 200

ScBx1_L318_(KF636828) .........A GG........ ....GCGGTT GCAGTG.... ..GCTCCGAC

ScBx1_(JQ716987.1) .........a gg........ ....gcggtt gcagtg.... ..gctccgac

ScBx1_Picasso_(HG380515.1) .........A GG........ ....GCGGTT GCAGTG.... ..GCTCCGAC

TaBx1B_(AB124849.1) .........a gg........ ....gcggtc gcggtg.... ..gctccgcc

TaBx1A_(AB094060.1) .........a gg........ ....gcggtt gccgtg.... ..gctccgcc

TaBx1D_(AB124850.1) .........a ag........ ....gcggtt gcggtg.... ..gctccgcc

HlBx1_(AY462226.1) .......... .......... .......ggc accagg.... ..gcgccagt

ZmBx1_(AY254103.1) ACCACTGCTA GAGCTGCGGC GGCTGCTGTC ACGGTTCCCG CCGCCCCGCC

201 250

ScBx1_L318_(KF636828) T...GCCCCG GCGCCGGCT. .......... .AAG...... ......CCAG

ScBx1_(JQ716987.1) t...gccccg gcgccggct. .......... .aag...... ......ccag

ScBx1_Picasso_(HG380515.1) T...GCCCCG GCGCCGGCT. .......... .AAG...... ......CCAG

TaBx1B_(AB124849.1) c...gccctg gcgccggct. .......... .aag...... ......ccag

TaBx1A_(AB094060.1) c...gccccg gcgccggcg. .......... .aag...... ......ccag

TaBx1D_(AB124850.1) c...gccccg gcgccggcg. .......... .aag...... ......ccag

HlBx1_(AY462226.1) g......ccc gtgcctgtc. .......... .......... ......gccg

ZmBx1_(AY254103.1) GCAGGCGCCG GCGCCGGCGC CGGTGCCACC AAAGCAAGCG GCGGCACCCG

251 300

ScBx1_L318_(KF636828) CAGCTGTGAG GAGCCGGACC GTGTCGGACA CCATGGCCAA GCTCATGGCT

ScBx1_(JQ716987.1) cagctgtgag gagccggacc gtgtcggaca ccatggccaa gctcatggct

ScBx1_Picasso_(HG380515.1) CAGCTGTGAG GAGCCGGACC GTGTCGGACA CCATGGCCAA GCTCATGGCT

TaBx1B_(AB124849.1) cagctgtgag gggccggacc gtgtcggaca ccatggccaa gctcatggct

TaBx1A_(AB094060.1) cagccgtgag gagccggccc gtgtcggtca ccatggccaa gctcatggct

TaBx1D_(AB124850.1) cagccgtgag gagccggccc gtgtcggtca ccatggccaa gctcatggct

HlBx1_(AY462226.1) ccggcgatcg cggcctgtcc gtttcgcagg ccatgtccaa agtcatggag

ZmBx1_(AY254103.1) CCGAGAGGAG GAGCCGTCCG GTGTCGGACA CCATGGCGGC GCTCATGGCC

301 350

ScBx1_L318_(KF636828) AAGGGCAAGA CAGCGCTCAT CCCGTATATC ACCGCCGGTG ATCCTGACCT

ScBx1_(JQ716987.1) aagggcaaga cagcgctcat cccgtatatc accgccggtg atcctgacct

ScBx1_Picasso_(HG380515.1) AAGGGCAAGA CAGCGCTCAT CCCGTATATC ACCGCCGGTG ATCCTGACCT

TaBx1B_(AB124849.1) aagggaaaga cagcgctcat cccgtatatc accgccggtg atcctgacct

TaBx1A_(AB094060.1) aagggcaaga cagcgctcat cccgtatatc accgccggtg atcctgacct

TaBx1D_(AB124850.1) aagggcaaga cagcgctcat cccgtatatc accgccggtg atcctgacct

HlBx1_(AY462226.1) aagggtaaga cggcgttcat cccgtacatc acggccggcg accccgacct

ZmBx1_(AY254103.1) AAGGGCAAGA CGGCGTTCAT CCCGTACATC ACCGCCGGCG ACCCGGACCT

351 400

ScBx1_L318_(KF636828) CGCCACAACG GCGGAGGCGC TGCGTCTCCT GGATGCCTGT GGCGCCGACG

ScBx1_(JQ716987.1) cgccacaacg gcggaggcgc tgcgtctcct ggatgcctgt ggcgccgacg

ScBx1_Picasso_(HG380515.1) CGCCACAACG GCGGAGGCGC TGCGTCTCCT GGATGCCTGT GGCGCCGACG

TaBx1B_(AB124849.1) ggccacaacg gcggaggcgc tgcgtctcct ggatgcctgt ggcgccgacg

TaBx1A_(AB094060.1) ggccacaacg gcagaggcgc tgcatctcct ggatgcctgt ggcgccgacg

TaBx1D_(AB124850.1) ggccacaacg gcggaggcgc tgcgtctcct ggatgcgtgt ggcgccgacg

HlBx1_(AY462226.1) ggcaacgacg gcggcggcgc tgaggctcct cgacgccctg ggcgcggacg

ZmBx1_(AY254103.1) AGCGACGACG GCCGAGGCGC TGCGTCTGCT GGACGGCTGT GGCGCCGACG

401 450

ScBx1_L318_(KF636828) TCATTGAGCT CGGCGTGCCC TGCTCCGACC CCTACGTGGA CGGTCCGATC

ScBx1_(JQ716987.1) tcattgagct cggcgtgccc tgctccgacc cctacgtgga cggtccgatc

ScBx1_Picasso_(HG380515.1) TCATTGAGCT CGGCGTGCCC TGCTCCGACC CCTACGTGGA CGGTCCGATC

TaBx1B_(AB124849.1) tcattgagct cggcgtgcca tgctccgacc cctacgtgga cggtccgatc

TaBx1A_(AB094060.1) tcattgagct cggcgtgccg tgctccgacc cctacgtgga cggtccgatc

TaBx1D_(AB124850.1) tcattgagct tggcgtgcca tgctccgacc cctacgtgga cggtccgatc

HlBx1_(AY462226.1) tcgtcgagct cggcatgccg ttctcggatg cctcggccga cggggccgtc

ZmBx1_(AY254103.1) TCATCGAGCT GGGGGTACCC TGCTCGGACC CCTACATCGA CGGGCCCATC

451 500

ScBx1_L318_(KF636828) ATCCAGGCTT CGAGTGCGAG GGCTCTAGCG AGCGGTGCGA CAATGGACGG

ScBx1_(JQ716987.1) atccaggctt cgagtgcgag ggctctagcg agcggtgcga caatggacgg

ScBx1_Picasso_(HG380515.1) ATCCAGGCTT CGAGTGCGAG GGCTCTAGCG AGCGGTGCGA CAATGGACGG

TaBx1B_(AB124849.1) atccaggctt caagtgcgag ggctctggcg ggcggtgcga caatggacgg

TaBx1A_(AB094060.1) atccaggctt cgagtgcgag ggctctcgct ggcggtgcca caatggacgg

TaBx1D_(AB124850.1) atccaggctt cgagtgcgag ggctctggcg ggcggtgcca caatggacgg

HlBx1_(AY462226.1) atcaaggcct ccgcggcgcg cgcgctggcc gctggcgcga cggttgacgc

ZmBx1_(AY254103.1) ATCCAGGCGT CGGTGGCGCG GGCTCTGGCC AGCGGCACCA CCATGGACGC

501 550

ScBx1_L318_(KF636828) CGTGCTGGCG ATGCTCAAGG AGGTGACGCC GGAGCTGTCG TGCCCGGTGG

ScBx1_(JQ716987.1) cgtgctggcg atgctcaagg aggtgacgcc ggagctgtcg tgcccggtgg

ScBx1_Picasso_(HG380515.1) CGTGCTGGCG ATGCTCAAGG AGGTGACGCC GGAGCTGTCG TGCCCGGTGG

TaBx1B_(AB124849.1) cgtgctggcg atgctcaagg aggtgacgcc ggagctgtcg tgcccggtgg

TaBx1A_(AB094060.1) cgtgctggcg atgctcaagg aggtgacgcc ggagctgtcg tgccctgtgg

TaBx1D_(AB124850.1) cgtgctggcg atgctcaagg aggtgacgcc ggagctgtcg tgccctgtgg

HlBx1_(AY462226.1) catcatggcg atgctgaagg aggtgacgcc ggagctttcc tgccccgtgg

ZmBx1_(AY254103.1) CGTGCTGGAG ATGCTGAGGG AGGTGACGCC GGAGCTGTCG TGCCCCGTGG

551 600

ScBx1_L318_(KF636828) TGCTCTTCTC GTACTACAGG CCCATCTTGT GTCGAGGG.. ..........

ScBx1_(JQ716987.1) tgctcttctc gtactacagg cccatcttgt gtcgaggg.. ..........

ScBx1_Picasso_(HG380515.1) TGCTCTTCTC GTACTACAGG CCCATCTTGT GTCGAGGG.. ..........

TaBx1B_(AB124849.1) tgctcttctc gtattacagg cctatcttgt gtcgaggg.. ..........

TaBx1A_(AB094060.1) tgctcttctc gtactacagg cctatcttat gtcgaggg.. ..........

TaBx1D_(AB124850.1) tgctcttctc gtactacagg cctatcttgt gtcgaggg.. ..........

HlBx1_(AY462226.1) tgatcttctc ctacttcagc cccattgcgc agcgagggac ggcgagcttc

ZmBx1_(AY254103.1) TGCTCCTCTC CTACTACAAG CCCATCATGT CTCGCAGC.. ..........

601 650

ScBx1_L318_(KF636828) TTGGCCGAAA TCAAAGAAGC CGGTGTACAC GGTCTTATAG TGCCTGATCT

ScBx1_(JQ716987.1) ttggccgaaa tcaaagaagc cggtgtacac ggtcttatag tgcctgatct

ScBx1_Picasso_(HG380515.1) TTGGCCGAAA TCAAAGAAGC CGGTGTACAC GGTCTTATAG TGCCTGATCT

TaBx1B_(AB124849.1) ttggccgaaa tcaaagaagc cggtgtgcac ggtcttatag tgcctgatct

TaBx1A_(AB094060.1) ttggccgaaa tcaaagaagc cggtgtacac ggtcttatag tgcctgatct

TaBx1D_(AB124850.1) ttggccgaaa tcaaagaagc cggtgtacac ggtcttatag tgcctgatct

HlBx1_(AY462226.1) gccgcagcgg tcaaggaagc cggcgtgaaa ggtcttatag tacccgatct

ZmBx1_(AY254103.1) TTGGCCGAGA TGAAAGAGGC GGGGGTCCAC GGTCTTATAG TGCCTGATCT

651 700

ScBx1_L318_(KF636828) CCCTTATGTA GCCGCGCATT CATTATGGAG TGAAGCCAAG AAGAACAACC

ScBx1_(JQ716987.1) cccttatgta gccgcgcatt cattatggag tgaagccaag aagaacaacc

ScBx1_Picasso_(HG380515.1) CCCTTATGTA GCCGCGCATT CATTATGGAG TGAAGCCAAG AAGAACAACC

TaBx1B_(AB124849.1) cccttatgta gccgcgcatg cattatggag tgaagccaag aagaacaacc

TaBx1A_(AB094060.1) cccttatgta gccgcgcatg cattatggag tgaagccaag aagaacaacc

TaBx1D_(AB124850.1) cccttatgta gccgcgcatg cattatggag tgaagccaag aagaacaacc

HlBx1_(AY462226.1) tccttacgcc gagacaagtg ctttcaggga tgaagccatc aaaaacgagc

ZmBx1_(AY254103.1) CCCGTACGTG GCCGCGCACT CGCTGTGGAG TGAAGCCAAG AACAACAACC

701 750

ScBx1_L318_(KF636828) TCGAGCTGGT GCTGCTCACA ACACCAGCCA TACCAGAAGA AAGAATGAAA

ScBx1_(JQ716987.1) tcgagctggt gctgctcaca acaccagcca taccagaaga aagaatgaag

ScBx1_Picasso_(HG380515.1) TCGAGCTGGT GCTGCTCACA ACACCAGCCA TACCAGAAGA AAGAATGAAA

TaBx1B_(AB124849.1) tcgagctggt gctgctcaca acaccagcca taccagaaga aagaatgaag

TaBx1A_(AB094060.1) tcgagctggt gctgctcaca acaccagcca taccagaaga aagaatgaag

TaBx1D_(AB124850.1) tcgagctggt gctgctcaca acaccagcca taccagaaga aagaatgaag

HlBx1_(AY462226.1) tagagctggt tttacttaca acaccatcta caccgccaga gaggatgaag

ZmBx1_(AY254103.1) TGGAGCTGGT GCTGCTGACA ACACCAGCCA TACCAGAAGA CAGGATGAAG

751 800

ScBx1_L318_(KF636828) GAAATCACGA AAGCTTCGGA AGGTTTCATT TACCTGGTGA GCGTCAATGG

ScBx1_(JQ716987.1) gaaatcacga aagcttcgga aggtttcatt tacctggtga gcgtcaatgg

ScBx1_Picasso_(HG380515.1) GAAATCACGA AAGCTTCGGA AGGTTTCATT TACCTGGTGA GCGTCAATGG

TaBx1B_(AB124849.1) gaaatcacga aagcttcaga aggtttcatt tacctggtga gcgtcaatgg

TaBx1A_(AB094060.1) ggaatcacga aagcttcgga aggtttcatt tacctggtga gcgtcaatgg

TaBx1D_(AB124850.1) gaaatcacga aagcttcgga aggtttcatt tacctggtga ccgtcaatgg

HlBx1_(AY462226.1) gagatcactg aagcttcagg aggcttcgtt taccttgtaa gtgtcgacgg

ZmBx1_(AY254103.1) GAGATCACCA AGGCTTCAGA AGGCTTCGTC TACCTGGTGA GCGTCAACGG

801 850

ScBx1_L318_(KF636828) AGTTACAGGT CCCCGTGAAA ACGTGAACCT GCGGGTTGAG TCCCTCATTC

ScBx1_(JQ716987.1) agttacaggt ccccgtgaaa acgtgaacct gcgggttgag tccctcattc

ScBx1_Picasso_(HG380515.1) AGTTACAGGT CCCCGTGAAA ACGTGAACCT GCGGGTTGAG TCCCTCATTC

TaBx1B_(AB124849.1) agttacaggt ccccgtgaaa acgtgaatct gcgggttgag tccctcattc

TaBx1A_(AB094060.1) agttacaggt ccccgtgaaa acgtgaacct gcgggttgag tccctcattc

TaBx1D_(AB124850.1) agttacaggt ccccgtgaaa acgtgaacct tcgggttgag tccctcattc

HlBx1_(AY462226.1) agttaggggt gcccgtgcaa ccgtgaaccc acgcgtcgag agtcttctta

ZmBx1_(AY254103.1) AGTGACAGGT CCTCGCGCAA ACGTGAACCC ACGAGTGGAG TCACTCATCC

851 900

ScBx1_L318_(KF636828) AAGAGATAAA AAAGGTTACT GACAAACCTG TTGCTGTTGG CTTTGGCATA

ScBx1_(JQ716987.1) aagagataaa aaaggttact gacaaacctg ttgctgttgg ctttggcata

ScBx1_Picasso_(HG380515.1) AAGAGATAAA AAAGGTTACT GACAAACCTG TTGCTGTTGG CTTTGGCATA

TaBx1B_(AB124849.1) aagagataaa aaaggttact gacaaacctg ttgctgttgg ctttggcata

TaBx1A_(AB094060.1) aagagataaa aaaggttact gacaaacctg ttgctgttgg ctttggcata

TaBx1D_(AB124850.1) aagagataaa aaaggttact gacaaacctg ttgctgttgg ctttggcata

HlBx1_(AY462226.1) agaagattaa gcaggtcact gacaaggcag tggctgttgg ctttggaatt

ZmBx1_(AY254103.1) AGGAGGTTAA GAAGGTGACT AACAAGCCCG TTGCTGTTGG CTTCGGCATA

901 950

ScBx1_L318_(KF636828) TCAAAACCTG AACACGTGAA GCAGATTGCA GGGTGGGGTG CGGATGGGGT

ScBx1_(JQ716987.1) tcaaaacctg aacacgtgaa gcagattgca ggatggggtg cggatggggt

ScBx1_Picasso_(HG380515.1) TCAAAACCTG AACACGTGAA GCAGATTGCA GGGTGGGGTG CGGATGGGGT

TaBx1B_(AB124849.1) tcaaaacctg aacacgtaaa gcagattgca gggtggggtg cggatggggt

TaBx1A_(AB094060.1) tcaaaacctg aacacgtaaa gcagattgca ggatggggtg cggatggggt

TaBx1D_(AB124850.1) tcaaaacctg aacacgtaaa gcagattgca agatggggtg cggatggggt

HlBx1_(AY462226.1) tcgacccctg accatgttaa gcagattgca gagtggggtg cagatggagt

ZmBx1_(AY254103.1) TCCAAGCCCG AGCACGTAAA GCAGATTGCG CAGTGGGGCG CTGACGGGGT

951 1000

ScBx1_L318_(KF636828) GATCATTGGC AGTGCGATGG TGAGGCAGTT AGGTGAAGCA GCTTCGCCCA

ScBx1_(JQ716987.1) gatcattggc agtgcgatgg tgaggcagtt aggtgaagca gcttcgccca

ScBx1_Picasso_(HG380515.1) GATCATTGGC AGTGCGATGG TGAGGCAGTT AGGTGAAGCA GCTTCGCCCA

TaBx1B_(AB124849.1) gatcattggc agtgcgatgg tgaggcagtt aggtgaagca gcttcgccca

TaBx1A_(AB094060.1) gatcattggc agtgcgatgg tgaggcagtt gggtgaagca gcttcgccca

TaBx1D_(AB124850.1) gatcattggc agtgcgatgg tgaggcagtt gggtgaagca gcttcgccca

HlBx1_(AY462226.1) gatcatcggc agtgcaatgg tgaagcagtt aggtgaagca gcttctccag

ZmBx1_(AY254103.1) GATCATCGGC AGCGCCATGG TGAGGCAGCT GGGCGAAGCG GCTTCTCCCA

1001 1050

ScBx1_L318_(KF636828) AAGAAGGATT GAAAAGGCTA GAGGCATATG CCAGGAGCAT GAAGAATGCA

ScBx1_(JQ716987.1) aagaaggatt gaaaaggcta gaggcatatg ccaggagcat gaagaatgca

ScBx1_Picasso_(HG380515.1) AAGAAGGATT GAAAAGGCTA GAGGCATATG CCAGGAGCAT GAAGAATGCA

TaBx1B_(AB124849.1) aagaaggatt gaaaagacta gaggcatatg ccaggagcat gaagaatgca

TaBx1A_(AB094060.1) gagaaggatt gaaaaggcta gaggcatatg cgaggagcat gaagaatgca

TaBx1D_(AB124850.1) gagaaggatt gaaaaggcta gaggcatatg ccaggagcat gaagaatgca

HlBx1_(AY462226.1) aagaagggtt gaaaaggttg gaagtctatg ccaggagctt gaaggatgca

ZmBx1_(AY254103.1) AGCAAGGCCT GAGGAGGCTG GAGGAGTATG CCAGGGGCAT GAAGAACGCG

1051 1059

ScBx1_L318_(KF636828) CTACCATGA

ScBx1_(JQ716987.1) ctaccatga

ScBx1_Picasso_(HG380515.1) CTACCATGA

TaBx1B_(AB124849.1) ctaccatga

TaBx1A_(AB094060.1) ctaccatga

TaBx1D_(AB124850.1) ctaccatga

HlBx1_(AY462226.1) ctgccatga

ZmBx1_(AY254103.1) CTGCCATGA

Fig.3.

Multiple sequence alignment for *Bx2 – Bx5* cds sequences from different *Poaceae* species

1 50

ScBx2_L318_(KF620524) .......... .......... .......... ...ATGCTCC ACGAAGCAGC

ScBx2_(JX442061.1) .......... .......... .......... ...atgctcc acgaagcagc

ScBx2_Picasso_(HG380516.1) ATGGCT...C AGGTACAT.. ....GTAGAA GAGATGCTCC ACGAAGCAGC

ZmBx2_(X81831.1) atggctgctc aactgcatca cgcactatac gagctgctgc acgaagcagc

HlBx2_(AY462227.1) atggct...c acgtacat.. ....gtagac gagatgctcc ac...ggagc

TaBx2A_(AB042630.1) atggct...c acgtacat.. ....gtagac gagctgctcc acgaagcagc

TaBx2B_(AB042631.1) atggct...c acgtacat.. ....gtagac gagatgctcc acgaagcagc

TaBx2D_(AB124851.1) atggct...c acgtacat.. ....gtagac gagatgctcc acgaagcagc

ScBx3_(KF636827) ATGGCT.... ..CTTGAAGC AGCATACCAC ...TACCTGC AGCTCGCCGG

ScBx3_Picasso_(HG380517.1) ATGGCT.... ..CTTGAAGC AGCATACCAC ...TACCTGC AGCTCGCCGG

TaBx3B_(AB124853.1) atggct.... ..cttgaagc agccttccac ...tacctgc agctcgccgg

TaBx3D_(AB124852.1) atggct.... ..cttgaagc agcgttccac ...tacctgc agctcgccgg

TaBx3A_(AB042628.1) atggct.... ..cttgaagc agcgaaccac ...tacctgc agctcgccgg

ZmBx3_(X81829.1) atggcc.... ..cttggagc tgcgtaccat cactacctgc agctcgccgg

HlBx3_(AY462228.1) atggct.... ..cttgaagc agcataccac ...tacctgc agatcgccgt

ScBx4_(KF636826) ATGGCT.... ..CTTGAAGC AGCGTACCAC ...TACCTGC AGCGCGCCGT

ScBx4_Picasso_(HG380518.1) ATGGCT.... ..CTTGAAGC AGCGTACCAC ...TAC.... ..........

HlBx4_(AY462229.1) atggct.... ..cttgaagc agcgtaccac ...tacctgc agcgcgccgt

TaBx4A_(AB124854.1) atggct.... ..cttgaagc agcgtaccac ...tacctgc agcgcgccgt

TaBx4D_(AB042627.1) atggct.... ..cttgaagc agcgtaccac ...tacctgc agcgcgccgt

TaBx4B_(AB124855.1) atggct.... ..cttgaagc agcgtaccac ...tacctgc agcgcgctgt

ZmBx4_(X81827.1) atggct.... ..ctcgaagc agcgtacgac ...tacctgc acgtcgccgt

ScBx5_(KF636825) ATGGCT.... ..CTTGAAGC TGCCCACCAC ...TACCTGC AGCTCGCCGT

TaBx5D_(AB124857.1) atggct.... ..cttgaagc tgcccaccac ...tacctgc agctcgccgt

TaBx5A_(AB042629.1) atggct.... ..cttgaagc agcccagcac ...tacctgc agctcgccgt

HlBx5_(AY462230.1) atggct.... ..cttgaagc tgcccaccac ...tacctgc ggcacgccgt

TaBx5B_(AB124856.1) atggct.... ..ttcgaagg agcataccat ...ttcctgc agctcgccgt

ZmBx5_(X81830.1) ...gca.... ..ctccaggc agcctacgag ...tacctgc agcaggccgt

ScBx5_Picasso_(HG380519.1) ATGGCT.... ..CTTGAAGC TGCCCACCAC ...TACCTGC AGCTCGCCGT

51 100

ScBx2_L318_(KF620524) GGCGGCCGCT CCACGATCTC TCTTGGTTGC CACTGCGGTG CTCTTCTCCC

ScBx2_(JX442061.1) agcggccgct ccacgatctc tcttggttgc cactgcggtg ctcttctccc

ScBx2_Picasso_(HG380516.1) GGCGGCCGCT CCACGATCTC TCTTGGTTGC CACTTCGGTG CTCTTCTCCC

ZmBx2_(X81831.1) agcagcgcaa ...cgagctc tgctgctcgc g......atc cccttctccc

HlBx2_(AY462227.1) agcagcagct ccgcgatctc tcctgattgc gactgcggtg ctcttctccc

TaBx2A_(AB042630.1) agcggcagct ccacgatctc tcttgatcgc gagtgcggtg ctcttctccc

TaBx2B_(AB042631.1) agcagccgct ccacgatctc tcttgattgc gactgcggtg ctcttctccc

TaBx2D_(AB124851.1) agcggcagct ccacgatctc tcttgattgc gactgcggtg ctcttctccc

ScBx3_(KF636827) CATCCATGGC ......ACAT CCACGCCGGC G......GTA CTACTCACC.

ScBx3_Picasso_(HG380517.1) CATCCATGGC ......ACAT CCACGCCGGC G......GTA CTACTCACC.

TaBx3B_(AB124853.1) cgtccatggc ......acat ccacgccggc a......cta ctgctcacc.

TaBx3D_(AB124852.1) catccatggc ......acat ccacgccggc a......gta ctactcacc.

TaBx3A_(AB042628.1) cgtccatggc ......acat ccacgccggc a......gta ctactcacc.

ZmBx3_(X81829.1) cgaccatggc ......accg ccacacacgc a......ctg ctcctcggc.

HlBx3_(AY462228.1) cggccatggc ......acat ccacgccggc g......gca ctactcacc.

ScBx4_(KF636826) CGGCCATGGC ......GTAT CCACAGAAGC A...CTA... CTGCTCACC.

ScBx4_Picasso_(HG380518.1) .......... .......... .......... .......... ..........

HlBx4_(AY462229.1) cggccatggc ......acaa ccacagaggc a...cta... ctgctcacc.

TaBx4A_(AB124854.1) cggccatggc ......acat ccacagaagc a...cta... ctactcacc.

TaBx4D_(AB042627.1) cggccatggc ......acat ccacagaagc a...cta... ctactcacc.

TaBx4B_(AB124855.1) cggccatggc ......acat ccacagaagc a...cta... ctactcacc.

ZmBx4_(X81827.1) cgtccagtgc ......acgc ccacacaagc a...gcagca gtcctcggc.

ScBx5_(KF636825) CGGCCATGGC ......ACGT CCACGCCGGC G...GCACTG CTCCTCGTC.

TaBx5D_(AB124857.1) cggccatggc ......acat ccacgccggc g...gtactc ctcatcgtc.

TaBx5A_(AB042629.1) cggccatggc ......atgt cctcgccggc g...gcactg ctccttgtc.

HlBx5_(AY462230.1) cggccatggc ......acgt ccgcgccagc g...gcactg ctcctcgtc.

TaBx5B_(AB124856.1) cggccatgcc ......acat cctcgccggc g...gcactg ctcctcgtc.

ZmBx5_(X81830.1) cggccatggc gcgtggtcgt ccacgcagac gctgacgctg ctgctcatc.

ScBx5_Picasso_(HG380519.1) CGGCCATGGC ......ACGT CCACGCCGGC G...GCACTG CTCCTCGTC.

101 150

ScBx2_L318_(KF620524) TTGTGGTTCT GCCGCTCCTA CTCCGCATTA TT........ ..........

ScBx2_(JX442061.1) ttgtggttct gccgctccta ctccgcatta tt........ ..........

ScBx2_Picasso_(HG380516.1) TTGTGGTTCT GCCGCTCCTA CTCCGCATTA TT........ ..........

ZmBx2_(X81831.1) tgctgctcct gccgctcctg ctccgctacc tcgcagcgtc ggcgtcggcg

HlBx2_(AY462227.1) ttgtggttct gccgctccta ctccgcatta tt........ ..........

TaBx2A_(AB042630.1) ttgtggttgt gccgctccta ctccgcatta tt........ ..........

TaBx2B_(AB042631.1) ttgtggttct gccgctccta ctccgcatta tt........ ..........

TaBx2D_(AB124851.1) ttgtggttgt gccgctcctg ctccgcatta tt........ ..........

ScBx3_(KF636827) ..GTTCTCCT GCTACTCATC GTTCGACTA. .......... .......GCA

ScBx3_Picasso_(HG380517.1) ..GTTCTCCT GCTACTCATC GTTCGACTA. .......... .......GCA

TaBx3B_(AB124853.1) ..gttctcct gctactcatc attcgacta. .......... .......gca

TaBx3D_(AB124852.1) ..attctcct gctactcatc attcgacta. .......... .......gca

TaBx3A_(AB042628.1) ..gttctcct gctactcatc attcgacta. .......... .......gca

ZmBx3_(X81829.1) ..gtacttat cttcctggtc atccgccta. .......... .......gta

HlBx3_(AY462228.1) ..gttctcct gctactcatc attcgacta. .......... .......gca

ScBx4_(KF636826) ..GTTCTCCT GCTACTCATC ATCCGACTAG CATGG..... .......GTA

ScBx4_Picasso_(HG380518.1) ........CT GCTACTCATC ATCCGACTAG CATGG..... .......GTA

HlBx4_(AY462229.1) ..gttctcct gctactcatc atccgactag catgg..... .......gta

TaBx4A_(AB124854.1) ..gttctcct gctactcatc atccgactag catgg..... .......gta

TaBx4D_(AB042627.1) ..gttctcct gctactcatc atccgagtag catgg..... .......gta

TaBx4B_(AB124855.1) ..gttctcct gctactcatc atccgactag catgg..... .......gta

ZmBx4_(X81827.1) ..gtcctcct cctcctcgcc atccggctag cc........ .......gcg

ScBx5_(KF636825) ..CTCGTGCC CTTACTGCTG TTACTGTTA. .......... .......GCA

TaBx5D_(AB124857.1) ..gttgttcc cttactgatg ttagtgcta. .......... .......gta

TaBx5A_(AB042629.1) ..gttgttcc cttactgctg ttactgcta. .......... .......gca

HlBx5_(AY462230.1) ..tgcgtacc attactgctg ttgctgttgc tgttc..... .......gca

TaBx5B_(AB124856.1) ..gtagtacc cttactgctg ttactgcta. .......... .......gca

ZmBx5_(X81830.1) ..gccgtacc caccgtacta ctgctgcta. .......... .......gcg

ScBx5_Picasso_(HG380519.1) ..CTCGTGCC CTTACTGCTG TTACTGTTA. .......... .......GCA

151 200

ScBx2_L318_(KF620524) ......ACCA AGCAG...GG AGCGGCA... ...AGCGATG CC...AAGCT

ScBx2_(JX442061.1) ......acca agcag...gg agcggca... ...agcgatg cc...aagct

ScBx2_Picasso_(HG380516.1) ......ACCA AGCAG...GG AGCGGCA... ...AGCGATG CC...AAGCT

ZmBx2_(X81831.1) tcggcgacga agaacgacgg agcggcgccg gccagcgacc ccgacaagct

HlBx2_(AY462227.1) ......acca agcag...gg agcggcg... ...agcgatg cc...aagct

TaBx2A_(AB042630.1) ......agca agcag...gg agcggca... ...agcgatg cc...aagct

TaBx2B_(AB042631.1) ......gcca agcag...gg agcggca... ...agcgatg cc...aagct

TaBx2D_(AB124851.1) ......acca agcag...gg agcggca... ...agcgacg cc...aagct

ScBx3_(KF636827) TGGGTAAGGA CCACA...AC CGCGTCGACA AGATTCGGCA AG...CAGCA

ScBx3_Picasso_(HG380517.1) TGGGTAAGGA CCACA...AC CGCGTCGACA AGATTCGGCA AG...CAGCA

TaBx3B_(AB124853.1) tgggtgagga ccaca...ac ggtgtcaaca agattcggca ag...cagca

TaBx3D_(AB124852.1) tgggtgagga ccaca...ac tgcgtcaaca agattcggta ag...cagca

TaBx3A_(AB042628.1) tgggtgagga ccgca...ac cgcgtcaaca agattcggca ag...cagca

ZmBx3_(X81829.1) tccgcaaggc gaaccggcac aacctcagcc aacaaacgta ag...cagca

HlBx3_(AY462228.1) tgggtaagga ccaca...ac cgcgtcaaca agattgagca ag...cagca

ScBx4_(KF636826) AGAGCCTTCG CCACC..... .ACCGCCGCG TCAACAAAAG GC...AAGCA

ScBx4_Picasso_(HG380518.1) AGAGCCTTCG CCACC..... .ACCGCCGCG TCAACAAAAG GC...AAGCA

HlBx4_(AY462229.1) agagccttca ccagc..... .accaccaca tcaacaaaat tc...aagca

TaBx4A_(AB124854.1) agagccttcg ccacc..... .accaccgca tcagcaaaat gc...aagca

TaBx4D_(AB042627.1) agagccttca ccacc..... .accaccgca tcgacaaaat gc...aagca

TaBx4B_(AB124855.1) agagccttcg ccacc..... .accaccgca tcaacaaaat gc...aagca

ZmBx4_(X81827.1) gcggcgagga gctcg..... .tccgcgacg tcccccaaat gg...aagca

ScBx5_(KF636825) TCTGTAAGGA CCTCA..... .GCATCCACA ......AGA. .....AAGCT

TaBx5D_(AB124857.1) tcagtaagaa catca..... .gcgtccaca ......aga. .....aagct

TaBx5A_(AB042629.1) tcagtaagaa cctca..... .gcgtccaca ......aga. .....aagct

HlBx5_(AY462230.1) tcgctaagaa cctca..... .gcgtcgaca ......aga. .....aagct

TaBx5B_(AB124856.1) tcggtaagga ggtcc..... .accatgacc gga...cgc. .....aagct

ZmBx5_(X81830.1) tccctcgcca agagc..... .acgtcgtcg tccggtaggg gc...aagcc

ScBx5_Picasso_(HG380519.1) TCTGTAAGGA CCTCA..... .GCATCCACA ......AGA. .....AAGCT

201 250

ScBx2_L318_(KF620524) GCTGAGC... ...CTGCTCC CATCTCCCCC GACGAAGCTC CCCATCATCG

ScBx2_(JX442061.1) gctgagc... ...ctgctcc catctccccc gacgaagctc cccatcatcg

ScBx2_Picasso_(HG380516.1) GCTGAGC... ...CTGCTCC CATCTCCCCC GACGAAGCTC CCCATCATCG

ZmBx2_(X81831.1) cttgagc... ...ctgctgc cgtcgccgcc catgaagctc cccatcatcg

HlBx2_(AY462227.1) gctgagc... ...ctgctcc cgtctccccc gagcaagctc cccatcatcg

TaBx2A_(AB042630.1) gctgagc... ...ctgctcc catctccccc gacgaagctc cccatcatcg

TaBx2B_(AB042631.1) gctgagc... ...ctgctcc catctccccc gacgaggctc cccatcatcg

TaBx2D_(AB124851.1) gctgagc... ...ctgctcc catctccccc gacgaggctc cccataatcg

ScBx3_(KF636827) GCAG...... ...CTCCCCC CTTCACCTCC AGGCAAGCTG CCCATCATCG

ScBx3_Picasso_(HG380517.1) GCAG...... ...CTCCCCC CTTCACCTCC AGGCAAGCTG CCCATCATCG

TaBx3B_(AB124853.1) gcag...... ...ctcccgc cttcacctcc aggcaagctg cccatcatcg

TaBx3D_(AB124852.1) acag...... ...ctcccgc cttcaccccc aggcaagctg cccatcatcg

TaBx3A_(AB042628.1) acag...... ...ctcccgc cttcacctcc aggcaagctg cccatcatcg

ZmBx3_(X81829.1) acagcggctt ccgctcccgc cttggccccc gggcaagctg cccatcatcg

HlBx3_(AY462228.1) gcag...... ...ctcccgc cttcacctcc aggcaagctg cccatcatcg

ScBx4_(KF636826) GCAG...... ...CTCCCGC CTACACCTCC AGGCAAGCTG CCCATCATCG

ScBx4_Picasso_(HG380518.1) GCAG...... ...CTCCCGC CTACACCTCC AGGCAAGCTG CCCATCATCG

HlBx4_(AY462229.1) gcag...... ...ctcccgc ctacacctcc aggcaagctg cccatcatcg

TaBx4A_(AB124854.1) gcag...... ...ctcccgc ctacacctcc cggcaagcta cccatcatcg

TaBx4D_(AB042627.1) gcag...... ...ctcccgc ctacacctcc aggcaagctg cccatcatcg

TaBx4B_(AB124855.1) gcag...... ...ctcccgc ctacacctcc aggcaagctg cctatcatcg

ZmBx4_(X81827.1) gcacagg... ...ctgccgc cgacgcctcc ggggaagctc cccatcatcg

ScBx5_(KF636825) CCGT...... ...CTCCCGC CGTCGCCTCC GGGCAGCCTT CCCATCATCG

TaBx5D_(AB124857.1) gcgg...... ...ctcccgc cttcgcctcc gggcagcctt cccatcatcg

TaBx5A_(AB042629.1) gcgg...... ...ctcccgc cttcgcctcc gggcagcctt cccatcatcg

HlBx5_(AY462230.1) gcgg...... ...ctcccgc cttcgcctcc gggcagcctt cccatcatcg

TaBx5B_(AB124856.1) gcgg...... ...ctcccgc cttcgcctcc gggcagcctc cccatcatcg

ZmBx5_(X81830.1) gccg...... ...ctccctc cctcgccgcc gggcaccctc cccatcgtgg

ScBx5_Picasso_(HG380519.1) CCGT...... ...CTCCCGC CTTCGCCTCC GGGCAGCCTT CCCATCATCG

251 300

ScBx2_L318_(KF620524) GGCACCTGCA CCTAATGGGG GATCTCCCCT ACGTCTCCCT CGCCGGCCTG

ScBx2_(JX442061.1) ggcacctgca cctaatgggg gatctcccct acgtctccct cgccggcctg

ScBx2_Picasso_(HG380516.1) GGCACCTGCA CCTAATGGGG GATCTCCCCT ACGTCTCCCT CGCCGGCCTG

ZmBx2_(X81831.1) ggcacctgca cctgatgggg gacatcccct acgtgtccct cgccgcgctc

HlBx2_(AY462227.1) ggcacctgca cctaatgggc gatctcccct acgtctccct cgccggcctg

TaBx2A_(AB042630.1) ggcacctgca cctaatgggc gatctcccct acgtctccct cgccggcctg

TaBx2B_(AB042631.1) ggcacctgca cctaatgggc gatcttccct acgtctccct cgctggcctg

TaBx2D_(AB124851.1) ggcacctaca cctaatgggc gatcttccct acgtctccct cgccggcctg

ScBx3_(KF636827) GCCACCTCCA CCTCCTCGGC TCCCAGACAC ACATATCCAT CCGGGACCTC

ScBx3_Picasso_(HG380517.1) GCCACCTCCA CCTCCTCGGC TCCCAGACAC ACATATCCAT CCGGGACCTC

TaBx3B_(AB124853.1) gccacctcca cctcctcggc tcccagacac acatatccat ccgggacctc

TaBx3D_(AB124852.1) gccacctcca cctcctcggc tcccagacac acatatccat ccgggacctc

TaBx3A_(AB042628.1) gccacctcca cctcctcggc tcccagacac acatatccat ccgggacctc

ZmBx3_(X81829.1) gccacctcca cctcatcggc gccgagaccc acatctcgat ccgggacctg

HlBx3_(AY462228.1) gccacctcca cctcctcggc tcccagacac acatatccat cagggacctt

ScBx4_(KF636826) GCCACCTCCA CCTCATCGGC TCCCACCCCC ACGTCTCCTT CCGCGACCTC

ScBx4_Picasso_(HG380518.1) GCCACCTCCA CCTCATCGGC TCCCACCCCC ACGTCTCCTT CCGCGACCTC

HlBx4_(AY462229.1) gccacctcca cctcatcggc tcccaccctc atgtcacctt ccgcgacctc

TaBx4A_(AB124854.1) gccacctcca ccttatcggc tcccaccccc acgtctcatt ccgcgacctc

TaBx4D_(AB042627.1) gccacctcca cctcatcggc tcccaccccc acgtatcctt ccgcgacctc

TaBx4B_(AB124855.1) gccacctcca cctcatcggt tcccaccccc atgtctcctt ccgcgacctc

ZmBx4_(X81827.1) ggcacctcca cctcatcggc tcccacccgc acgtctcctt ccgcgacctc

ScBx5_(KF636825) GCCACCTCCA CCACATCGGC GCCCAGACCC ACATCTCCCT CCAGCACCTG

TaBx5D_(AB124857.1) gccacctcca ccacatcggc gcccagaccc acatctccct gcagcacctg

TaBx5A_(AB042629.1) gtcacctcca ccacatcggc gtccagaccc acatctccct ccagcacctg

HlBx5_(AY462230.1) gccacctcca ccacatcggc gcccagaccc acatctccct ccagcacctg

TaBx5B_(AB124856.1) gccacctcca ccacatcggc gcccagaccc atatctccct ccagcacctg

ZmBx5_(X81830.1) ggcacctaca ccacatcggg ccccagaccc acatctcgct ccaggagctg

ScBx5_Picasso_(HG380519.1) GCCACCTCCA CCACATCGGC GCCCAGACCC ACATCTCCCT CCAGCACCTG

301 350

ScBx2_L318_(KF620524) GCCGCCAAGT ACGGCCCGGA A...CTCATG CTGGTACACC TCGGTGCCGT

ScBx2_(JX442061.1) gccgccaagt acggcccgga a...cttatg ctggtacacc tcggtgccgt

ScBx2_Picasso_(HG380516.1) GCCGCCAAGT ACGGCCCGGA A...CTCATG CTGGTACACC TCGGTGCCGT

ZmBx2_(X81831.1) gccaccaggt acggcccgga c...ctgatg ctgctccgcc tcggcgccgt

HlBx2_(AY462227.1) gctgcaaagt acggcccgga a...ctcatg ctggtacgcc tcggtgccgt

TaBx2A_(AB042630.1) gccgccaagt acggcccgga a...ctcatg ctggtacacc tcggtgccgt

TaBx2B_(AB042631.1) gccgccaagt acggcccgga a...ctcatg ctggtacacc tcggtgccgt

TaBx2D_(AB124851.1) gccgccaagt acggcccgga a...ctcatg ctggtacacc tcggtgccgt

ScBx3_(KF636827) GATGCCAAGC ATGGTCGCAA TGGCCTCTTG CTCCTCCGCA TCGGTGCTGT

ScBx3_Picasso_(HG380517.1) GATGCCAAGC ATGGTCGCAA TGGCCTCTTG CTCCTCCGCA TCGGTGCTGT

TaBx3B_(AB124853.1) gatgccaagc atggccgcaa tggcctcttg ctcctccgca tcggtgccgt

TaBx3D_(AB124852.1) gatgccaagc atggccgcaa tggcctcttg ctcctccgca tcggtgccgt

TaBx3A_(AB042628.1) gatgccaagc atggccgcaa tggcctcttg ctcctccgca tcggtgccgt

ZmBx3_(X81829.1) gacgccaagc acgggcgcaa cggcctgctg ctcctccgca tcggcgccgt

HlBx3_(AY462228.1) gatgccaagc atggccgtaa tggcctcttg ctcctccgca tcggtgccgt

ScBx4_(KF636826) GCCGCCAAGC ATGGCCGCGA CGGCCTCATG CTCGTCCATG TCGGTGCCGT

ScBx4_Picasso_(HG380518.1) GCCGCCAAGC ATGGCCGCGA CGGCCTCATG CTCGTCCATG TCGGTGCCGT

HlBx4_(AY462229.1) gccgcaaagc atggccgtga cggcctcatg ctcgtccatg tcggcgccgt

TaBx4A_(AB124854.1) gccgcaaagc atggccgcga cggcctcatg ctcgtccatg tcggtgccgt

TaBx4D_(AB042627.1) gccgcaaagc atggccgcga cggcctcatg ctcgtccatg tcggtgcggt

TaBx4B_(AB124855.1) gccgcaaagc atggccgcga cggcctcatg ctcgtccatg tcggtgccgt

ZmBx4_(X81827.1) cacgccaagt acggccacaa cgggctgatg ctggtgcagg tcggcgccgt

ScBx5_(KF636825) GTCGACAAGT ACGGCCACAA CGGCCTCCTC TTCCTCCGCG CCGGCGCCGT

TaBx5D_(AB124857.1) gtcgacaagt acggccacaa cggcctcctc ttcctccgcg ccggcgccgt

TaBx5A_(AB042629.1) gtcgacaagt acggccacaa cggcctcctc ttcctccgcg ccggcgccgt

HlBx5_(AY462230.1) gtcgacaagt acggccacaa cggcctcctc ttcctccgcg ccggcgccgt

TaBx5B_(AB124856.1) gtcgacaagt acggccacaa cggcctcctc ttcctccgtg ctggggcggt

ZmBx5_(X81830.1) gtggccaagt acgggcacaa cgggttcctg ttcctccgcg ccggcgccgt

ScBx5_Picasso_(HG380519.1) GTCGACAAGT ACGGCCACAA CGGCCTCCTC TTCCTCCGCG CCGGCGCCGT

351 400

ScBx2_L318_(KF620524) GCCCACCGCC GTCGTGTCCT CACCGCGCAC TGCCGAGGCC ATCCTGCGCA

ScBx2_(JX442061.1) gcccaccgcc gtcgtgtcct caccgcgcac tgccgaggcc atcctgcgca

ScBx2_Picasso_(HG380516.1) GCCCACCGCC GTCGTGTCCT CACCGCGCAC TGCCGAGGCC ATCCTGCGCA

ZmBx2_(X81831.1) gccgaccgtc gtggtctcgt cgccgcgcgt cgccgaggcc gtgctccgca

HlBx2_(AY462227.1) gcccactgcc gtcgtgtcct ccccgcgcac tgcggaggcc gtcctgcgca

TaBx2A_(AB042630.1) gcccaccgcc gtcgtgtcct cgccgcgcac tgccgaggcc gtcctgcgca

TaBx2B_(AB042631.1) gcccaccgcc gtcgtgtcct cgccgcgcac tgctgaggcc gtcctgcgca

TaBx2D_(AB124851.1) gcccaccgcc gtcgtgtcct cgccgcgcac tgccgaggcc gtcctgcgca

ScBx3_(KF636827) ACCCACCTTG TTCGTGTCCT CGCCGAGCGC CGCCGAGGCC GTCCTGCGCA

ScBx3_Picasso_(HG380517.1) ACCCACCTTG TTCGTGTCCT CGCCGAGCGC CGCCGAGGCC GTCCTGCGCA

TaBx3B_(AB124853.1) acccaccttg ttcgtgtcct cgccgagcgc cgccgaggcc gtcctgcgca

TaBx3D_(AB124852.1) acccacctta ttcgtgtcct cgccgagtgc cgccgaggct gtcctgcgta

TaBx3A_(AB042628.1) acccaccttg ttcgtgtcct cgccgagcgc cgccgaggcc gtcctgcgca

ZmBx3_(X81829.1) gcccaccctg ttcgtgtcct cgcccagcgc cgccgacgcc gtcctgcgca

HlBx3_(AY462228.1) acccaccttg ttcgtgtcct cgccgagcgc cgccgaggcc gtcctgcgca

ScBx4_(KF636826) GCCCACTGTG GTGGTGTCCA CGCCTCAGGC CGCCGAGGCC GTCCTCCGCA

ScBx4_Picasso_(HG380518.1) GCCCACTGTG GTGGTGTCCA CGCCTCAGGC CGCCGAGGCC GTCCTCCGCA

HlBx4_(AY462229.1) gcccaccgtc gtcgtgtcca cacctcaggc cgccgaggcc gtcctccgca

TaBx4A_(AB124854.1) gcccaccgtc gttgtgtcca cgccccaggc cgctgaggct gtcctgcgca

TaBx4D_(AB042627.1) gcccaccgtg gtggtgtcca cacctcaggc cgccgaggcc gtcctgcgca

TaBx4B_(AB124855.1) gcccaccgtc gtcgtgtcca cgccccaggc cgccgaggct gtcctgcgca

ZmBx4_(X81827.1) gcccaccatc gtggtgtcca cgccgcaggc cgccgaggcc gtgctccgca

ScBx5_(KF636825) GCCCACCGTC ATCGTCTCCT CGCCCAGCGC CGCCGAGGCC GTCATGCGCA

TaBx5D_(AB124857.1) gcccaccgtc atcgtctcct cgccaagcgc cgccgaggcc gtcatgcgca

TaBx5A_(AB042629.1) gcccaccgtc atcgtctcct cgcccagcgc cgccgaggct gtcatgcgca

HlBx5_(AY462230.1) gcccaccgtc atcgtgtcct cgcccagcgc cgccgaggcc gtcatgcgca

TaBx5B_(AB124856.1) gcccaccgtc atcgtctcct cgcccagcgc cgccgaggcc gtcatgcgca

ZmBx5_(X81830.1) gcccaccctg atcgtgtcgt cgcccagcgc cgccgaggct gtgatgcgca

ScBx5_Picasso_(HG380519.1) GCCCACCGTC ATCGTCTCCT CGCCCAGCGC CGCCGAGGCC GTCATGCGCA

401 450

ScBx2_L318_(KF620524) CCCACGACCA CATCTTCGCG TCGCGGCCGC GGTCGATGGT CTTCGACATC

ScBx2_(JX442061.1) cccacgacca catcttcgcg tcgcggccgc ggtcgatggt cttcgacatc

ScBx2_Picasso_(HG380516.1) CCCACGACCA CATCTTCGCG TCGCGGCCGC GGTCGATGGT CTTCGACATC

ZmBx2_(X81831.1) cctacgacca cgtcttctcc tcccgcccgc gctccctggt gtccgacatc

HlBx2_(AY462227.1) cccacgacca cgtcttcgcg tcgcggccgc ggtcgatggt gttcgacatc

TaBx2A_(AB042630.1) cccacgacca catcttcgcg tcacggccgc ggtcgatggt cttcgacatc

TaBx2B_(AB042631.1) cccacgacca catcttcgca tcgcggccgc ggtcgatggt cttcgacatc

TaBx2D_(AB124851.1) cccacgacca catcttcgcg tcgcgaccgc ggtcgatggt cttcgacatc

ScBx3_(KF636827) CCCACGACCA AATCTTTGCG TCGCGGCCGC CATCCATGGC CGCTGACATC

ScBx3_Picasso_(HG380517.1) CCCACGACCA AATCTTTGCG TCGCGGCCGC CATCCATGGC CGCTGACATC

TaBx3B_(AB124853.1) cccacgacca aatctttgcg tcgcggccgc catccatggc cgccgacatc

TaBx3D_(AB124852.1) cccacgacca aatctttgcg tcgcggccac catccatggc cgctgacatc

TaBx3A_(AB042628.1) cccacgacca aatctttgcg tcgcggccgc catccatggc cgccaacatc

ZmBx3_(X81829.1) cccaggacca catcttcgcg tcgcggccgc cgtggatggc cgccgagatc

HlBx3_(AY462228.1) cccacgacca aatctttgcg tcgcggccgc catccatggc cgccgccatc

ScBx4_(KF636826) CGCACGACCA TGTGTTTGCA TCCAGGCCAC GTAACCCCGT CGCCGACATC

ScBx4_Picasso_(HG380518.1) CGCACGACCA TGTGTTTGCA TCCAGGCCAC GTAACCCCGT CGCCGACATC

HlBx4_(AY462229.1) cgcacgacca cgtgttcgcg tccaggccac gaaaccctgt cgccgacatc

TaBx4A_(AB124854.1) cgcacgacca cgtgttcgca tccaggccac gtaaccccgt cgccgacatc

TaBx4D_(AB042627.1) cacacgacca cgtgttcgcg tccaggccac gtaaccccgt cgccgacatc

TaBx4B_(AB124855.1) cgcacgacca cgtgttcgcg tccaggccac gtaaccccgt cgccgacatc

ZmBx4_(X81827.1) cgcacgacca cgtgctggcg tcccgcccgc ggaaccccgt cgccgacatc

ScBx5_(KF636825) CCCACGACCA CATCCTCGCC TCCCGCCCGT GGTCCATGGC CTCGCACATC

TaBx5D_(AB124857.1) cccacgacca catcctcgcg tcgcgcccgt ggtccatggc ctcgcacatc

TaBx5A_(AB042629.1) cgcacgacca catcctcgcg tcccgcccgt ggtccatggc ctcgcacatc

HlBx5_(AY462230.1) ctcacgacca catcctcgca tcgcgcccgt ggtccatggc ctcccacatc

TaBx5B_(AB124856.1) cccacgacca catcctcgcg tcgcgcccgt ggtccatggc ctcccacatc

ZmBx5_(X81830.1) cccacgacca catctgcgcg tcccggccgt ggtccatggc ctcccacatc

ScBx5_Picasso_(HG380519.1) CCCACGACCA CATCCTCGCC TCCCGCCCGT GGTCCATGGC CTCGCACATC

451 500

ScBx2_L318_(KF620524) ATCATGTACG GGCAGACGGA CTCATGCTTC GCGCCCTACG GCGAGCACTT

ScBx2_(JX442061.1) atcatgtacg ggcagacgga ctcatgcttc gcgccctacg gcgagcattt

ScBx2_Picasso_(HG380516.1) ATCATGTACG GGCAGACGGA CTCATGCTTC GCGCCCTACG GCGAGCACTT

ZmBx2_(X81831.1) atcatgtacg gcgccaccga ctcgtgcttc gcgccctacg gcgaccactt

HlBx2_(AY462227.1) atcatgtacg ggcagacgga ctcgtgcttc gcgccctacg gcgaccactt

TaBx2A_(AB042630.1) atcatgtacg ggcagacgga ctcgtgcttc gcgccctacg gcgagcactt

TaBx2B_(AB042631.1) atcatgtacg ggcagacgga ctcgtgcttc tcgccctacg gcgagcactt

TaBx2D_(AB124851.1) atcatgtacg ggcagacgga ctcgtgcttc gcgccctacg gcgagcactt

ScBx3_(KF636827) ATTCGCTACG GCCCCACAGA CATTGCGTTT GCACCCTATG GCGAGTACTG

ScBx3_Picasso_(HG380517.1) ATTCGCTACG GCCCCACAGA CATTGCGTTT GCACCCTATG GCGAGTACTG

TaBx3B_(AB124853.1) attcgctacg gcccgacaga catcgcgttt gcaccctatg gtgagtactg

TaBx3D_(AB124852.1) attcgctacg gccccacaga catcgcattt gcaccctatg gtgagtactg

TaBx3A_(AB042628.1) attcgctatg ggccaacaga catcgcgttt gcaccctatg gcgagtactg

ZmBx3_(X81829.1) atccgctacg ggccctcgga cgtcgcgttc gtgccctacg gcgagtacgg

HlBx3_(AY462228.1) attcgctacg gcctgacaga catcgctttt gcaccctatg gtgagtactg

ScBx4_(KF636826) ATCCGCTACA ACTCCACAGA CATCGCGTTC GCGCCCTACG GCGACTACTG

ScBx4_Picasso_(HG380518.1) ATCCGCTACA ACTCCACAGA CATCGCGTTC GCGCCCTACG GCGACTACTG

HlBx4_(AY462229.1) atccgctacg gctccacgga catcgcgttc gcgccctatg gcgactattg

TaBx4A_(AB124854.1) atccgctaca actccacaga catcgcgttc gcgccctacg gcgactactg

TaBx4D_(AB042627.1) atccgctaca actccactga catcgcattc gcgccctacg gcgactactg

TaBx4B_(AB124855.1) atccgctaca actccacaga catcgcgttc gcgccctacg gcgactactg

ZmBx4_(X81827.1) atccgctaca actgcacgga cgtggccttc gcgccgtacg gcgagtactg

ScBx5_(KF636825) CTTCGCTACA ACACCACCGA CGTCGCCTTC TCGCCCTTGG GCGAGTACTG

TaBx5D_(AB124857.1) cttcgctaca acaccactga cgtcgccttc tcgcccttgg gcgagtactg

TaBx5A_(AB042629.1) ctgcgctaca acaccaccga cgtcgctttc tcacccttgg gcgagtattg

HlBx5_(AY462230.1) cttcgctaca acaccaccga cgtcgccttc tcgcccttgg gcgagtactg

TaBx5B_(AB124856.1) ctgcgctaca acaccaccga cgtcgccttc tcgcccctgg gcgagtactg

ZmBx5_(X81830.1) ctccgctaca acacctgcga cgtggccttc tcgccgctcg gcgaatactg

ScBx5_Picasso_(HG380519.1) CTTCGCTACA ACACCACCGA CGTCGCCTTC TCGCCCTTGG GCGAGTACTG

501 550

ScBx2_L318_(KF620524) CCGGAAGGCC AGGAAGCTCG TGACGGTGCA CATGCTCAAC GCCAGGAAGA

ScBx2_(JX442061.1) ccggaaggcc aggaagctcg tgacggtgca catgctcaac gccaggaaga

ScBx2_Picasso_(HG380516.1) CCGGAAGGCC AGGAAGCTCG TGACGGTGCA CATGCTCAAC GCCAGGAAGA

ZmBx2_(X81831.1) ccgcaaggcg cggaagctgg tcaccgtgca cctgctcaac gccagcaagg

HlBx2_(AY462227.1) ccggaaggcc aggaagcttg tgacggtgca catgctcaac gccaggaaga

TaBx2A_(AB042630.1) ccggaaggcc aggaagctgg tgacggtgca catgctcaac gccaggaaga

TaBx2B_(AB042631.1) ccggaaggcc aggaagctcg tgacggtgca catgctcaac gccaggaaga

TaBx2D_(AB124851.1) ccggaaggcc aggaagctcg tgacggtgca catgctcaac gccaggaaga

ScBx3_(KF636827) GCGGCAGGCC AGGAAGCTCT TAACCACGCA TATGCTCAGC GCCAAGGTGG

ScBx3_Picasso_(HG380517.1) GCGGCAGGCC AGGAAGCTCT TAACCACGCA TATGCTCAGC GCCAAGGTGG

TaBx3B_(AB124853.1) gcggcaggcc aggaagctgt taaccacgca tatgctcagc gccaaggtgg

TaBx3D_(AB124852.1) gcggcaggcc aggaagctct taaccacaca tatgctcagc gccaaggtgg

TaBx3A_(AB042628.1) gcggcaggcc aggaagctct taaccacaca tatgctcagc gcgaaggtgg

ZmBx3_(X81829.1) gcggcagggc aggaaactcc tcaccacgca catgctcagc accaagaagg

HlBx3_(AY462228.1) gcggcaggcc aggaagctct taaccacgca tatgctcagc gccaaggtgg

ScBx4_(KF636826) GCGTAGGGCT AGGAAGGTCG TCAACACGCA TCTGCTCAGC GTCAAGATGG

ScBx4_Picasso_(HG380518.1) GCGTAGGGCT AGGAAGGTCG TCAACACGCA TCTGCTCAGC GTCAAGATGG

HlBx4_(AY462229.1) gcgtagggct aggaaggtcg tcaacacgca tctgctcagc gtcaagatgg

TaBx4A_(AB124854.1) gcgtagggct aggaaggtcg tcaacacgca tctgctcagc gtcaagatgg

TaBx4D_(AB042627.1) gcgtagggct aggaaggtcg tcaacacgca tctgctcagc gtcaagatgg

TaBx4B_(AB124855.1) gcgtagggct aggaaggtcg tcaacacgca tctgctcagt gtcaagatgg

ZmBx4_(X81827.1) gcgcacggcg cggaaggtgg tcaacacgca cctgctcagc gccaagatgg

ScBx5_(KF636825) GCAGCACACC CGGAAGCTCG TCAACACCCA TCTCCTCAAC GCCAAGAAGG

TaBx5D_(AB124857.1) gcagcacacc cggaagctcg tcaacacgca cctgctcagt gccaagaagg

TaBx5A_(AB042629.1) gcagcacacc aggaagctcg tcaacaccca tctcctcagc gccaagaagg

HlBx5_(AY462230.1) gcagcacacc aggaagctcg tcaacaccca cctgctcagc gccaagaagg

TaBx5B_(AB124856.1) gcagcacacc aggaagctcg tcaacaccca tctcctcagc gccaagaagg

ZmBx5_(X81830.1) gcagcagacc aggaagctga tgaacacgca cctgctcagc aacaagaagg

ScBx5_Picasso_(HG380519.1) GCAGCACACC CGGAAGCTCG TCAACACCCA TCTCCTCAAC GCCAAGAAGG

551 600

ScBx2_L318_(KF620524) TTCGGTCCCA GCGCCCGGCC CGGGAAGAGG AGGTCCGGCT GGTGATTGGG

ScBx2_(JX442061.1) ttcggtccca gcgcccggcc cgggaagagg aggtccggct ggtgattggg

ScBx2_Picasso_(HG380516.1) TTCGGTCCCA GCGCCCGGCC CGGGAAGAGG AGGTCCGGCT GGTGATTGGG

ZmBx2_(X81831.1) tgcggtcgca gcggcccgcg cgggaggagg aggtgcgcgg cgcgctcgac

HlBx2_(AY462227.1) taaggtccca acgcccggcc cgcgaagagg aggtccggct ggtgattgga

TaBx2A_(AB042630.1) tacggtccca gcgcccggcc cgggtggagg aggtccggct agtgattgga

TaBx2B_(AB042631.1) tacggtccca gcgcccggcc cgggaagagg aggtccggca agtgattgga

TaBx2D_(AB124851.1) tacggtccca gcgcccggcc cgggaggagg aggtccggct agtgattgga

ScBx3_(KF636827) TGCACTCCTT CCGCCATGGT CGTCAAGAAG AGGTGCGCCT CGTTATCAAC

ScBx3_Picasso_(HG380517.1) TGCACTCCTT CCGCCATGGT CGTCAAGAAG AGGTGCGCCT CGTTATCAAC

TaBx3B_(AB124853.1) tgcactcctt ccgccatggt cgtcaggaag aggtgcgcct tgttatcaac

TaBx3D_(AB124852.1) tgcactcctt ccgccatggt cgtcaagaag aggtgcgcct cgttatcaac

TaBx3A_(AB042628.1) tgcactcctt ccgccatggt cgtcaagaag aggtgcgcct cattatcaac

ZmBx3_(X81829.1) tgcagtcctt ccgccatggc cgccaggaag aggtgcggct cgtcatggac

HlBx3_(AY462228.1) tgcactcctt ccgccatggt cgtcaggaag aggtgcgcct tgttatcaac

ScBx4_(KF636826) TCTACTCAAA GCGCCATGAC CGTGAAGAAG AGGTGCGGCT CGTGGTCGGC

ScBx4_Picasso_(HG380518.1) TCTACTCAAA GCGCCATGAC CGTGAAGAAG AGGTGCGGCT CGTGGTCGGC

HlBx4_(AY462229.1) tctactccaa gcgccatgac cgcgaagaag aggtgcggct cgtggtcgcc

TaBx4A_(AB124854.1) tctactccaa gcgccatgac cgcgaagaag aggtgcggct cgtggtcgcc

TaBx4D_(AB042627.1) tctactccaa gcgccatgac cgcgaagagg aggtgcggct cgtggtcgcc

TaBx4B_(AB124855.1) tctactccaa gcgccatgac cgcgaagaag aggtgcggct cgtggtcgcc

ZmBx4_(X81827.1) tcttctccaa gcggcgggag cgggaggagg aggtgcgcct ggtggtggcc

ScBx5_(KF636825) TGCACTCCTT CCGCCATGGC CGTCAAGAAG AGGTGTGCCT CGTCGTCAAC

TaBx5D_(AB124857.1) tgcactcctt ccgccatggc cgtcaagaag aggtgtgcct cgtcgtcaac

TaBx5A_(AB042629.1) tgcactcctt ccgccatggc cgtcaagaag aggtgtgcct cgtcgtcaac

HlBx5_(AY462230.1) tgcactcctt ccgccatggc cgtcaagaag aggtgagcct cgtcgttgac

TaBx5B_(AB124856.1) tgcactcctt cgcaaatggc cgtcaagaag aggtgtgcct cgtcgtcaac

ZmBx5_(X81830.1) tctactcctt ccgccatggc cgcgaggaag aggtgtgcct cgtcgtggac

ScBx5_Picasso_(HG380519.1) TGCACTCCTT CCGCCATGGC CGTCAAGAAG AGGTGTGCCT CGTCGTCAAC

601 650

ScBx2_L318_(KF620524) AAGGTCGCCA AGGCCGCGGC CGCGCGC... ...GAGTCCG TCGACATGAG

ScBx2_(JX442061.1) aaggtcgcca aggccgcggc cgcgcgc... ...gagtccg tggacatgag

ScBx2_Picasso_(HG380516.1) AAGGTCGCCA AGGCCGCGGC CGCGCGC... ...GAGTCCG TGGACATGAG

ZmBx2_(X81831.1) agggtgcgcc gcgccgccgc cgcgcgg... ...gagcccg tcgacatgag

HlBx2_(AY462227.1) aagatcgcca aggccgcggc cgcgcgc... ...gaggccg tggacatgag

TaBx2A_(AB042630.1) aagatcgcca aggccgcggc cgcgcgc... ...gaggccg tggacatgag

TaBx2B_(AB042631.1) aagatcgcca aggccgcagc cgcgcgc... ...gaggccg tggacatgag

TaBx2D_(AB124851.1) aagatcgcca aggccgcggc cgcgcgc... ...gaggccg tagacatgag

ScBx3_(KF636827) AAGATCCGTG CGGCGGCCAC CAGAGGC... ...ACCGCGG TGGACATGAG

ScBx3_Picasso_(HG380517.1) AAGATCCGTG CGGCGGCCAC CAGAGGC... ...ACCGCGG TGGACATGAG

TaBx3B_(AB124853.1) aagatccgtg aggcggccac cagaggc... ...acggtgg tggacatgag

TaBx3D_(AB124852.1) aagatccatg aggcggccac cagaggc... ...acggcgg tggacatgag

TaBx3A_(AB042628.1) aagatccgtg cggcggccac cagaggc... ...acggcgg tggacatgag

ZmBx3_(X81829.1) aagatccggg cggctgccac ggcggccccg ccggcggcgg tggacctgag

HlBx3_(AY462228.1) aagacccgtg aggcggccac cagaggc... ...acggcgg tggacatgag

ScBx4_(KF636826) AAGATCCGTG AGTTGGCCAT GGCCGCTCCA GGCAAGGCAT TGGACATGAC

ScBx4_Picasso_(HG380518.1) AAGATCCGTG AGTTGGCCGC GGCCGCTCCA GGCAAGGCAT TGGACATGAC

HlBx4_(AY462229.1) aagatccatg agttggccat ggccgctcca ggcaaggcgt tggacatgac

TaBx4A_(AB124854.1) aagatccgtg agttggccac agctgctcca ggcaaggcat tggacatgac

TaBx4D_(AB042627.1) aagatctgtg agttggccat ggccgctcca ggcaaggcgt tggacatgac

TaBx4B_(AB124855.1) aagatccgtg agttggccat ggccgctcca ggcaaggcgt tggacatgac

ZmBx4_(X81827.1) cggatccgcg acgccgccga ggcgtccccg gggacggcgc tcgacatgac

ScBx5_(KF636825) AAGATTCGTG AGGCGGCCAC CAACGCTCCA TCCACGGCGG TGGACATGAG

TaBx5D_(AB124857.1) aagattcgtg aggcggccac caacgctcca tccacggcgg tggacatgag

TaBx5A_(AB042629.1) aagattcacg aggcggccac caacgctcca tccacggcgg tggacatgag

HlBx5_(AY462230.1) aagatccgtg aggcggccac caacgctcca tccaccgtgg tggatatgag

TaBx5B_(AB124856.1) aagattcgtg aggcggccac cactgctcca tccacggcgg tggacatgag

ZmBx5_(X81830.1) aacctccgcg aggcggccgc caagtcgccg tcgacggccg tggacatgag

ScBx5_Picasso_(HG380519.1) AAGATTCGTG AGGCGGCCAC CAACGCTCCA TCCACGGCGG TGGACATGAG

651 700

ScBx2_L318_(KF620524) CGAGCTCCTG CACTCGTACG TCAATGACCT CGTTTGCCGT GCTGTGTCGG

ScBx2_(JX442061.1) cgagctcctg cactcgtacg tcaatgacct cgtttgccgt gctgtgtcgg

ScBx2_Picasso_(HG380516.1) CGAGCTCCTG CACTCGTACG TCAATGACCT CGTTTGCCGT GCTGTGTCGG

ZmBx2_(X81831.1) cgagctcctg cactccttcg tcaacaacct cgtctgccgc gccgtctccg

HlBx2_(AY462227.1) cgagctcctg cactcgtacg tcaacgacct cgtttgccgt gctgtgtcgg

TaBx2A_(AB042630.1) cgagctcctg cactcgtacg tcaacgacct cgtttgccgt gctgtgtcgg

TaBx2B_(AB042631.1) tgagctcctg cactcgtacg tcaacgacct cgtttgccgt gctgtgtcgg

TaBx2D_(AB124851.1) cgagctcctg cactcgtacg tcaacgacct cgtttgccgt gctgtgtcgg

ScBx3_(KF636827) CGAGCTCCTG TCCGGCTACA CCAATGACGT CGTGTGCCGT GCAGTCCTTG

ScBx3_Picasso_(HG380517.1) CGAGCTCCTG TCCGGCTACA CCAATGACGT CGTGTGCCGT GCAGTCCTTG

TaBx3B_(AB124853.1) cgacctcctg tccggctaca ccaacgacgt cgtgtgccgt gcggtcctcg

TaBx3D_(AB124852.1) cgagctcctg tccggctaca ccaacgacgt cgtatgccgt gcagtcctcg

TaBx3A_(AB042628.1) cgagctcctg tccggctaca ccaatgacgt cgtatgccgt gcggtcctag

ZmBx3_(X81829.1) cgatctcctg tccgggtaca ccaacgacgt ggtgtcccgc gcggtgcttg

HlBx3_(AY462228.1) cgagctcctg tccggctaca ccaatgacgt cgtatgccgt gcagtcctcg

ScBx4_(KF636826) AGAGCTCCTG GGTGGGTACG CCAGCGACTT TGTGTGCCGT GCGGTCCTCG

ScBx4_Picasso_(HG380518.1) GGAGCTCCTG GGTGGGTACG CCAGCGACTT TGTGCGCCGT GCGGTCCTCG

HlBx4_(AY462229.1) ggagctcttg ggcgggtacg ccagcgactt tgtgtgccgt gcggtcctcg

TaBx4A_(AB124854.1) ggagctcctg ggcgggtacg ccagcgactt tgtgtgccgt gcggtccttg

TaBx4D_(AB042627.1) ggagctcctg ggcgggtatg ccagcgactt tgtgtgccgt gcggtcctcg

TaBx4B_(AB124855.1) agagctcctg ggcgggtatg ccagcgactt tgtgtgccgt gcggtcctcg

ZmBx4_(X81827.1) ggagctgctg ggcggctacg ccagcgactt cgtgtgccgc gccgtgctcg

ScBx5_(KF636825) CGAGTTCCTG GCCGCCTACA CCAACGACGT CGTAAGCCGC TCGGTGCTGG

TaBx5D_(AB124857.1) cgagttcctg gccgcctaca ccaatgacgt cgtaagccgc tcggtgctgg

TaBx5A_(AB042629.1) cgagttcctg gccgcctaca ccaacgatgt cgtaagccgc tcggtgctgg

HlBx5_(AY462230.1) cgagtttctg gccgcctaca ccaatgatgt cgtaagccgc tcggtgctgg

TaBx5B_(AB124856.1) cgagtttctg gccgcctaca ccaacgatgt tgtaagccgc tcggtgctgg

ZmBx5_(X81830.1) cgaggtgctg gcggcgtaca ccaacgacgt ggtgagccgg tcggtgctgg

ScBx5_Picasso_(HG380519.1) CGAGTTCCTG GCCGCCTACA CCAACGACGT CGTAAGCCGC TCGGTGCTGG

701 750

ScBx2_L318_(KF620524) GCAAGTTCTC CCAGGAGGAG GGGCGGAACA AGCTGTTCCG TGAGCTCACC

ScBx2_(JX442061.1) gcaagttctc ccaggaggag gggcggaaca agctgttccg tgagctcacc

ScBx2_Picasso_(HG380516.1) GCAAGTTCTC CCAGGAGGAG GGGCGGAACA AGCTGTTCCG TGAGCTCACC

ZmBx2_(X81831.1) gcaagttctc catggaggaa ggccgcaaca ggctgttccg ggagctcacc

HlBx2_(AY462227.1) gtaagttctc acaggaggag gggcggaaca agctgttccg tgagctcacc

TaBx2A_(AB042630.1) gcaagttctc ccaggaggag gggcggaaca agctgttccg tgagctcacc

TaBx2B_(AB042631.1) gcaagttctc ccaggaggag gggcggaaca agctgttccg agagctcacc

TaBx2D_(AB124851.1) gcaagttctc ccaggaggag gggcggaaca agctgttccg tgagctcacc

ScBx3_(KF636827) GAGAATCCCA CCGCAAGGCA GGCCGGAACA GGCTTTTCAG TGAGCTCACA

ScBx3_Picasso_(HG380517.1) GAGAATCCCA CCGCAAGGCA GGCCGGAACA GGCTTTTCAG TGAGCTCACA

TaBx3B_(AB124853.1) gagaatccca ccgcaaggca ggccggaaca ggcttttcag cgagctcaca

TaBx3D_(AB124852.1) gagaatccca ccgcaaggaa ggccggaaca ggcttttcag cgagctcaca

TaBx3A_(AB042628.1) gagaatccca ccgcaaggaa ggccggaaca ggcttttcag cgagctcaca

ZmBx3_(X81829.1) gcgcgtcgca ccggaaccaa gggcggaaca ggctgttcag cgagctgaca

HlBx3_(AY462228.1) gagaatcaca ccgcaaggaa ggccggaaca ggcttttcag cgagctcacg

ScBx4_(KF636826) GAGAGTCCCA CCGGAAGCAT GGCCGGAACG AGCTTTTCCG CGAGCTCACC

ScBx4_Picasso_(HG380518.1) GAGAGTCCCA CCGGAAGCAT GGCCGGAACG AGCTTTTCCG CGAGCTCACC

HlBx4_(AY462229.1) gagagtccca ccggaagcat ggccggaacg agcttttccg cgagctcacc

TaBx4A_(AB124854.1) gagagtccca ccggaagaat ggccggaacg agcttttccg cgagctcacc

TaBx4D_(AB042627.1) gagagtccca caggaagcat ggccggaacg agcttttccg cgagctcacc

TaBx4B_(AB124855.1) gagagtccca caggaagcat ggccggaacg agcttttccg cgagctcacc

ZmBx4_(X81827.1) gcgagtccca ccggaagcag ggccggaaca agctgttccg ggagctgacg

ScBx5_(KF636825) GAGCGACCCA CCGGAAGAAA GGCCGCAACA CTCTCTTCAG AGAGATGACC

TaBx5D_(AB124857.1) gagcgaccca ccggaagaaa ggccgcaaca cgctcttcag agagatgacc

TaBx5A_(AB042629.1) gagcaaccca ccggaagaaa ggccgcaaca cgctcttcag agagatgacc

HlBx5_(AY462230.1) gagcaactca ccggaagaaa ggccgcaaca cgctcttcag agagatgacc

TaBx5B_(AB124856.1) gagcgaccca ccggaagaaa ggccgcaaca cgctcttcag agagatgacc

ZmBx5_(X81830.1) gctcgacgca ccggaagaaa ggccggaaca cgctgttcag ggagatgacc

ScBx5_Picasso_(HG380519.1) GAGCGACCCA CCGGAAGAAA GGCCGCAACA CTCTCTTCAG AGAGATGACC

751 800

ScBx2_L318_(KF620524) GACATCAACG CGGCGCTCCT GGGAGGGTTC AACATCCTCG ACTACTTCCC

ScBx2_(JX442061.1) gacatcaacg cggcgctcct gggagggttc aacatcctcg actacttccc

ScBx2_Picasso_(HG380516.1) GACATCAACG CGGCGCTCCT GGGAGGGTTC AACATCCTCG ACTACTTCCC

ZmBx2_(X81831.1) gacatcaacg cgggcctcct cggagggttc cacatccagg actacttccc

HlBx2_(AY462227.1) gacatcaacg cggcgctcct gggagggttc aacatcctcg actacttccc

TaBx2A_(AB042630.1) gacatcaacg cggcgctcct gggagggttc aacatcctcg actacttccc

TaBx2B_(AB042631.1) gacatcaacg cggcgctcct gggagggttc aacatcctcg actacttccc

TaBx2D_(AB124851.1) gacatcaacg cggcgctcct gggagggttc aacatcctcg actacttccc

ScBx3_(KF636827) GAGATCAACG TCTCCCTTCT TGGCGGGTTC AGCCTCGAGA ACTACATCCC

ScBx3_Picasso_(HG380517.1) GAGATCAACG TCTCCCTTCT TGGCGGGTTC AGCCTCGAGA ACTACATCCC

TaBx3B_(AB124853.1) gagatcaacg tctcccttct cggcgggttc agcctcgaga attacatccc

TaBx3D_(AB124852.1) gagatcaacg tctcccttct tggcgggttc agcctcgaga attacatccc

TaBx3A_(AB042628.1) gagatcaatg tctcccttct tggtgggttc agcctcgaga attacatccc

ZmBx3_(X81829.1) gagatcaacg tgtccctcct cgccgggttc aacctcgagg actacttccc

HlBx3_(AY462228.1) gagatcaacg tgtcccttct tggcgggttc agcctcgaga attacatccc

ScBx4_(KF636826) GAGATCAGCG CCTCCCTGCT GGGGGGATTC AACCTGGAGG ACTACTTCCC

ScBx4_Picasso_(HG380518.1) GAGATCAGCG CCTCCCTGCT GGGGGGATTC AACCTGGAGG ACTACTTCCC

HlBx4_(AY462229.1) gagatcagcg cctccctact agggggattc aacctggagg actacttccc

TaBx4A_(AB124854.1) gagatcagcg cctccctgct ggggggattc aacctggagg actacttccc

TaBx4D_(AB042627.1) gagatcagcg cctccctgct ggggggattc aacctggagg actacttccc

TaBx4B_(AB124855.1) gagatcagcg cctccctgct ggggggattc aacctggagg actacttccc

ZmBx4_(X81827.1) gagaccagcg ccgccctcct gggcgggttc aacgtggagg actacttccc

ScBx5_(KF636825) GAGACCAACG TCGACCTTCT CGTGGGATTC AACCTTGAGA ACTTCATCCC

TaBx5D_(AB124857.1) gagaccaacg tcgaccttct cgtgggattc aaccttgaga acttcatccc

TaBx5A_(AB042629.1) gagaccaacg ttgaccttct cgtgggattc aaccttgaga acttcatccc

HlBx5_(AY462230.1) gagaccaacg ttgaccttct agtgggattc aaccttgaga acttcatccc

TaBx5B_(AB124856.1) gagaccaacg tcgaccttct cgtgggattc aaccttgaga acttcatccc

ZmBx5_(X81830.1) atgaccaacg tggacctcct ggtggggttc aacctggagt actacatccc

ScBx5_Picasso_(HG380519.1) GAGACCAACG TCGACCTTCT CGTGGGATTC AACCTTGAGA ACTTCATCCC

801 850

ScBx2_L318_(KF620524) G...AGCCTG GGGAGGTTCG AGTTGGTCTG C...AAGGTG GCCTGCGCCA

ScBx2_(JX442061.1) g...agcctg gggaggttcg agttggtctg c...aaggtg gcctgcgcca

ScBx2_Picasso_(HG380516.1) G...AGCCTG GGGAGGTTCG AGTTGGTCTG C...AAGGTG GCCTGCGCCA

ZmBx2_(X81831.1) t...aggctg ggccggatcg agctcgtcag g...aaggtg gcgtgcgcca

HlBx2_(AY462227.1) g...agcctg gggaggttcg agttggtctg c...aaggtg gcgtgcgcca

TaBx2A_(AB042630.1) g...agcctg gggaggttcg agttggtctg c...aagatg gcctgcgcca

TaBx2B_(AB042631.1) g...agcctg gggaggttcg agttggtctg c...aaggtg gcgtgcgcca

TaBx2D_(AB124851.1) g...agcctg gggaggttcg agttggtctg c...aaggtg gcgtgcgcca

ScBx3_(KF636827) CCCAAACATG GTAATGGCGG ATGTGCTCTT G...AGGCTG GTTTCCGTCA

ScBx3_Picasso_(HG380517.1) CCCAAACATG GTAATGGCGG ATGTGCTCTT G...AGGCTG GTTTCCGTCA

TaBx3B_(AB124853.1) cccaaacatg gtaatggcgg atgtgctctt g...aggctg gtttccgtca

TaBx3D_(AB124852.1) cccaaacatg gtaatggcgg atgtgctctt g...aggctg gtttccgtca

TaBx3A_(AB042628.1) cccaaatatg gtaatggcgg atgcgctctt g...aggctg gtttccgtca

ZmBx3_(X81829.1) cccgaatatg gccatggcgg acgtgctcct c...cggctg gtgtccgtca

HlBx3_(AY462228.1) cccaaatatg atcatggcgg atgtgctctt g...aggctg gtttccgtca

ScBx4_(KF636826) G...AGGTTG GCAAACCTGG ATGTGTTCCT C...AGGGTG GTCTGCTCCA

ScBx4_Picasso_(HG380518.1) G...AGGTTG GCAAACCTGG ATGTGTTCCT C...AGGGTG GTCTGCTCCA

HlBx4_(AY462229.1) a...aggttg gcaaacctgg atgtgttcct c...agggtg gtttgctcca

TaBx4A_(AB124854.1) a...aggttg gcaaacctgg atgtgttcct c...agggtg gtttgctcca

TaBx4D_(AB042627.1) t...aggttg gcaaacctgg acgtgttcct c...agggtg gtttgctcca

TaBx4B_(AB124855.1) g...aggttg gcaaacctgg atgtgttcct c...agggtg gtttgctcca

ZmBx4_(X81827.1) g...aagctg gcggacgtgg acctgttcct c...cggatc atctgcgcca

ScBx5_(KF636825) T...AGGTGG CCACTGACGG AGGTGCTCTT C...AGGCTG GTCTGCTGGA

TaBx5D_(AB124857.1) t...aggtgg ccactgacgg aggtgctctt c...aggctg gtctgctgga

TaBx5A_(AB042629.1) t...aggtgg ccattgacgg aggtgctctt c...aggctg gtctgctgga

HlBx5_(AY462230.1) t...aggtgg ccactgacgg aggtgctctt c...aggctg gtctgctgga

TaBx5B_(AB124856.1) t...aggtgg ccactgacgg aggtgctctt c...aggctg gtctgctgga

ZmBx5_(X81830.1) g...cggtgg ccgctgacgg acctgctctt c...aggctc gtgtgctgga

ScBx5_Picasso_(HG380519.1) T...AGGTGG CCACTGACGG AGGTGCTCTT CCTCAGGCTG GTCTGCTGGA

851 900

ScBx2_L318_(KF620524) AGGCCCGACG GGTGAGGAAG CGGTGGGACC TGCTCCTCGA CAAGCTGATT

ScBx2_(JX442061.1) aggcccgacg ggtgaggaag cggtgggacc tgctcctcga caagctgatt

ScBx2_Picasso_(HG380516.1) AGGCCCGACG GGTGAGGAAG CGGTGGGACC TGCTCCTCGA CAAGCTGATT

ZmBx2_(X81831.1) agaccaggag agtcaggaag cgctgggacg acctcctcga caagctcatc

HlBx2_(AY462227.1) aggcccgacg ggtgaggaag cggtgggacc tgctcctcga caagctaatt

TaBx2A_(AB042630.1) aggcccgacg ggtgaggaag cggtgggacc tgctcctcga caagctaatt

TaBx2B_(AB042631.1) aggcccgacg ggtgaggaag cggtgggacc tgctcctcga caagctaatt

TaBx2D_(AB124851.1) aggcccgacg ggtgaggaag cggtgggacc tgctcctcga caagctaatt

ScBx3_(KF636827) AGGCCCAGCG ACTCAACAAG AGGTGGGACG AATTGTTCAA CGAGATCATT

ScBx3_Picasso_(HG380517.1) AGGCCCAGCG ACTCAACAAG AGGTGGGACG AATTGTTCAA CGAGATCATT

TaBx3B_(AB124853.1) aggctcaacg actcaacaag aggtgggacg acttgttcaa cgagatcatt

TaBx3D_(AB124852.1) aggctcggcg gctcaacaag aggtgggacg aattgttcaa cgagatcatt

TaBx3A_(AB042628.1) aggctcagcg actcaacaag aggtgggacg acttgttcaa cgagatcatc

ZmBx3_(X81829.1) aggcccggcg gctcaaccag aggtggaacg acgtcttcga cgagctcatc

HlBx3_(AY462228.1) aggctcagcg actcaacaag aggtgggacg acctgttcaa cgagatcatt

ScBx4_(KF636826) AGGCAATGGG AGTCAGCAAG AGGTGGGACA ACCTGTTTAA CGAGCTCATC

ScBx4_Picasso_(HG380518.1) AGGCAATGGG AGTCAGCAAG AGGTGGGACA ACCTGTTTAA CGAGCTCATC

HlBx4_(AY462229.1) aggcaatggg agtcagcaag aggtgggaca acctgtttaa cgagctcatc

TaBx4A_(AB124854.1) aggcaatggg agtcagcaag aggtgggaca acctgtttaa cgagctcatc

TaBx4D_(AB042627.1) aggcaatggg agtcagcaag aggtgggaca acctgtttaa cgagctcatc

TaBx4B_(AB124855.1) aggcaatggg agtcagcaaa aggtgggaca acctgtttaa cgagctcatc

ZmBx4_(X81827.1) aggccaaggg cgtcagcaag cggtgggaca gcctcttcaa cgagctgctg

ScBx5_(KF636825) AGGTTCAGCG ACACCTCAAC AAGTGGGACG CCTTGCTGGA AGAGGTCATC

TaBx5D_(AB124857.1) aggttcagcg acacctcaac aagtgggacg ccttgctgga agaggtcatc

TaBx5A_(AB042629.1) aggttcagcg acacctcaac aagtgggacg ccttgctgga agaggtcatc

HlBx5_(AY462230.1) aggttcagcg gcacctcaac aaatgggacg ccttgctgga agaggtcatc

TaBx5B_(AB124856.1) aggttcagcg acacctcaac aagtgggacg ccttgctgga agaggtcatc

ZmBx5_(X81830.1) aggtcacgcg ccacctcaag cgctgggacg ccctgctgga ggaggtgatc

ScBx5_Picasso_(HG380519.1) AGGTTCAGCG ACACCTCAAC AAGTGGGACG CCTTGCTGGA AGAGGTCATC

901 950

ScBx2_L318_(KF620524) GACGACCATG CAGCAAGGAT GGTAAGCCGT GAGGATGAGG CCCAAGTGGA

ScBx2_(JX442061.1) gacgaccatg cagcaaggat ggtaagccgt gaggatgagg cccaggcgga

ScBx2_Picasso_(HG380516.1) GACGACCATG CAGCAAGGAT GGTAAGCCGT GAGGATGAGG CCCAGGTGGA

ZmBx2_(X81831.1) gacgaccatg cggccaggat ggcaacccat caggacgag. ..........

HlBx2_(AY462227.1) gacgaccatg cagcaaggat ggtaagccgt gaggatgagg cccaggggga

TaBx2A_(AB042630.1) gacgaccatg cagcaaggat ggtaagccgt gaggatgagg cccagccggc

TaBx2B_(AB042631.1) gacgaccatg cagcaaggat ggtaagccgt gagggtgagg cccagccgga

TaBx2D_(AB124851.1) gacgaccatg cagcaaggat ggtaagccgt gaggatgagg cccagccgga

ScBx3_(KF636827) GAAGAACACC TACACCCCAG CAAACCATCA TCT...GGCG AG........

ScBx3_Picasso_(HG380517.1) GAAGAACACC TACACCCCAG CAAACCATCA TCT...GGCG AG........

TaBx3B_(AB124853.1) gaggaacacc tgcaccccag caaaccatca tcc...ggcg ag........

TaBx3D_(AB124852.1) gaagaacacc tacaccccag caaaccatca tct...ggcg ag........

TaBx3A_(AB042628.1) gaggaacacc tacaccccag caaaccatca tct...ggcg ag........

ZmBx3_(X81829.1) caggaacacg tgcag...ag caggccctcc ggcgagagcg ag........

HlBx3_(AY462228.1) gaggaacacc tacaccccag caaaccatca tct...ggcg ag........

ScBx4_(KF636826) GCCGAATACG AA......GG CGGCAAGGAA ...GAT.... ..........

ScBx4_Picasso_(HG380518.1) GCCGAATACG AA......GG CGGCAAGGAA ...GAT.... ..........

HlBx4_(AY462229.1) gccgaatacg aa......gg cggtaaggaa ...gac.... ..........

TaBx4A_(AB124854.1) gccgaatacg aa......ca cggcaaggaa ...gat.... ..........

TaBx4D_(AB042627.1) gccgagtacg aa......ca cggcaaggaa ...gat.... ..........

TaBx4B_(AB124855.1) gccgaatatg aa......ca cggcaaggaa ...gat.... ..........

ZmBx4_(X81827.1) tccgagtacg cgctctccgg cggcaagcag ggcgaccac. ..........

ScBx5_(KF636825) AAGGAGCACA TGAACTTGAA GCAG...... ...GATAAT. ..........

TaBx5D_(AB124857.1) aaggagcaca taaacttgaa gcaa...... ...gataat. ..........

TaBx5A_(AB042629.1) aaggaacaca taaacttgaa gcaa...... ...gataat. ..........

HlBx5_(AY462230.1) aaggagcaca taaacttgaa gcaa...... ...gacaat. ..........

TaBx5B_(AB124856.1) aaggagcaca tcaacttgaa gcaa...... ...gacaat. ..........

ZmBx5_(X81830.1) cacgagcacg tggagatgag gaagctgtcc ggcgacaagg ag........

ScBx5_Picasso_(HG380519.1) AAGGAGCACA TGAACTTGAA GCAG...... ...GATAAT. ..........

951 1000

ScBx2_L318_(KF620524) GCAAGAAGAA GACAAAGACT TCATCGACGT CTCGCTATCT CTTCAGCAGG

ScBx2_(JX442061.1) gcaagaagaa gacaaagact tcatcgacgt ctcgctatct cttcagcagg

ScBx2_Picasso_(HG380516.1) GCAAGAAGAA GACAAAGACT TCATCGACGT CTCGCTATCT CTTCAGCAGG

ZmBx2_(X81831.1) ....gacgac gataaggact tcatatacgt attgctgtct cttcagaagg

HlBx2_(AY462227.1) gcaagaagaa gacaaagact tcatcgatgt ctccctatct cttcagcagg

TaBx2A_(AB042630.1) gcaagaggaa gacaaagact tcatcgacgt atccctatct cttcagcagg

TaBx2B_(AB042631.1) gcaagaagaa gacaaagact tcatcgacgt atccctatct cttcagcagg

TaBx2D_(AB124851.1) gcaagaagaa gacaaagact tcatcgacgt atccctctct cttcagcagg

ScBx3_(KF636827) ....CAGCAA GCAGCAGATT TCATAGATCT TCTGCTCTCT CTCAAGGAAG

ScBx3_Picasso_(HG380517.1) ....CAGCAA GCAGCAGATT TCATAGATCT TCTGCTCTCT CTCAAGGAAG

TaBx3B_(AB124853.1) ....cagcaa gcagcagatt tcatagatct tctgctctct ctcaaggaag

TaBx3D_(AB124852.1) ....cagcaa gcggtagatt tcatagatct tctgctctct ctcaaggaag

TaBx3A_(AB042628.1) ....cagcaa gcagcagatt tcatagatct tctgctctct ctcaaggaag

ZmBx3_(X81829.1) ....gagagc gaggcggact tcatacacgt tctgctctcc atacagcaag

HlBx3_(AY462228.1) ....cagcaa gcagcagatt tcatagatct tctgctctct ctcaaggaag

ScBx4_(KF636826) .......AAC GCGGAGGACT TCGTACATCT TTTGCTTTCT CTCAAGAAAG

ScBx4_Picasso_(HG380518.1) .......AAC GCGGAGGACT TCGTACATCT TTTGCTTTCT CTCAAGAAAG

HlBx4_(AY462229.1) .......aac gcggaggact tcgtacatct tttgctttct ctgaagaaag

TaBx4A_(AB124854.1) .......aac gcggaggact tcgtacatct tttgctttcg ctgaagaaag

TaBx4D_(AB042627.1) .......aat gcggaagact ttgtacatct tttgctttct ctaaagaaag

TaBx4B_(AB124855.1) .......aac gcggaggact tcgtacatct tttgctttct ctgaagaaag

ZmBx4_(X81827.1) .......aac tctgaagact tcgtgcacct cttgctgtcg ctgcagaaag

ScBx5_(KF636825) .......... TCTGCTGACT TCATCCATGT TTTCCTCTCT CTACAGCAAG

TaBx5D_(AB124857.1) .......... tctgccgact tcatccatgt tttcctctct ctacagcaag

TaBx5A_(AB042629.1) .......... tctgccgact tcatccatgt tttcctctct ctacagcaag

HlBx5_(AY462230.1) .......... tctgccgact tcatccatgt tttcctctcg ctacagcaag

TaBx5B_(AB124856.1) .......... tctgccgact tcatccatgt tttcctctct ctacagcaag

ZmBx5_(X81830.1) ....aaggag tcggacgact tcatcgacat cttcctctcc agatacgagg

ScBx5_Picasso_(HG380519.1) .......... TCTGCTGACT TCATCCATGT TTTCCTCTCT CTACAGCAAG

1001 1050

ScBx2_L318_(KF620524) AGTATGGTCT CACCAGGGAC CATATCAAGG CCATCTTGAT AGACATGTTT

ScBx2_(JX442061.1) agtatggtct caccagggac catatcaagg ccatcttgat agacatgttt

ScBx2_Picasso_(HG380516.1) AGTATGGTCT CACCAGGGAC CATATCAAGG CCATCTTGAT AGACATGTTT

ZmBx2_(X81831.1) agtatggcct caccagagac catatcaagg ctatcttgat agacatgttc

HlBx2_(AY462227.1) agtatggtct caccagggac catatcaagg ccatcttgat agacatgttt

TaBx2A_(AB042630.1) agtatggtct caccagggac catatcaagg ccatcttgat agacatgttt

TaBx2B_(AB042631.1) agtatggtct caccagggac catatcaagg ccatcttgat agacatgttt

TaBx2D_(AB124851.1) agtatggtct caccaggcac catatcaagg ccatcttgat agacatgttt

ScBx3_(KF636827) AGTACGGTCT CACTACGGAT AACATCAAGG CCATCTTGGT GGACATGTTT

ScBx3_Picasso_(HG380517.1) AGTACGGTCT CACTACGGAT AACATCAAGG CCATCTTGGT GGACATGTTT

TaBx3B_(AB124853.1) agtacggtct cactacggat aacatcaagg ccatcttggt ggacatgttt

TaBx3D_(AB124852.1) agtacggtct cactacagat aacatcaagg ccatcttggt ggacatgttt

TaBx3A_(AB042628.1) agtacggtct cactacggat aacatcaagg ccattttggt ggacatgttt

ZmBx3_(X81829.1) agtacggcct cactacagac aatctcaagg ccatcttggt ggacatgttc

HlBx3_(AY462228.1) agtacggtct cactacggat aacatcaagg ccatcttggt ggacatgttt

ScBx4_(KF636826) AGTACGGCCT CTCCACGGAT AACGTCAAGG CCATCTTAGT GAACATGTTT

ScBx4_Picasso_(HG380518.1) AGTACGGCCT CTCCACGGAT AACGTCAAGG CCATCTTAGT GAACATGTTT

HlBx4_(AY462229.1) agtacaatct ctccacggat aatgtcaagg ccatcttggt gaacatgttt

TaBx4A_(AB124854.1) agtacggtct ctccacggat aacgtcaagg ccatcttggt gaacatgttt

TaBx4D_(AB042627.1) agtacggtct gtccacagat aacgtcaagg ccatcttggt gaacatgttt

TaBx4B_(AB124855.1) agtacggtct ctccacggat aacgtcaagg ccatcttggt gaacatgttt

ZmBx4_(X81827.1) actacggtct caccacggat aacatcaagg gcatacttgt gaacatgttc

ScBx5_(KF636825) AGTACGGTCT CACCGACGAT AACGTCAAGT CCCTCTTGAT GAACATATTT

TaBx5D_(AB124857.1) agtacggtct caccgacgat aatgtcaagt ccctcttgat gaacatattt

TaBx5A_(AB042629.1) agtacggtct caccgacgat aacgtcaagt ccctcttgat gaacatattt

HlBx5_(AY462230.1) agtacggtct caccgacgat aatgtcaagt ccctcttgat gaacatattc

TaBx5B_(AB124856.1) agtacggtct caccgacgat aatgtcaagt ccctcttgat gaacatattt

ZmBx5_(X81830.1) agtacggctt caccatggat aacgtcaagt ccctcctcat gaacgtgttc

ScBx5_Picasso_(HG380519.1) AGTACGGTCT CACCGACGAT AACGTCAAGT CCCTCTTGAT GAACATATTT

1051 1100

ScBx2_L318_(KF620524) GAGGCCGGCA CAGACACCTC GTATATGACG CTGGAATTTG CCATGGCGGA

ScBx2_(JX442061.1) gaggccggca cagacacctc gtatatgacg ctggaatttg ccatggcgga

ScBx2_Picasso_(HG380516.1) GAGGCCGGCA CAGACACCTC GTATATGACG CTGGAATTTG CCATGGCGGA

ZmBx2_(X81831.1) gaggccggaa ccgacacctc gtacatgacg ttggagttcg ccatgacgga

HlBx2_(AY462227.1) gaggccggca cggatacctc gtatatgacg ctggagtttg ccatggcgga

TaBx2A_(AB042630.1) gaggccggca cggacacctc gtatatgacg ctggagtttg ccatggcgga

TaBx2B_(AB042631.1) gaggccggca cggacacctc gtatatgacg ctggagtttg ccatggcgga

TaBx2D_(AB124851.1) gaggccggca cggacacctc gtatatgacg ctggagtttg ccatggcgga

ScBx3_(KF636827) GAGGCAGGCA TAGAAACATC CTATCTGACG TTGGAATACG GCATGGCTGA

ScBx3_Picasso_(HG380517.1) GAGGCAGGCA TAGAAACATC CTATCTGACG TTGGAATACG GCATGGCTGA

TaBx3B_(AB124853.1) gaggctggca tagaaacatc ctatctgacg ttggagtacg gcatggctga

TaBx3D_(AB124852.1) gaggcaggca tagaaacatc ttatctgacg ttggagtacg gcatggctga

TaBx3A_(AB042628.1) gaggcaggca tagaaacatc ttatctgacg ttggaatacg gcatggctga

ZmBx3_(X81829.1) gaggcaggta tagagacatc ttacctgacg ctagagtacg gcatggccga

HlBx3_(AY462228.1) gaggcaggca tagaaacatc ctatctgacg ttggaatacg gcatggctga

ScBx4_(KF636826) GAGGCAGCTA TAGAAACATC ATTCCTGGTG CTGGAATACT CCATGGCCGA

ScBx4_Picasso_(HG380518.1) GAGGCAGCTA TAGAAACATC ATTCCTGGTG CTGGAATACT CCATGGCCGA

HlBx4_(AY462229.1) gaggcggcta tagaaacatc attcctggtg ctggaatact ccatggccga

TaBx4A_(AB124854.1) gaggcagcta tagaaacatc attcctggtg ctggaatact ccatggccga

TaBx4D_(AB042627.1) gaggcagcta tagaaacatc attcctggtg ctggaatact ccatggccga

TaBx4B_(AB124855.1) gaggcagcta tagaaacatc attcctggtg ctggaatact ccatggccga

ZmBx4_(X81827.1) gaagcagcca tcgagacatc attcctggtg ctggagtact ccatgagcga

ScBx5_(KF636825) GAGGCAGCCA TAGAAACATC GTATCTGGTG TTGGAATACG CCATGGCCGA

TaBx5D_(AB124857.1) gaggcagcta tagaaacatc gtatctggtg ctggaatacg ccatggccga

TaBx5A_(AB042629.1) gaggcagcca tagaaacatc gtatttggtg ctggaatacg ccatggctga

HlBx5_(AY462230.1) gaggcagcta tagaaacatc gtatctggtg ctggaatacg ccatggccga

TaBx5B_(AB124856.1) gaggcagcca tagaaacatc atatctggtg ctggaatacg ccatggccga

ZmBx5_(X81830.1) gaggcagcca tcgagacctc atatctggtg ctggagtccg ccatggccga

ScBx5_Picasso_(HG380519.1) GAGGCAGCCA TAGAAACATC GTATCTGGTG TTGGAATACG CCATGGCCGA

1101 1150

ScBx2_L318_(KF620524) GCTCATACGG AAGCCACACC TTATGAAGAA GCTGCAGGAG GAGGTTAGG.

ScBx2_(JX442061.1) gctcatacgg aagccacacc ttatgaagaa gctgcaggag gaggttagg.

ScBx2_Picasso_(HG380516.1) GCTCATACGG AAGCCACACC TTATGAAGAA GCTGCAGGAG GAGGTTAGG.

ZmBx2_(X81831.1) gctcatacgg aagccacacc tgatgaagaa gctgcaggaa gaagtgcgg.

HlBx2_(AY462227.1) gctcatacgg aagccacacc ttctgaacaa gctgcaggaa gaggtaagg.

TaBx2A_(AB042630.1) gctcatacgg aagccacacc ttatgaagaa gctgcaggaa gaggtaagg.

TaBx2B_(AB042631.1) gctcatacgg aagccacacc ttatgaagaa gctgcaggaa gaggtaagg.

TaBx2D_(AB124851.1) gctcatacgg aagccacacc ttatgaagaa gctgcaggaa gaggtaagg.

ScBx3_(KF636827) GCTCATGAAC AACAGACACA TTCTGAAAAA ATTACAGGAG GAGGTAAGA.

ScBx3_Picasso_(HG380517.1) GCTCATGAAC AACAGACACA TTCTGAAAAA ATTACAGGAG GAGGTAAGA.

TaBx3B_(AB124853.1) gctcatgaac aacagacaca ttctgaagaa attacaggag gaggtaaga.

TaBx3D_(AB124852.1) gctcatgaac aacagacaca ttctgaaaaa attacaggag gaggtaaga.

TaBx3A_(AB042628.1) gctcatgaac aacagacaca ttctgacaaa attacaggag gaggtaaga.

ZmBx3_(X81829.1) gctcatcaac aacaggcacg tcatggagaa gctacagacg gaggtgagga

HlBx3_(AY462228.1) gctcatgaac aacagacaca ttctgacaaa attacaggag gaggtaaga.

ScBx4_(KF636826) GCTCATCAAC AACAGGCACG TCATGGCGAA GGTGCAAAAG GAGGTAAGG.

ScBx4_Picasso_(HG380518.1) GCTCATCAAC AACAGGCACG TCATGGCGAA GGTGCAAAAG GAGGTAAGG.

HlBx4_(AY462229.1) gctcatcaac aacaggcacg tcatgaccaa agtgcagaag gaggtaagg.

TaBx4A_(AB124854.1) gctcatcaac aacaggcacg tcatggccaa agtgcaaaag gaggtaagg.

TaBx4D_(AB042627.1) gctcatcaac aacaggcacg tcatggccaa agtgcagaag gaggtaagg.

TaBx4B_(AB124855.1) gctcatcaac aacaggcacg tcatggccaa agtgcaaaag gaggtaagg.

ZmBx4_(X81827.1) gctgatgaac aaccgccacg tcctggccaa gctccagaag gaggtgagg.

ScBx5_(KF636825) ACTTATCAAC AACAGACATG TTATGAAGAA ACTACAAACC GAGGTAAGG.

TaBx5D_(AB124857.1) gcttataaac aacagacatg ttatgaagaa actacaaacc gaggtaagg.

TaBx5A_(AB042629.1) gcttatcaac aacagacatg ttatgaagaa acttcaaacc gaggtaagg.

HlBx5_(AY462230.1) gcttatcaac aacagacatg ttatgaagaa actacaaacc gaggtaagg.

TaBx5B_(AB124856.1) gcttatcaac aacagacatg ttatgaagaa actacaaacc gaggtaagg.

ZmBx5_(X81830.1) gctcatgaac cacaggcgcg tcatgaagaa gctgcaagcg gaggtacgg.

ScBx5_Picasso_(HG380519.1) ACTTATCAAC AACAGACATG TTATGAAGAA ACTACAAACC GAGGTAAGG.

1151 1200

ScBx2_L318_(KF620524) .....AGGAA TGTACCCAAC GGGCAA.... ..GAGATGGT CGCCGAAGAC

ScBx2_(JX442061.1) .....aggaa tgtacccaac gggcaa.... ..gagatggt cgccgaagac

ScBx2_Picasso_(HG380516.1) .....AGGAA TGTACCCAAC GGGCAA.... ..GAGATGGT CGCCGAAGAC

ZmBx2_(X81831.1) .....cggaa cgtgccggcg gggcag.... ..gagatggt caccgaggac

HlBx2_(AY462227.1) .....aggaa tgtacccaac gggcaa.... ..gagatggt cgccgaagac

TaBx2A_(AB042630.1) .....aggaa tgtaccaaac gggcaa.... ..gagatggt cgccgaagac

TaBx2B_(AB042631.1) .....aggaa tgtacccaac gggcaa.... ..gagatggc cgccgaagac

TaBx2D_(AB124851.1) .....aggaa tgtaaccaac gggcaa.... ..gagatggt cgccgaagac

ScBx3_(KF636827) .......... .TCC...CAA GGCAAAAAGC TAGACATGAT AACAGAGGAG

ScBx3_Picasso_(HG380517.1) .......... .TCC...CAA GGCAAAAAGC TAGACATGAT AACAGAGGAG

TaBx3B_(AB124853.1) .......... .tcc...caa ggcaaaaaat tagacatgat aacagaggag

TaBx3D_(AB124852.1) .......... .tcc...caa ggcaaaaagt tagacatgat aacggaggag

TaBx3A_(AB042628.1) .......... .tcc...caa ggcaaaaagt tagacatgat aacggaggag

ZmBx3_(X81829.1) cgacgatggg ctcgccggac ggcaagaagc tggacatgtt agcggaggag

HlBx3_(AY462228.1) .......... .tcc...caa ggcaaaaagt tagacatgat aacggaggag

ScBx4_(KF636826) .....GAGTC CACACCCAAC GGCGAAAAGC TGGACCTGAT AATGGAGGAG

ScBx4_Picasso_(HG380518.1) .....GAGTC CACACCCAAC GGCGAAAAGC TGGACCTGAT AATGGAGGAG

HlBx4_(AY462229.1) .....gagtc cacacccgag ggcggaaagc tggacctgat aatggaggag

TaBx4A_(AB124854.1) .....gagtc cacacccggc ggcgaaaagc tggacctgat aatggaggag

TaBx4D_(AB042627.1) .....gagtc cacacccaag ggcgaaaagc tggacctgat aatggaggag

TaBx4B_(AB124855.1) .....gagtc cacaccccac ggcgaaaagc tggacctgat aatggaggag

ZmBx4_(X81827.1) .....acggc gacgcccgac ggccgc.... .....atggt gatggaggag

ScBx5_(KF636825) ..ACATTTGC ATCATCCAAG GGCAAAAAGT TGGACATGAT TACGGAGGAG

TaBx5D_(AB124857.1) ..acgtttgc atcgtccaag ggcaaaaagt tggacatgat tacggaggag

TaBx5A_(AB042629.1) ..acgtttgc atcatccaag ggcaaaaggt tggacatgat tacggaggag

HlBx5_(AY462230.1) ..acgtttgc atcatccaag ggcaaaaagt tggacatgat tacggaggag

TaBx5B_(AB124856.1) ..acgtttgc atcgtccaag ggaaaaaagt tggacatgat tacggaggag

ZmBx5_(X81830.1) ..gcgtacgg agcggagaag ......aagc tggacatgat cagggaggac

ScBx5_Picasso_(HG380519.1) ..ACATTTGC ATCATCCAAG GGCAAAAAGT TGGACATGAT TACGGAGGAG

1201 1250

ScBx2_L318_(KF620524) GATCTCCCCA ACATGACCTA CCTAAAGGCT GTCATCAAGG AGACACTCCG

ScBx2_(JX442061.1) gatctcccca acatgaccta cctaaaggct gtcatcaagg agacactccg

ScBx2_Picasso_(HG380516.1) GATCTCCCCA ACATGACCTA CCTAAAGGCT GTCATCAAGG AGACACTCCG

ZmBx2_(X81831.1) aacctccccg gcatgaccga cctcaaggcc gtcatcaagg agacgctccg

HlBx2_(AY462227.1) gatctcccca acatgaccta cctcaaagct gttatcaagg aaacactccg

TaBx2A_(AB042630.1) gatctcccca acatgaccta cctcaaggct gtcatcaagg agacactccg

TaBx2B_(AB042631.1) gatctcccca acatgaccta cctcaaggct gtcatcaagg agacactccg

TaBx2D_(AB124851.1) gatctcccca acatgaccta cctcaaggct gtcatcaagg agacactccg

ScBx3_(KF636827) GACCTTAGCA GCATGGCCTA CCTAAGGGCA ACCATCAAGG AGACGCTGCG

ScBx3_Picasso_(HG380517.1) GACCTTAGCA GCATGGCCTA CCTAAGGGCA ACCATCAAGG AGACGCTGCG

TaBx3B_(AB124853.1) gaccttagca gcatggccta cctaagggca accatcaagg agacgctgcg

TaBx3D_(AB124852.1) gaccttggca gcatggccta cctaagggca accatcaagg agacgctgcg

TaBx3A_(AB042628.1) gaccttagca gcatggccta cctaagggca accatcaagg agacgctgcg

ZmBx3_(X81829.1) gacctcggca gcatgcccta cctcaaggcc accatcaagg agacgctgcg

HlBx3_(AY462228.1) gatgttagca gcatggccta cctaagggca accatcaagg agacgtcgcg

ScBx4_(KF636826) GACCTCAGCC GCATGCCGTA CCTCAAGGCG ACCATCAAAG AGGCGATGCG

ScBx4_Picasso_(HG380518.1) GACCTCAGCC GCATGCCGTA CCTCAAGGCG ACCATCAAAG AGGCGATGCG

HlBx4_(AY462229.1) gacctcagcc gcatgccgta cctcaaggcg accatcaaag aggcgatgcg

TaBx4A_(AB124854.1) gacctcagcg gcatgcccta cctcaaggcg accatcaagg aggcgatgcg

TaBx4D_(AB042627.1) gacctcagcc gcatgccgta cctcaaggcg accatcaagg aggcaatgcg

TaBx4B_(AB124855.1) gacctcagcc gcatgccgta cctcaaggcg accatcaagg aggcgatgcg

ZmBx4_(X81827.1) gacctgagcc ggatgccata cctgaaggcc accatcaagg agtccatgcg

ScBx5_(KF636825) GACCTCAGCA GCCTGCCCTA CCTAAAGGCA ACCATGAAAG AGGCCCTCCG

TaBx5D_(AB124857.1) gacctcagca gcctgcccta cctaaaggca actatgaaag aggccctccg

TaBx5A_(AB042629.1) gacctcagca gcctgcccta cctaaaggca accatgaaag aggccctgcg

HlBx5_(AY462230.1) gacctcagca gcttgcccta cctaaaggca accatgaaag aggccctgcg

TaBx5B_(AB124856.1) gacctcagca gcctgccgta cctaaaggca accatgaaag aggcgctgcg

ZmBx5_(X81830.1) gacctgagca gcctgccgta cctaaaggcg tccatgaagg aagcgctgcg

ScBx5_Picasso_(HG380519.1) GACCTCAGCA GCCTGCCCTA CCTAAAGGCA ACCATGAAAG AGGCCCTCCG

1251 1300

ScBx2_L318_(KF620524) GCTGCACCCA CCGGTACCTC TCATGATTCC ACACTTCTCC CTAGATGCGT

ScBx2_(JX442061.1) gctgcaccca ccggtacctc tcatgattcc acacttctcc ctagatgcgt

ScBx2_Picasso_(HG380516.1) GCTGCACCCA CCGGTACCTC TCATGATTCC ACACTTCTCC CTAGATGCGT

ZmBx2_(X81831.1) cctgcacccg ccggtgccgc tcctcctccc gcactactcc ctggacgcct

HlBx2_(AY462227.1) gctgcaccca ccggtacctc tcatgattcc acacttctcc cttgatgcct

TaBx2A_(AB042630.1) gctgcacccg ccggtacctc tcatgattcc acacttctcc ctagacgcct

TaBx2B_(AB042631.1) gctgcatccg ccggtacctc tcatgattcc acacttctcc ctagatgcct

TaBx2D_(AB124851.1) actgcatccg ccggtacctc tcatgattcc acacttctcc ctagatgcct

ScBx3_(KF636827) CTTGCATCCA CCGGCACCCT TCCTCCTCCC GCACTTCTCC ACCGCTGACT

ScBx3_Picasso_(HG380517.1) CTTGCATCCA CCGGCACCCT TCCTCCTCCC GCACTTCTCC ACCGCTGACT

TaBx3B_(AB124853.1) catgcaccca ccagcgccct tcctcctccc gcacttctcc accgctgact

TaBx3D_(AB124852.1) cttgcaccca ccagcgccct tcctcctccc acacttctcc accgctgact

TaBx3A_(AB042628.1) catgcaccca ccagcaccct tcctcctccc acacttctcc accgctgact

ZmBx3_(X81829.1) cctgcacccg cccgcgccgt tcctcctccc gcactactcc accgccgact

HlBx3_(AY462228.1) cttgcaccca ccggcgccct tcctcctccc acacttctcc accgctgact

ScBx4_(KF636826) CATACACCCA CCGGCGCCCT TCCTGCTCCC ACACTTCTCC ACCAACGACT

ScBx4_Picasso_(HG380518.1) CATACACCCA CCGGCGCCCT TCCTGCTCCC ACACTTCTCC ACCAACGACT

HlBx4_(AY462229.1) cgtacacccg ccggcgcctt tcctgctccc gcacttctcc accaacgact

TaBx4A_(AB124854.1) catacacccg ccggcgccct tcctgctccc acacttctcc accaacgact

TaBx4D_(AB042627.1) catacacccg ccggcaccct tcctgctccc acacttctcc accaacgact

TaBx4B_(AB124855.1) catacacccg ccggcgccct tcctgctccc acacttctcc accaatgact

ZmBx4_(X81827.1) catccacccg ccggcgccgt tcctcctgcc gcacttctcc acccacgact

ScBx5_(KF636825) CTTGCACCCA CCAGGGCCTT TACTCCTCCC ACACTACTCC ACCGCTGACT

TaBx5D_(AB124857.1) cttgcaccca ccagggcctt tactcctccc acactactcc actgctgact

TaBx5A_(AB042629.1) cttgcaccca ccagggcctt tactcctccc acactactcc accgctgact

HlBx5_(AY462230.1) cttgcaccca ccagggcctt tactcctccc acactactcc accgctgatt

TaBx5B_(AB124856.1) cttgcaccca ccagggcctt tactcctccc acactactcc actgctgatt

ZmBx5_(X81830.1) gctgcaccca ccggggcccc tgctactgcc gcactactcc accgccgact

ScBx5_Picasso_(HG380519.1) CTTGCACCCA CCAGGGCCTT TACTCCTCCC ACACTACTCC ACCGCTGACT

1301 1350

ScBx2_L318_(KF620524) GTACCGTTGA TGGTTACACG ATACCAGCAA ACACTCGTGT CGTTGTAAAC

ScBx2_(JX442061.1) gcaccgttga tggttacacg ataccagcaa acactcgtgt cgttgtaaac

ScBx2_Picasso_(HG380516.1) GTACCGTTGA TGGTTACACG ATACCAGCAA ACACTCGTGT CGTTGTAAAC

ZmBx2_(X81831.1) gcgaggtcgc cggctacacc atcccggcca acacccgcgt cgtcgtcaac

HlBx2_(AY462227.1) gcacggttga cggctacacg atcccagcaa acactcgtgt cgttatcaac

TaBx2A_(AB042630.1) gcaccgttga tggctacacg atcccagcaa acactcgtgt cgttatcaac

TaBx2B_(AB042631.1) gcaccgttga tggctacacg atcccagcaa acactcgtgt cgttatcaac

TaBx2D_(AB124851.1) gcaccgttga tggctacacg atcccagcaa acactcgtgt cgttatcaac

ScBx3_(KF636827) GCAAGATCGA CGGATACTTG ATACCCTCCA ACACACGCGT CCTTGTGAAT

ScBx3_Picasso_(HG380517.1) GCAAGATCGA CGGATACTTG ATACCCTCCA ACACACGCGT CCTTGTGAAT

TaBx3B_(AB124853.1) gcaaggtcga cggatacttg atacccgcca acacacgtgt ccttgtgaat

TaBx3D_(AB124852.1) gcaagatcga cggatacttg ataccctcca acacacgcgt ccttgtgaat

TaBx3A_(AB042628.1) gcaagatcga cggatacttg atacccgcca acacacgtgt ccttgtgaat

ZmBx3_(X81829.1) ccgagatcga cggctacttc gtccccgccg ggacgcgcgt cctcgtccac

HlBx3_(AY462228.1) gcaacatcga cggatacgtg gtaccctcca acacacgtgt ccttgtgaat

ScBx4_(KF636826) GCGAGATCAA CGGATACACC ATTCCCGCGG GAACACGCGT CATTGTGAAT

ScBx4_Picasso_(HG380518.1) GCGAGATCAA CGGATACACC ATTCCCGCGG GAACACGCGT CATTGTGAAT

HlBx4_(AY462229.1) gcgaggtcaa cggatacacc attccggcag gaacacgcgt cattgtgaac

TaBx4A_(AB124854.1) gcgaggttaa tggatacacc attcctgcgg gaacacgtgt cattgtgaat

TaBx4D_(AB042627.1) gcgaggtcaa cggatacacc attcccgcgg ggacacgtgt cattgtgaat

TaBx4B_(AB124855.1) gcgagatcaa cggatacacg attcccgcgg gaacacgggt cattgtgaac

ZmBx4_(X81827.1) gcgagatcaa cggctacacc atccccgccg gcacgcgcgt catcgtcaac

ScBx5_(KF636825) GCAACATCGA CGGATACGAC ATACCCGCCA AAACACGTAT CCTTGTGAAC

TaBx5D_(AB124857.1) gcaacattga cggatacgac atacccgcca aaacacgtat ccttgtgaac

TaBx5A_(AB042629.1) gcaacatcga cggatacgac atacccgcca aaacacgtat ccttgtgaac

HlBx5_(AY462230.1) gcaacatcga cggatacgac atacccgcca aaacacgtat ccttgtgaac

TaBx5B_(AB124856.1) gcagcatcga cggatatgac ataccggcca aaacacgtat ccttgtgaat

ZmBx5_(X81830.1) gccagatcga cgggtatcac atccccgcca acccgcgcgt cctcgtgaac

ScBx5_Picasso_(HG380519.1) GCAACATCGA CGGATACGAC ATACCCGCCA AAACACGTAT CCTTGTGAAC

1351 1400

ScBx2_L318_(KF620524) GCCTGGGCAC TCGGCAGGCA CAGTGGCTAC TGGGAAAATG AAAATGAATT

ScBx2_(JX442061.1) gcctgggcac tcggcaggca cagtggctac tgggaaaatg aaaatgaatt

ScBx2_Picasso_(HG380516.1) GCCTGGGCAC TCGGCAGGCA CAGTGGCTAC TGGGAAAATG AAAATGAATT

ZmBx2_(X81831.1) gcctgggcgc tcggccgcca cagcggctac tgggagcgcg agaacgagtt

HlBx2_(AY462227.1) gcctgggcac tcggaaggca cagtagctac tgggaaaatg aaaacgaatt

TaBx2A_(AB042630.1) gcctgggcac tcggcaggca cagtagctac tgggaaaatg aaaatgaatt

TaBx2B_(AB042631.1) gcctgggcac tcggcaggca cagtggctac tgggaaaatg aaaatgaatt

TaBx2D_(AB124851.1) gcctgggcac tcggcaggca cagtggctac tgggaaaatg aaaatgaatt

ScBx3_(KF636827) GCCTGGGCCC TTGGGAGGGA TCCGTCGTCT TGGGAGAGGC CAGAGGATTT

ScBx3_Picasso_(HG380517.1) GCCTGGGCCC TTGGGAGGGA TCCGTCGTCT TGGGAGAGGC CAGAGGATTT

TaBx3B_(AB124853.1) gcctgggccc tagggaggga tccgtcgtct tgggagaggc cagatgattt

TaBx3D_(AB124852.1) gcttgggccc ttgggaggga tccatcgtct tgggagaggc cagaggattt

TaBx3A_(AB042628.1) gcttgggccc tagggaggga tccatcgtct tgggagaggc cagaggattt

ZmBx3_(X81829.1) gcctgggcgc tcgggaggga ccgcacgacg tgggagaagc cggaggagtt

HlBx3_(AY462228.1) gcctgggccc ttgggaggga tctatcgtct tgggagaggc cagatgattt

ScBx4_(KF636826) GCTTGGGCTC TTGCCAGAGA CCCATTGCAC TGGGAGAGAG CGGAGGAGTT

ScBx4_Picasso_(HG380518.1) GCTTGGGCTC TTGCCAGAGA CCCATTGCAC TGGGAGAGAG CGGAGGAGTT

HlBx4_(AY462229.1) gcttgggctc ttgccagaga cccgtcgcac tgggagagag cggaggagtt

TaBx4A_(AB124854.1) gcttgggctc ttgccagaga cccgtcgcac tgggagagag cggaggagtt

TaBx4D_(AB042627.1) gcttgggctc ttgccagaga cccgtcgcac tgggagagag cggaggagtt

TaBx4B_(AB124855.1) gcttgggctc ttgccagaga cccgtcgcac tgggagagag cggaggagtt

ZmBx4_(X81827.1) gcgtgggcgc tcgccaggga cccgacgtgc tgggacaagg ccgaggagtt

ScBx5_(KF636825) GGTTGGGCCA TCGGGAGAGA CCCAACAGCC TGGGAGAAAC CAGAGGATTT

TaBx5D_(AB124857.1) ggttgggcca ttggaagaga cccaacagct tgggagaggc cagaggattt

TaBx5A_(AB042629.1) ggttgggcca tcgggagaga cccaaaagct tgggagagac cagaggattt

HlBx5_(AY462230.1) ggttgggcca tcgggagaga cccaacagct tgggagagac aagaggattt

TaBx5B_(AB124856.1) ggttgggcca ttgggagaga cccaaaggct tgggagagac cggaggagtt

ZmBx5_(X81830.1) ggctgggcca tcggcagaga cccggcggtc tgggagaagc ccgaggagtt

ScBx5_Picasso_(HG380519.1) GGTTGGGCCA TCGGGAGAGA CCCAACAGCC TGGGAGAAAC CAGAGGATTT

1401 1450

ScBx2_L318_(KF620524) CCAACCTGAG AGATTTATGA ATGGA..... .GCCGGC... ...GTTGATC

ScBx2_(JX442061.1) ccaacctgag agatttatga atgga..... .gccggc... ...gttgatc

ScBx2_Picasso_(HG380516.1) CCAACCTGAG AGATTTATGA ATGGA..... .GCCGGC... ...GTTGATC

ZmBx2_(X81831.1) cgtcccggag aggttcctca gcggggacgt ggccggcggc ...gtcgacc

HlBx2_(AY462227.1) ccaacctgag agattcatga atgga..... .gccggc... ...gttgatc

TaBx2A_(AB042630.1) ccaacctgag agatttatga atgga..... .gccggc... ...gttgatc

TaBx2B_(AB042631.1) ccaacctgag agatttatga atgga..... .gccggc... ...gttgatt

TaBx2D_(AB124851.1) ccaacctgag agatttatga atgga..... .gccggc... ...gttgatc

ScBx3_(KF636827) CTGGCCTGAG AGGTTTCTGC AGGATCAAGC TGGCGATGTG GACACCCAGA

ScBx3_Picasso_(HG380517.1) CTGGCCTGAG AGGTTTCTGC AGGATCAAGC TGGCGATGTG GACACCCAGA

TaBx3B_(AB124853.1) ctggcctgag aggtttctgc aggatcaagc tggcgatgtg gacacccaga

TaBx3D_(AB124852.1) cttgcctgag aggtttctac aagatcaaga tggtgatgtt gacacccaaa

TaBx3A_(AB042628.1) ctggcctgag aggtttctgc aggatcaaga tggtgatgtg gacacccaaa

ZmBx3_(X81829.1) catgccggag cggttcgtgc aggaaccc.. .ggcgcggtg gacgtccaca

HlBx3_(AY462228.1) cttgcctgag aggtttctgc aagatcaagc tggcgatgtg gacacccaga

ScBx4_(KF636826) CTACCCAGAG AGGTTTCTCC AAGAAGGCCG TGACGCAGAG ...GTCGACA

ScBx4_Picasso_(HG380518.1) CTACCCAGAG AGGTTTCTCC AAGAAGGCCG TGATGCAGAG ...GTCGACA

HlBx4_(AY462229.1) ctacccagag aggtttctgc aagaaggccg tgatgcagag ...gtcgaca

TaBx4A_(AB124854.1) ctacccagag aggtttctcc aagaaggccg tgatgcagag ...gtcgaca

TaBx4D_(AB042627.1) ctacccggag aggtttctcc aagaaggccg tgatgcagag ...gtcgaca

TaBx4B_(AB124855.1) ctacccggag aggtttctcc aagaaggccg tgatgcagag ...gtcgaca

ZmBx4_(X81827.1) cttcccggag cgcttcctgg aacaaggccg cgacgccgag ...gtcgaca

ScBx5_(KF636825) CATGCCCGAG AGGTTCCTAC AAGATGGTCA GGAGAAATCT ...AGCAACC

TaBx5D_(AB124857.1) catgccggag aggttcctac aagatggcca ggagaaatct ...agcaacc

TaBx5A_(AB042629.1) catgccggag aggttcctac aagatggtca ggagaaatct ...agcaacc

HlBx5_(AY462230.1) catgcccgag aggttcctac aggaaggtca ggagaaatct ...agcaact

TaBx5B_(AB124856.1) catgcccgag aggttcctac aagatggtca ggagaaatct ...agcaacc

ZmBx5_(X81830.1) catgccggag aggttcatgc gggacggctg ggacaagtcc aacagctaca

ScBx5_Picasso_(HG380519.1) CATGCCCGAG AGGTTCCTAC AAGATGGTCA GGAGAAATCT ...AGCAACC

1451 1500

ScBx2_L318_(KF620524) TGAAGCCAAA TGAGTTCCAT TACTTGCCAT TTGGGTTTGG ACGAAGAATG

ScBx2_(JX442061.1) tgaagccaaa tgagttccat tacttgccat ttgggtttgg acgaagaatg

ScBx2_Picasso_(HG380516.1) TGAAGCCAAA TGAGTTCCAT TACTTGCCAT TTGGGTTTGG ACGAAGAATG

ZmBx2_(X81831.1) tcaagcccaa cgagttccag ttcctggcgt tcgggtccgg gcggaggatg

HlBx2_(AY462227.1) tcaagccaaa tgagttccat tacttgccat ttgggtttgg acgaagaatg

TaBx2A_(AB042630.1) tgaagccaaa tgagttccat tacttgccat ttgggtttgg acgtagaatg

TaBx2B_(AB042631.1) tgaagccaaa tgagttccat tacttgccat ttgggtttgg acgaagaatg

TaBx2D_(AB124851.1) tgaagccaaa tgagttccat tacttgccat ttgggtttgg acgaagaatg

ScBx3_(KF636827) TGAGGGGTAA AGATCTTAGG TTCCTGCCTT TTGGGTTCGG GCGGAGGATT

ScBx3_Picasso_(HG380517.1) TGAGGGGTAA AGATCTTAGG TTCCTGCCTT TTGGGTTCGG GCGGAGGATT

TaBx3B_(AB124853.1) tgaggggtaa agatcttagg ttcctgccat ttgggttcgg gcggaggatt

TaBx3D_(AB124852.1) tgaggggtaa ggatcttagg ttcctgccat ttgggttcgg gcggaggatt

TaBx3A_(AB042628.1) tgaggggtaa ggatcttagg ttcctgccat ttgggttcgg gcggaggatt

ZmBx3_(X81829.1) tgaagggcaa ggacctcagg ttcatcccgt tcggctccgg ccggaggatc

HlBx3_(AY462228.1) tgaggggtaa agatcttagg ttcctgccat ttgggttcgg gcgaaggatt

ScBx4_(KF636826) TGTATGGTAA AGATATCCGG TTTGTGCCGT TTGGGGCTGG GCGGAGGATC

ScBx4_Picasso_(HG380518.1) TGTATGGTAA AGATATCCGG TTTGTGCCGT TTGGGGCTGG GCGGAGGATC

HlBx4_(AY462229.1) tgtatggtaa agatatccgg tttgtgccgt ttggggccgg gaggaggatc

TaBx4A_(AB124854.1) tgtatggcaa agatatccgg tttgtgcctt tcggtgccgg gcggaggatc

TaBx4D_(AB042627.1) tgtatggtaa agatatccgc tttgtgcctt ttggtgccgg gcggaggatc

TaBx4B_(AB124855.1) tgtatggtaa ggatatccgc tttgtgccgt ttggggccgg gcggcggatc

ZmBx4_(X81827.1) tgtacggcaa ggacatccgg ttcgtgccgt tcggggctgg gcgcaggatc

ScBx5_(KF636825) TG...GGTCA AGATTTTAAG TACCTGCCAT TTGGATCCGG ACGTAGGATT

TaBx5D_(AB124857.1) tg...ggtca agattttaag tatctgccat ttggatccgg acgtaggatt

TaBx5A_(AB042629.1) tg...ggtca agattttaag tatctgccat ttggatccgg acgcaggatt

HlBx5_(AY462230.1) tg...ggtca agattttaag tacctgccat ttggatccgg gcgaagaatc

TaBx5B_(AB124856.1) tg...ggtca agattttaag tatcttccat ttggatccgg acgcaggatt

ZmBx5_(X81830.1) gc...ggcca ggacttcagg tacctgccgt tcgggtctgg ccgccggatc

ScBx5_Picasso_(HG380519.1) TG...GGTCA AGATTTTAAG TACCTGCCAT TTGGATCCGG ACGTAGGATT

1501 1550

ScBx2_L318_(KF620524) TGTCCTGGGG TTCACTCAGC ATCAGCAACG GTCGAGACAA TGCTGGCAAA

ScBx2_(JX442061.1) tgtcctgggg ttcactcagc atcagcaacg gtcgagacaa tgctggcaaa

ScBx2_Picasso_(HG380516.1) TGTCCTGGGG TTCACTCAGC ATCAGCAACG GTCGAGACAA TGCTGGCAAA

ZmBx2_(X81831.1) tgccctgggg tccactcggc gtccgcgacc atcgaggcga tgctgtccaa

HlBx2_(AY462227.1) tgccctgggg ttcactcggc atcagcaacg gtcgagacaa tgctggcaaa

TaBx2A_(AB042630.1) tgccctgggg ttcactcggc atcagcaacg gtcgagacaa tgctggcaaa

TaBx2B_(AB042631.1) tgccctgggg ttcactcggc atcagcaacg gtcgagacaa tgctggcaaa

TaBx2D_(AB124851.1) tgccctgggg ttcactcggc atcagcaacg gtcgagacaa tgctggcaaa

ScBx3_(KF636827) TGTCCAGGGA TGAATTTCGG GTTCGCCACC ATGGAGGTTA TGTTAGCAAA

ScBx3_Picasso_(HG380517.1) TGTCCAGGGA TGAATTTCGG GTTCGCCACC ATGGAGGTTA TGTTAGCAAA

TaBx3B_(AB124853.1) tgtccaggga tgaatttcgg gttcgccacc atggaggtta tgttagcaaa

TaBx3D_(AB124852.1) tgtccaggga tgaatttcgg gttcgccacc atggaggtta tgttagcaaa

TaBx3A_(AB042628.1) tgtccaggga tgaattttgg gttcgccacc atggaggtta tgttagcaaa

ZmBx3_(X81829.1) tgccccggga tgaacttcgg cttcgcgacc atggaggtca tgctggccaa

HlBx3_(AY462228.1) tgtccaggca tgaatttcgg gttcgccacc atggagatta tgttggcaaa

ScBx4_(KF636826) TGCGCGGGGG CTACTTTCGC GATCGCTACT GTTGAGGTGA TGCTGGCAAA

ScBx4_Picasso_(HG380518.1) TGCGCGGGGG CTACTTTCGC GATCGCTACT GTTGAGGTGA TGCTGGCAAA

HlBx4_(AY462229.1) tgcgctgggg ctaccttcgc catcgctact gtggaggtaa tgctggcaaa

TaBx4A_(AB124854.1) tgcgcggggg ctactttcgc gatcgctact gttgaggtaa tgctggcgaa

TaBx4D_(AB042627.1) tgcgcgggag ctactttcgc gatcgctact gttgaggtaa tgctggcaaa

TaBx4B_(AB124855.1) tgcgcgggtg ctactttcgc gatcgctact gttgaggtaa tgctggcaaa

ZmBx4_(X81827.1) tgcgcggggg ccacgttcgc catcgccacc gtcgagatca tgctcgcgaa

ScBx5_(KF636825) TGCCCCGGGG CAAATTTCGC GCTCGCGACA ATGGAGATCA TGCTGGTAAA

TaBx5D_(AB124857.1) tgccccgggg caaattttgc gctcgcgaca atggagatta tgctagtaaa

TaBx5A_(AB042629.1) tgccccgggg caaatttcgc acttgcaaca atggagatca tgctagtaaa

HlBx5_(AY462230.1) tgtcccgggg cgaatttcgc gcttgcgacc atggagatta tgctagtaaa

TaBx5B_(AB124856.1) tgccccgggg caaatttcgc actcgcgacc atggagatta tgctcgtaaa

ZmBx5_(X81830.1) tgccccgggg ccaacttcgg gctcgcgacc atggagatca tgctcgccaa

ScBx5_Picasso_(HG380519.1) TGCCCCGGGG CAAATTTCGC GCTCGCGACA ATGGAGATCA TGCTGGTAAA

1551 1600

ScBx2_L318_(KF620524) CCTCATGTAC CGATTCGATT GGAAGCTTCC ACCCGGATTG ......AAGG

ScBx2_(JX442061.1) cctcatgtac cgattcgatt ggaagcttcc acccggattg ......aagg

ScBx2_Picasso_(HG380516.1) CCTCATGTAC CGATTCGATT GGAAGCTTCC ACCCGGATTG ......AAGG

ZmBx2_(X81831.1) cctcatgtac cggttcgact ggcagctgcc ggcggggatg ......aagg

HlBx2_(AY462227.1) cctcatgtac cgattcgact ggaagctccc acccggatta ......aagg

TaBx2A_(AB042630.1) cctcatgtac cgtttcgatt ggaagcttcc acccggattg ......aagg

TaBx2B_(AB042631.1) cctcatgtac cgattcgatt ggaagcttcc acccggattg ......aagg

TaBx2D_(AB124851.1) cctcatgtac cgattcgatt ggaagcttcc acccggattg ......aagg

ScBx3_(KF636827) TCTCATGTAC CATTTTGATT GGGATGTTCC GAATATCATG ...GGTACCG

ScBx3_Picasso_(HG380517.1) TCTCATGTAC CATTTTGATT GGGATGTTCC GAATATCATG ...GGTACCG

TaBx3B_(AB124853.1) tctcatgtac cattttgatt gggatgttcc gaatatgatg ...ggtaccg

TaBx3D_(AB124852.1) tctcatgtac cattttgatt gggatgttcc gaatatggtg ...ggtaccg

TaBx3A_(AB042628.1) tctcatgtac cattttgatt gggatgttcc aaatatggtg ...ggtaccg

ZmBx3_(X81829.1) tctcatgtac cattttgatt gggaggttcc g......... ...ggctctg

HlBx3_(AY462228.1) cctcatgtac catttcgatt gggatgttcc gaatatgatg ...ggtaccg

ScBx4_(KF636826) CCTCATATAC CATTTCGACT GGGCGCTTCC AAGTGAGATG GAGGCCATTG

ScBx4_Picasso_(HG380518.1) CCTCATATAC CATTTCGACT GGGCGCTTCC AAGTGAGATG GAGGCCATTG

HlBx4_(AY462229.1) cctcatatac catttcgact gggagcttcc gagtgagatg gaggccgttg

TaBx4A_(AB124854.1) cctcatatac catttcgact gggagcttcc gagtgagatg gaggccattg

TaBx4D_(AB042627.1) cctcatatac catttcgact gggagcttcc gagtgagatg gaggccattg

TaBx4B_(AB124855.1) cctcatatac catttcgact gggagcttcc cagtgaaatg gaggccattg

ZmBx4_(X81827.1) cctcatctac catttcgact gggagatgcc cgccgagatg gagcgcaccg

ScBx5_(KF636825) TCTCATGTAC CATTTTGACT GGGAGGTTCC CAATGAGAAA GATGGTACCG

TaBx5D_(AB124857.1) tctcatgtac cattttgact gggaggttcc caatgagaaa gagggtactg

TaBx5A_(AB042629.1) tctcatgtac cattttgatt gggaggttcc caatgagaga gagggtactg

HlBx5_(AY462230.1) tctcatgtac cattttgact gggaggttcc caatgagaaa gagggtactg

TaBx5B_(AB124856.1) cctcatgtac cattttgact gggaggttcc caatgagaaa gatggtgctg

ZmBx5_(X81830.1) cctcatgtac catttcgact gggaggtccc caatgagaag gaagacggtt

ScBx5_Picasso_(HG380519.1) TCTCATGTAC CATTTTGACT GGGAGGTTCC CAATGAGAAA GATGGTACCG

1601 1650

ScBx2_L318_(KF620524) AAGAGGACAT AGATATGACT GAGGTGTTTG GAATAACAGT TTCAAGAAAG

ScBx2_(JX442061.1) aagaggacat agatatgact gaggtgtttg gaataacagt ttcaagaaag

ScBx2_Picasso_(HG380516.1) AAGAGGACAT AGATATGACT GAGGTGTTTG GAATAACAGT TTCAAGAAAG

ZmBx2_(X81831.1) cggaggacgt ggacatgacc gaggtgtttg ggatcacggt ctcgaggaag

HlBx2_(AY462227.1) aagaggacat agatatgact gaggtgtttg gaataacagt ttcaagaaag

TaBx2A_(AB042630.1) aagaggacat agatatgact gaagtgtttg gaataacagt ttcaagaaag

TaBx2B_(AB042631.1) aagaaaacat agatatgact gaggtgtttg gaataacagt ttcaagaaag

TaBx2D_(AB124851.1) aagaaaacat agatatgact gaggtgtttg gaattacagt ttcaagaaag

ScBx3_(KF636827) GCGCAGGGGT TGACATGGCC GAGTCGTTCG GGTTGACGCT TCGCCGGAAG

ScBx3_Picasso_(HG380517.1) GCGCAGGGGT TGACATGGCC GAGTCGTTCG GGTTGACGCT TCGCCGGAAG

TaBx3B_(AB124853.1) gtgcaggggt tgatatggct gagtcgttcg ggttaacgct tcgccgaaag

TaBx3D_(AB124852.1) gcgcaggggt tgatatggcc gagtcgttcg ggttaacgct tcgccgaaag

TaBx3A_(AB042628.1) gcgcaggggt tgatatggct gagtcgttcg ggttaacgct tcgccgaaag

ZmBx3_(X81829.1) gagcaggggt gtccatggag gagtcctttg ggctgactct tcgccggaag

HlBx3_(AY462228.1) gtgcaggggt tgatatggct gagtcgttcg ggttgacgct tcgccggaag

ScBx4_(KF636826) GCGCAAAGGT TGATATGTCC GATCAGTTTG GGATGACGCT TCGCCGAACT

ScBx4_Picasso_(HG380518.1) GCGCAAAGGT TGATATGTCC GATCAGTTTG GGATGACGCT TCGCCGAACT

HlBx4_(AY462229.1) gcgcgaaggt tgatatgtcc gatcagtttg ggatgacgct tcgccgaacg

TaBx4A_(AB124854.1) gcgcaaaggt tgatatgtca gaccagtttg ggatgaccct tcgccgaacg

TaBx4D_(AB042627.1) gcgcaaaggt tgatatgacg gaccagtttg ggatgacgct tcgccgaacg

TaBx4B_(AB124855.1) gcgcaaaggt tgatatgtcc gatcagtttg ggatgacgct tcgccgaacg

ZmBx4_(X81827.1) gggccaaggt ggacatgtcc gatcagttcg ggatgacgct tcgccggacg

ScBx5_(KF636825) GTGGGAAGGT TAGTATGGCC GAGACGTTCG GGCTGATGCT TCGCCGAAAT

TaBx5D_(AB124857.1) gtggaaaggt tagtatggcc gagacgttcg ggctgatgct tcgccgaaat

TaBx5A_(AB042629.1) gtggaaaggt tagtatggcc gagacgttcg ggctgatgct tcgccgaaat

HlBx5_(AY462230.1) gtggaaaggt tagtatggct gagacatttg ggctgatgct tcgccgaaat

TaBx5B_(AB124856.1) gtggaaaggt tagtatggct gagacgttcg ggctgatgct tcgccgaaat

ZmBx5_(X81830.1) gctggaaggt gagcatggac gagaagttcg ggctgatgct tcgcaggaac

ScBx5_Picasso_(HG380519.1) GTGGGAAGGT TAGTATGGCC GAGACGTTCG GGCTGATGCT TCGCCGAAAT

1651 1686

ScBx2_L318_(KF620524) GAGAAGCTCA TCTTAGTCCC CGTGGCCATG TGA...

ScBx2_(JX442061.1) gagaagctca tcttagtccc cgtggccatg tga...

ScBx2_Picasso_(HG380516.1) GAGAAGCTCA TCTTAGTCCC CGTGGCCATG TGA...

ZmBx2_(X81831.1) gagaagctgc tcctagtccc acaggctgcg tga...

HlBx2_(AY462227.1) gaaaagctca tattagtccc cgtgaccgtg tga...

TaBx2A_(AB042630.1) gaaaagctca tcttagtccc cgtgaccgca tga...

TaBx2B_(AB042631.1) gaaaagctca tcttagtccc cgtgaccgca tga...

TaBx2D_(AB124851.1) gaaaagctca tcttagtccc cgtgaccgca tga...

ScBx3_(KF636827) GAGAAGCTTC AACTTGTTCC TCAGATTCCG TAA...

ScBx3_Picasso_(HG380517.1) GAGAAGCTTC AACTTGTTCC TCAGATTCCG TAA...

TaBx3B_(AB124853.1) gagaagcttc aacttgttgc tcagattccc taa...

TaBx3D_(AB124852.1) gagaagcttc aacttgttcc tcggattccc taa...

TaBx3A_(AB042628.1) gagaagcttc aacttgttcc tcagattccc taa...

ZmBx3_(X81829.1) gagaagcttc tccttgttcc aaggattgct tcctga

HlBx3_(AY462228.1) gagaagcttc aacttgttcc tcagattccc taa...

ScBx4_(KF636826) GAGAGGCTTC ACCTTGTTCC TAAAATTTAC AAATAA

ScBx4_Picasso_(HG380518.1) GAGAGGCTTC ACCTTGTTCC TAAAATTTAC AAATAA

HlBx4_(AY462229.1) cagaggcttc accttgtccc taaaatttac aaataa

TaBx4A_(AB124854.1) gagaggcttc accttgttcc taaaatttac aaataa

TaBx4D_(AB042627.1) gagaggcttc accttgttcc caaaatttac aaataa

TaBx4B_(AB124855.1) gagaggcttc accttgttcc taaaatttac aaataa

ZmBx4_(X81827.1) cagaaacttt accttgttcc tagaattccc aaatga

ScBx5_(KF636825) GAGAAACTCT ACCTTGTTCC AAGGATTGTC TAA...

TaBx5D_(AB124857.1) gagaaactct accttgttcc aaagattgtc taa...

TaBx5A_(AB042629.1) gagaaactct accttgttcc aaagattgtc taa...

HlBx5_(AY462230.1) gagaaactct accttgttcc aaggattgtc gaataa

TaBx5B_(AB124856.1) gagaaactct accttgttcc aaggattgcc taa...

ZmBx5_(X81830.1) gagctgctct accttgttcc tagggcgtct agc...

ScBx5_Picasso_(HG380519.1) GAGAAACTCT ACCTTGTTCC AAGGATTGTC TAA...
